# A Web-Based and Mobile Health Social Support Intervention to Promote Adherence to Inhaled Asthma Medications: Randomized Controlled Trial

**DOI:** 10.2196/jmir.4963

**Published:** 2016-06-13

**Authors:** Justin T Koufopoulos, Mark T Conner, Peter H Gardner, Ian Kellar

**Affiliations:** ^1^ School of Psychology Faculty of Medicine and Health University of Leeds Leeds United Kingdom

**Keywords:** Internet, telemedicine, social support, asthma, adherence, attrition, engagement, randomized controlled trial, online community, social health network

## Abstract

**Background:**

Online communities hold great potential as interventions for health, particularly for the management of chronic illness. The social support that online communities can provide has been associated with positive treatment outcomes, including medication adherence. There are few studies that have attempted to assess whether membership of an online community improves health outcomes using rigorous designs.

**Objective:**

Our objective was to conduct a rigorous proof-of-concept randomized controlled trial of an online community intervention for improving adherence to asthma medicine.

**Methods:**

This 9-week intervention included a sample of asthmatic adults from the United Kingdom who were prescribed an inhaled corticosteroid preventer. Participants were recruited via email and randomized to either an “online community” or “no online community” (diary) condition. After each instance of preventer use, participants (N=216) were required to report the number of doses of medication taken in a short post. Those randomized to the online community condition (n=99) could read the posts of other community members, reply, and create their own posts. Participants randomized to the no online community condition (n=117) also posted their medication use, but could not read others’ posts. The main outcome measures were self-reported medication adherence at baseline and follow-up (9 weeks postbaseline) and an objective measure of adherence to the intervention (visits to site).

**Results:**

In all, 103 participants completed the study (intervention: 37.8%, 39/99; control: 62.2%, 64/117). MANCOVA of self-reported adherence to asthma preventer medicine at follow-up was not significantly different between conditions in either intention-to-treat (*P*=.92) or per-protocol (*P*=.68) analysis. Site use was generally higher in the control compared to intervention conditions.

**Conclusions:**

Joining an online community did not improve adherence to preventer medication for asthma patients. Without the encouragement of greater community support or more components to sustain engagement over time, the current findings do not support the use of an online community to improve adherence.

**ClinicalTrial:**

International Standard Randomized Controlled Trial Number (ISRCTN): 29399269; http://www.isrctn.com/ISRCTN29399269/29399269 (Archived by WebCite at http://www.webcitation.org/6fUbEuVoT)

## Introduction

Online communities have the potential to foster feelings of social support in patients battling chronic health issues [1-3]. The management of chronic health issues falls mostly on the patient and their family and can create a sense of isolation and distress [4]. Although there is no single accepted definition of an online community, a common definition is “...a group of people who share a strong common interest, form relationships, and interact online” [1]. Some of the largest online communities for patients (as of November 2015) are MedHelp, PatientsLikeMe, TuDiabetes, and DailyStrength with millions of members [5]. Such online communities can provide valuable support, but do they also relate to better management of chronic health issues such as increased medication adherence?

### Link Between Social Support and Medication Adherence

Medication adherence can be defined as the extent to which a patient follows medication-taking guidelines agreed on by a patient and doctor [6]. Common guidelines include medication dosage and frequency. According to the World Health Organization, adherence to asthma medicine is just 50%, representing a significant health threat including increased risk of hospitalization and death [4]. Adherence is a complex phenomenon influenced by many factors, including economic (eg, financial costs of drugs or therapies), social (eg, age, race), therapy (eg, adverse reactions to medication), and patient (eg, individual attitudes and concerns) factors [4,6].

Adherence to medication is also influenced by social support. Although many of the factors relating to adherence are difficult to influence, social support is a promising target because it may be improved through low cost interventions (eg, support groups, sponsors). In a systematic review of 122 studies published between 1948 and 2001, DiMatteo [7] found a significant relationship between social support and adherence. Studies were categorized into types of support, including practical support (eg, instrumental support, assistance, reminders, organization, support for a specific behavior), emotional support, unidimensional social support (involving multiple types of social support, not separated in their measurement), family cohesiveness (eg, warmth, closeness, acceptance), and marital status and living arrangement. Patients receiving practical support were 3.6 times more likely to adhere to treatment regimens than those who were not. Risk of nonadherence was also found to be 1.53 times more likely if patients had low social support. Equivalence between online and face-to-face interventions in related fields suggests that online support could similarly provide social support and be associated with improved medication adherence [8].

### Current State of the Evidence for Online Communities: Randomized Controlled Trials

There have been only a few randomized controlled trials (RCTs) of online communities for patients with chronic health issues [9-13]. The results of these studies have been mixed. For example, a randomized controlled trial by Richardson et al [9], found an online community for an Internet-mediated walking program did not increase participant step count, but participants randomized to the online community had greater engagement and lower rates of attrition than the control group.

Similarly, a RCT by Brindal et al [10] of an online platform for weight loss found that compared to the noninteractive control group, groups with online community features (eg, friend requests, newsfeeds, quizzes, profile pages) had greater engagement, but did not show increased weight loss or retention. A RCT by Stoddard et al [11] also found that an online community feature (a message board) appeared to increase engagement for a smoking cessation website, but this feature did not influence quit rates.

Although more broadly fitting the definition of an online community [1], we found two trials that used Facebook to evaluate the effectiveness of online communities on physical activity, and weight loss, respectively [12,13]. In a RCT to evaluate the feasibility and efficacy of a Facebook-based intervention on physical activity for young adult cancer survivors, participants were randomized to either a Facebook online community intervention condition (FITNET) or a Facebook self-care condition. Increases in light physical activity were more than 2 hours per week greater in the FITNET condition compared to the self-care condition [12]. In another trial using Facebook, students with a body mass index of 25-50 kg/m^2^were randomized to Facebook, a Facebook plus text messaging and personal feedback group, or a wait list. After 8 weeks, the Facebook plus text messaging and personal feedback group had significantly greater weight loss than either of the other two groups [13].

### Theoretical Framework

We predicted that participating in an online community would lead to greater medication adherence. The theoretical underpinnings of this prediction are Social Cognitive Theory [14,15], the Theory of Planned Behavior (TPB) [16], and the stress and coping perspective of social support [17]. The effect of community website exposure [11] is also included in the theoretical framework of the intervention. According to Social Cognitive Theory [14,15], individuals can learn by observing the actions of others. If those actions produce an effect that is beneficial to the individual being observed, those actions are more likely to be imitated. It was predicted that participants observing the adherence of other patients will themselves improve adherence to inhaled corticosteroid (ICS) treatment. Participants reading other patients’ success stories regarding adherence or dealing with asthma more generally will learn from these stories and apply these lessons to their own life, improving adherence.

The TPB states that the more an individual intends to perform a given behavior, the more likely they are to perform that behavior [16]. According to the TPB, intentions to perform a behavior are based on norms, attitudes, perceived behavioral control, and influence whether a given behavior is performed through intentions. It is predicted that when patients observe self-reports of adherence and other interactions on the site, over time these observations will positively influence their attitudes, norms, and perceived behavioral control around adherence promoting stronger intentions to adhere and subsequent adherence behavior.

Social support has been defined as the quality and structure of an individual’s relationships; greater social support is associated with improvements in adherence to medication regimens [7]. As is predicted in the stress-buffering perspective of social support [17], the perception of having socially supportive relationships and the support that participants actually receive will reduce stress associated with adherence and asthma, improving adherence and overall health.

### Objective and Hypothesis

Our objective was to conduct a rigorous study of the effects of participating in an online community on adherence to asthma medicine. To our knowledge, there are no previous studies of the relationship between online communities and medication adherence. Asthma was chosen as the target illness for this trial because medication adherence is often low and the incidence of chronic asthma in adults is relatively high: nearly 10% in the United Kingdom [18]. Asthma in adults is typically treated with a combination of an ICS preventer and a bronchodilator reliever. We hypothesized that adherence would be improved by participation in an online community due to processes of modeling and social support.

## Methods

### Study Design

In this 2-arm RCT, participants were enrolled at random into either the intervention condition, “AsthmaVillage,” an online community for patients with asthma, or the control condition, “AsthmaDiary,” an online diary for recording ICS preventer use. Intervention arm participants had access to an online community and could leave comments or see who else was online. In contrast, the control-arm participants could not read the posts of other control-arm participants or interact with other participants online. An active control was used to test the effect of the community on adherence and to prevent participants from guessing if they were in the group of interest. The study was carried out for 9 weeks, between June 24 and August 26, 2013. The trial conformed to the Consolidated Standards of Reporting Trials (CONSORT) eHealth Checklist (Multimedia Appendix 1) [19].

### Recruitment

A total of 1833 emails requesting participants for a study on asthma management were sent out to department secretaries of the 40 largest universities in the United Kingdom by enrollment. Universities were chosen as recruitment sites because recruitment through medical centers or primary care practices in the United Kingdom requires a lengthy approvals process that can go on for many months, which was beyond the resources available for this study.

Recruitment emails were sent over a period of 10 days from June 13 to 23, 2013. Department secretaries were asked in the body of the email to forward the request to department mailing lists (Multimedia Appendix 2). The request for participants invited individuals managing their asthma with an ICS preventer to fill out an eligibility screening form and included a link to the questionnaire (Multimedia Appendix 3). Participants were also informed that on successful completion of the study they would receive a £20 (approximately US $30) shopping voucher. Successful completion of the study was defined as recording their ICS preventer use at least once per week on the site for the duration of the study.

### Eligibility Screening and Consent

**Figure 1:**
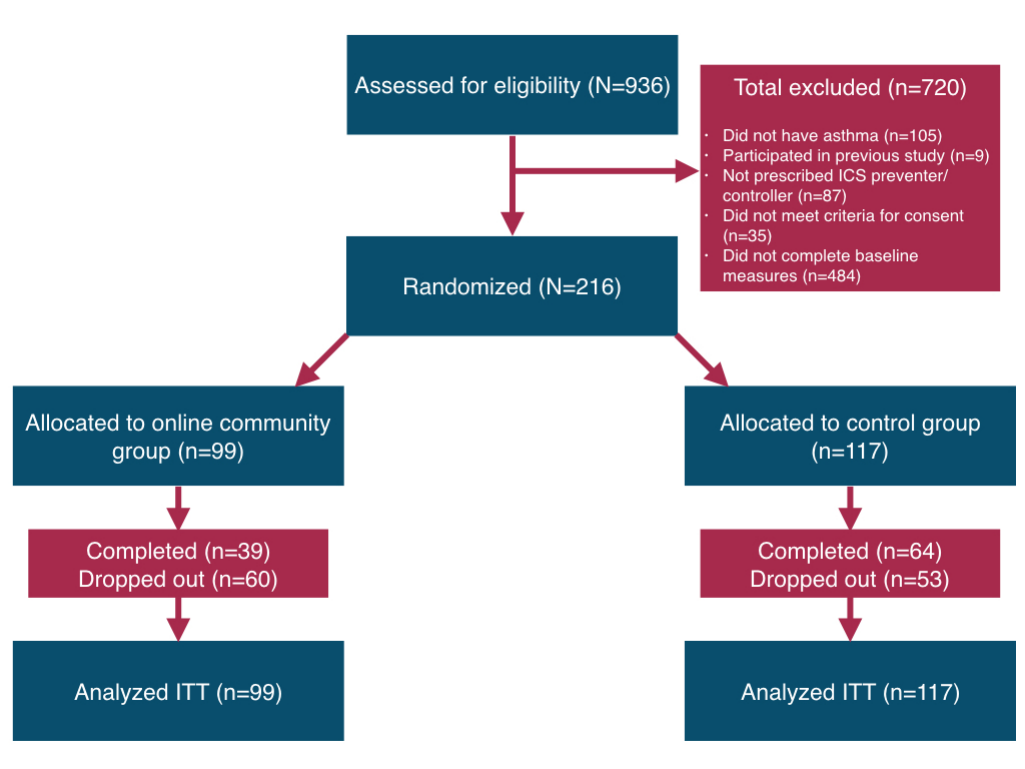
Participant flowchart of recruitment, participation after exclusion criteria, randomization, and attrition.

A total of 936 participants responded to the eligibility questionnaire. Participants were excluded from the study if they failed to complete the eligibility questionnaire (n=256) or baseline measures (n=228), did not have asthma (n=105), were not prescribed an ICS preventer inhaler for a weekly regimen of at least one dose per week (n=87), failed to complete informed consent (n=35), or had previously participated in the pilot study (n=9). After screening, a total of 251 participants were eligible for study inclusion. See Figure 1 for details.

Participants were automatically taken to the information sheet (Multimedia Appendix 4) and were asked to provide informed consent (Multimedia Appendix 5). In all, 35 participants refused to provide consent and were eliminated from the study, leaving 216 eligible participants. Participants were then randomized to the diary (n=117) and online community (n=99) conditions. Randomization occurred through a random number generator [20], yielding two unequal groups. The experimenters then manually separated the two lists and emailed both groups log-in instructions.

The online screening survey was administered through Qualtrics [21], a subscription-based online survey software suite.

### Baseline Measures

Baseline measures were included as part of the eligibility screening. Participants completed an online survey (Multimedia Appendix 6) that included questions about gender, age, previous social networking use, and prescriptions.

Preventer adherence in both conditions was self-reported using the 6-item Simplified Medication Adherence Questionnaire (SMAQ) [22] (Multimedia Appendix 7), a common, validated measure of medication adherence. A self-report measure of medication adherence was used because this study was designed to be low cost, feasible, and widely geographically distributed in the United Kingdom. Furthermore, questionnaires can have a high concordance with more expensive objective measures, such as electronic counters [23]. In the questionnaire, the SMAQ refers generally to all medicine. For example, the first item of the SMAQ is “Do you ever forget to take your medicine?” For this study, all instances of the word “medicine” were changed to “asthma preventer medication.” This small change was unlikely to have affected the measure.

The SMAQ was then recalculated with dichotomous scoring of all variables (more than two missed uses was treated as nonadherent) and by reverse scoring of item 4 of the SMAQ (“Thinking about the last week, how often have you not taken your asthma preventer medicine as prescribed?”). The rescored SMAQ formed a reliable measure (Cronbach alpha=.72); therefore, a mean across all completed items was computed. If there were two or fewer completed SMAQ items for an individual, the score was treated as missing (ie, no value was given) and these individuals were excluded from the analysis involving this variable. Replacing missing values with imputed values did not substantively alter the reported findings.

### Pilot Study

Previous to this trial, a small pilot study (N=8) was conducted to gather qualitative feedback on the usability of the online community. The online community was created using WordPress [24], an open-source content management system, and BuddyPress [25] social networking features (Multimedia Appendix 8). There were three primary intervention components: (1) a home page that displayed all site activity in a rolling status board, (2) a group diary for posting preventer use, and (3) a profile page. The intervention was developed and adapted based on feedback collected during the pilot study. No additional feedback was collected during the trial.

The pilot study ran for 31 days. Participants were required to post their preventer use weekly in the online community. At the end of the study, participants were sent a questionnaire with short-answer items regarding the usefulness and usability of the site. Participants reported that they found the social support features of the intervention useful in connecting with other asthma patients, but found that after the first few weeks it was difficult to become engaged with the site because the number of active conversations diminished.

The results of the pilot study influenced the development of the final intervention administered during the RCT. The main finding from the pilot study was that participants found it difficult to engage with the site because of the lack of activity. With only eight members, participant conversations fell off rapidly and little discussion was observed after the first week. Participants reported logging in to the website to report their medication usage and then logging out right after with little else happening on the site to engage them. However, most of the participants reported that hearing about other asthma sufferers’ experiences and having a forum to ask questions about asthma was useful. To more directly engage participants, a separate site section dedicated to questions about asthma was created for the RCT.

Additionally, participants reported often failing to remember to log in to AsthmaVillage, which likely also affected site usage. Therefore, for the RCT we decided to implement a system of automated, weekly reminder notifications. This automation was accomplished with MailChimp, an email marketing software tool.

### Intervention

Like the pilot, the online community was created using WordPress and BuddyPress. In addition to the three features mentioned in relation to the pilot study, a fourth feature was added based on the results of the pilot and the need to increase engagement: a page for posting questions and answers about asthma.

The main actions participants could take on the online community were reporting their preventer use and writing posts, comments, or questions. Questions and comments needed to be answered by the community members themselves because there was no experimenter intervention once the trial had begun. The only feedback patients could receive during the trial was from other patients themselves because this intervention was optimized for implementation at scale and at low cost. This trial attempted to understand the value of an online community, implemented without the added support of a community manager to engage members. The intervention was developed to be accessible by smartphone Web browsers as well as by desktop versions.

The effect of membership in the online community is dependent on the extent members use the website of the online community itself. As previously mentioned, site engagement was a barrier for participants in the pilot study. In order to create a website that was more engaging, we created a site discussion section dedicated to posting questions and answers regarding asthma. Such features have been shown to be beneficial to engagement in previous studies [9].

### Control

The control condition comprised an online diary, AsthmaDiary. The online diary was created using Google Forms. A single-item survey was created (Multimedia Appendix 9): “How many times did you take your preventer?” Participants randomized to the control condition could then input the number of puffs and, after entering their unique personal identification number (PIN), hit “submit.” Because participants did not need to log in with a username to fill out the form, participants used a PIN that allowed their posts to be identified by the researcher. Participants in the control condition could not see the posts of the other participants or otherwise know that there were other participants posting in their condition.

### Follow-Up Measures

Follow-up measures were taken 9 weeks postbaseline. The SMAQ was also used to measure ICS preventer adherence at follow-up (SMAQ-T2) and scored in an identical fashion. The SMAQ-T2 formed a reliable measure (Cronbach alpha=.69) and mean scores were computed across all completed items. Missing values were assigned to any participant with fewer than two filled-out SMAQ-T2 items. Intention-to-treat (ITT) analyses used SMAQ-T1 means carried forward to follow-up for participants who did not complete the follow-up SMAQ-T2 measures.

Site activity was measured by producing counts of comments and posts by user and week. Comments were exported from the WordPress content management system and categorized into the following groupings: “preventer posts” (posts that were self-reports about preventer use), “posts about symptoms” (posts made by users about their asthma symptoms), “questions about asthma” (questions about asthma embedded in a comment or post, or standalone), “answer or reply comments” (answers or comments left by users to posted questions), and “nonanswer comments” (comments or statements left by users that were neither about symptoms or supporting another user). Posts or comments were categorized at the sentence level because longer posts or comments often had statements about symptoms and follow-up questions in the same post.

Participants in both conditions were instructed to report their preventer use on their assigned website each time they used their preventers over a period of 9 weeks. The extent that participants adhered to these directions was calculated (site adherence). The total number of preventer puffs (total puffs) each participant reported taking in each week was divided by the total number of preventer puffs prescribed in a given week (daily total number of puffs prescribed in 1 day multiplied by 7). This calculation produced a score of weekly site adherence for each participant. Thus, if a participant logged their preventer use every time they were prescribed to use their preventer, their site adherence would be 100%. The mean adherence across each of the 9 weeks was calculated, forming an overall score of adherence to the site (site adherence).

### Procedure

After randomization, participants were emailed instructions on how to use their site (Multimedia Appendices 10 and 11). Participants were then prompted to log in to their respective websites. Before logging in, participants created a username and password for their sites. Participants randomized to the diary created a PIN that they were required to input whenever they posted preventer use. Participants in both conditions were quasi-anonymous. Registration required an email confirmation. It was not possible to determine whether an individual operated multiple accounts on AsthmaVillage or the online diary. Account passwords were screened by the experimenters for duplicates in an attempt to mitigate this possibility.

During the trial, an automated weekly email was sent to participants indicating which week the trial was on (eg, week 6 of 9) and with a reminder to post their preventer inhaler use (Multimedia Appendix 12). At the end of the study, participants were emailed instructions (Multimedia Appendix 13) on how to complete the follow-up measures.

Participants who completed the study and posted on their site at least once a week were mailed a £20 (approximately US $30) shopping voucher for participation (n=82). Participants who completed at least the baseline and follow-up measures were mailed a £10 (approximately US $15) voucher (n=23). This approach was taken to allow as many participants as possible an opportunity to fill out the follow-up questionnaire and reduce bias in the sample.

### Statistical Analysis

#### Sample Size Calculation

Based on an expected medium effect size (*d*=.5), an alpha of .05 (1-tailed), and power of 80%, we calculated that a total of 102 participants would be needed to complete the study. A medium effect size was justified based on the review by Webb et al [26], which found that greater use of theory in Internet interventions was associated with larger effect sizes. For example, use of the TPB was associated with a medium-sized effect (*d*=.5) in this review [26].

In the few RCTs of online communities, attrition varied considerably. We assumed a 50% dropout rate and, thus, aimed to recruit double the number of participants the power analyses suggested were required.

#### Analysis

First, descriptive statistics were calculated for all variables (gender, age, total puffs, site adherence, SMAQ-T1, SMAQ-T2, and SMAQ-T2 ITT) and examined across the whole sample and for each condition to ensure the measures were normally distributed.

Next, we examined the effect of condition on outcome variables. Multivariate analysis of variance (MANOVA) was used when variables were not measured at baseline (total puffs and site adherence). Multivariate analysis of covariance (MANCOVA) was used when variables were also measured at baseline (SMAQ) with the baseline score being the covariate.

Statistical analyses were conducted using SPSS version 20.0 (IBM Corp, Armonk, NY, USA).

### Human Participants and Trial Registration

The University of Leeds, School of Psychology Ethics Committee approved this study (ethics reference number 13-0096). All participants gave online consent. The details of the trial were made public in advance (ISRCTN trial registration number: 29399269).

## Results

### Attrition

Of the 216 participants who met our inclusion criteria, only 103 participants fully completed the study, 64 (62.1%) of these were from the control arm. Of the 99 participants allocated to the intervention arm, 82 created a username and password (83%). A chi-square test indicated that dropout was higher in the intervention condition (60/99, 61%) than the control condition (53/117, 45.3%; χ^2^_2_=5.0, *P=*.03).

We also tested whether the sample who completed the study were representative of the initial sample on baseline measures of gender, age, and SMAQ-T1. MANOVA revealed a significant difference between groups (Wilks’ lambda=0.944, *F*_3,212_=4.190, *P=*.007). Examination of the univariate effects revealed significant effects for SMAQ-T1 (*F*_1,214_=4.48, *P*=.04) and gender (*F*_1,214_=7.20, *P*=.008), but no effect for age (*F*_1,214_=0.72). On average, completers scored higher on the SMAQ-T1 (mean 1.49, SD 0.30) than noncompleters (mean 1.40, SD 0.27). Higher SMAQ scores indicate lower preventer adherence. Study completers were also more likely to be female (79.6%, 82/103) than noncompleters (61.1%, 69/113).

Our attrition analyses indicated that the sample completing the study was not fully representative of those starting the study; therefore, our analyses based on completers should be treated with caution.

### Descriptive Statistics

Participants were mostly women and although ages ranged from 18 to 64 years, the average participant was in their late twenties (Table 1). There was generally an even mixture of adherent and nonadherent participants. Table 1 also shows that, in general, the control and intervention arms showed few differences except in relation to site adherence measures.

**Table 1:** Descriptive statistics.

Variable		Total N=216	Control n=117	Intervention n=99
Age (years), mean (SD)		28.1 (9.7)	28.8 (10.1)	27.2 (9.2)
**Gender, n (%)**				
	Male	64 (29.6)	35 (29.9)	29 (29.3)
	Female	151 (69.9)	82 (70.1)	69 (69.7)
**Self-report adherence, mean (SD)**				
	SMAQ-T1	1.45 (0.29)	1.48 (0.30)	1.41 (0.27)
	SMAQ-T2^a^	1.48 (0.28)	1.49 (0.29)	1.46 (0.28)
	SMAQ-T2 ITT	1.44 (0.28)	1.46 (0.29)	1.42 (0.26)
**Site measures, mean (SD)**				
	Site adherence^b^	18.35 (21.98)	25.53 (25.63)	9.22 (10.87)
	Total puffs^c^	42.32 (50.40)	54.49 (58.01)	21.20 (20.80)

^a^For SMAQ-T2, total n=104, control n=64, and intervention n=40.

^b^For site adherence, total n=134, control n=75, and intervention n=59.

^c^For total puffs, total n=134, control n=85, and intervention n=49.

### Primary Outcomes

MANCOVA indicated no condition effects on the SMAQ scores (Wilks’ lambda=0.998; *F*_1,96_=0.176, *P=*.68). For this analysis, 104 participants were included, with 64 in the control and 40 in the intervention groups. When based on ITT analyses, MANCOVA also indicated no condition effects for the SMAQ, with 117 participants in the control and 99 participants in the intervention groups (Wilks’ lambda=1.000; *F*_1,207_=0.011 *, P* =.92). MANOVA for variables not measured at baseline (site adherence, total puffs) revealed significant differences for condition (Wilks’ lambda=0.922; *F*_2,114_=4.835, *P=*.01). Examination of the univariate statistics revealed significant differences for both site adherence (*F*_1,91_=6.635, *P*=.01) and total puffs (*F*_1,91_=9.400, *P*=.003). Examination of the means revealed that these differences reflected the higher levels of site adherence and total puffs in the control compared to the intervention condition (Table 1).

### Site Activity Via Community Posts and Comments

**Figure 2:**
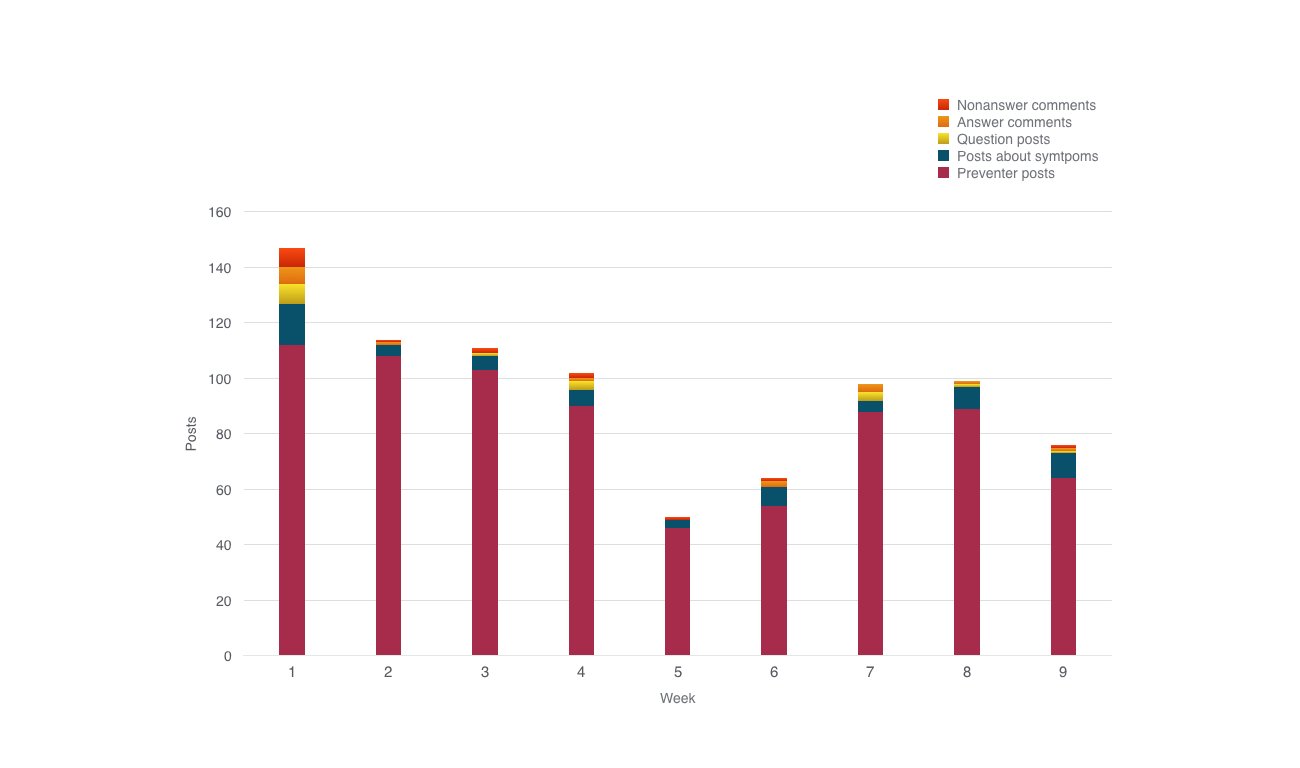
Comments and posts by type over nine weeks.

Examination of site activity via the posts and comments created by users during the 9 weeks suggested that the majority of site activity were self-reports of asthma preventer use (Figure 2). Of the 99 participants allocated to the intervention condition, 83 created a username and password.

Of the 861 comments or posts left by users on AsthmaVillage (intervention condition), 754 (87.6%) were preventer posts (eg, “2x preventer” and “1x symbiocort”). Looking at the remaining comments or posts, 7.1% (61/754) were posts about symptoms (eg, “Got my preventer back, asthma has been quite uncontrolled this past week” and “No need to take my ventolin today, feeling good ” and “Well still haven’t taken my preventer since my last post. To be honest, not feeling any different”), 1.9% (16/754) were questions (eg, “Has anyone taken Singulair? I’ve heard it’s good as a preventer and for allergies. I’ve been on fexofenadine for years and it’s starting to lose effectiveness. It would be great to take a tablet that would replace my antihistamine and my symbicort” and “I have a question about asthma reviews at the doctor. They chase me to come every 6 months, but nothing has ever changed [I’m 35 and have had asthma since I was 5, so I’m pretty good at managing it myself by now]. I don’t really understand the point of the review—if my symptoms get worse I will go to the doctor, but if it’s well managed why do I still need to go every 6 months? Does anyone else find these reviews useful? Is there something I can do to get more out of them?” and “I’m self-regulating my dosage at the moment and my symptoms are almost nonexistent during summer. I was wondering if it’s better to keep taking the preventer two puffs a day, or to stop the preventer and just use reliever before exercise? Anyone know?”), 1.7% (15/754) were answer comments (eg, “Hi, I have the same experiences. Nothing has changed in 20 years and it feels like a waste of their time. I’m keen to know if there’s anything I can do to get more out of them too.” and “Since I’ve been with my current GP I’ve had only annual reviews [and they are useful in that the preventer was altered from a pure steroid to include a long acting broncho-dilator that’s rendered Ventolin seldom used].”), and 1.7% (15/754) were nonanswer statements (eg, “Finally managed to pick up my preventer inhaler today after far too long without it.” and “I’ve got a cold...”).

In all, 33 of 82 participants (40%) posted something on the site that was not purely a preventer post (ie, posts about symptoms, questions about asthma, answer or reply comments, nonanswer comments), and there were a mean 3.24 (SD 0.94) nonpreventer posts over the 9 weeks of the intervention. Eight of 82 participants (10%) explicitly asked questions of the community and tended to post more frequently (mean number of nonpreventer posts in this subgroup was 5.38, SD 3.50).

### Site Adherence Over Nine Weeks

**Figure 3:**
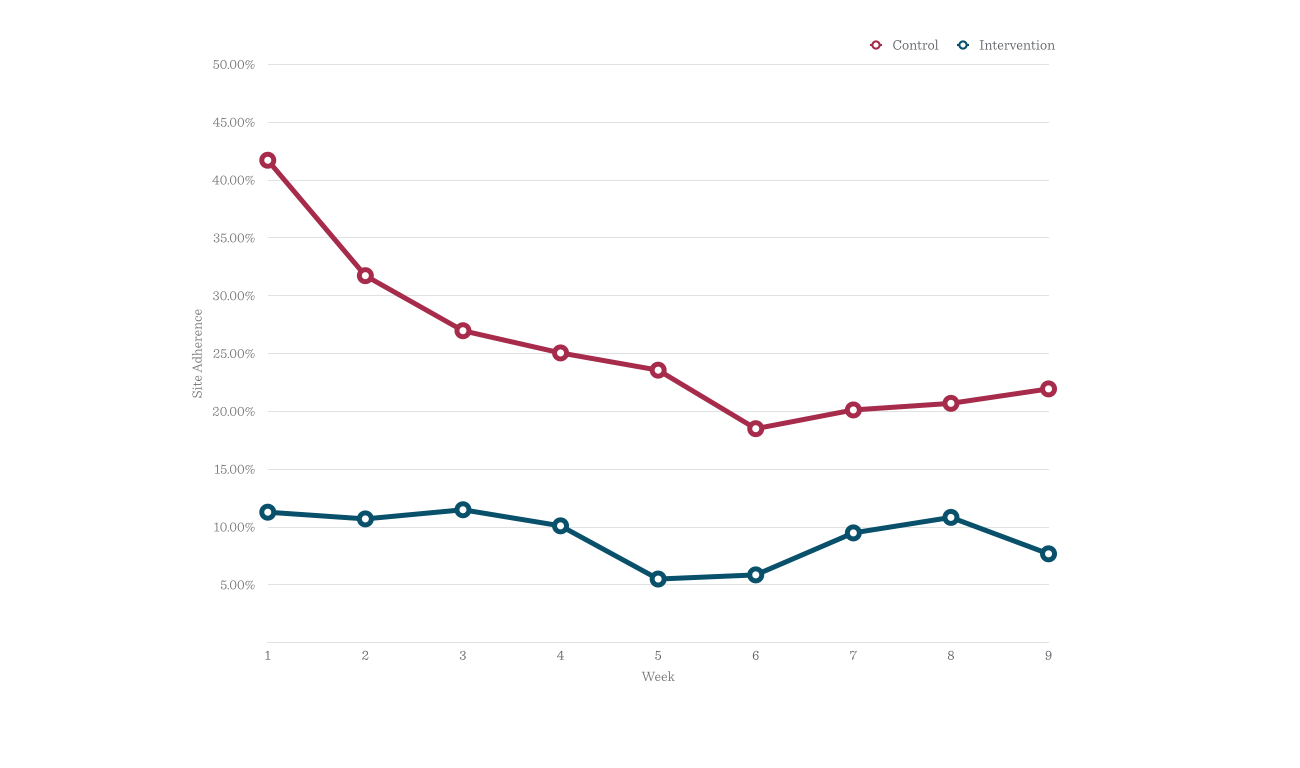
Change in site adherence over nine weeks by condition.

Further detailed examination of the weekly site adherence means over the 9 weeks of the intervention indicated a substantial difference between conditions in adherence. This was particularly apparent at week 1, with 41.7% adherence in the control condition compared to 11.3% site adherence in the intervention condition. Figure 3 shows that site adherence was most different between groups at week 1, but fell at a much greater rate across weeks in the control compared to the intervention condition. Site adherence was relatively consistent across weeks in the intervention condition.

## Discussion

### Summary of Principal Results

This RCT examined whether being part of an online community would improve self-reported preventer adherence. We anticipated that through the mechanisms of role modeling, social support, and website exposure the intervention compared to the control condition would increase adherence. However, contrary to expectations, being part of an online community for asthma patients for 9 weeks failed to increase self-reported medication adherence to ICS preventer therapy compared to a control diary condition (*P=*.68). In addition, there was significantly lower site adherence in the intervention condition than in the control condition (*P*<.001), even from the first week of the intervention (Figure 3).

Study condition also predicted attrition. Participants were less likely to complete the study if they were randomized to the intervention compared to control condition (*P=*.03). Further examination of this data indicated that both baseline adherence as assessed by SMAQ scores (*P*=.04) and gender (*P*=.008) were related to attrition with study completers being more likely to be women (79.6%, 82/103 vs 61.1%, 69/113) and less likely to be adherent to asthma preventer medication at baseline than noncompleters (mean 1.49, SD 0.30 vs mean 1.41, SD 0.27). This finding can perhaps be explained by the site being more useful for people struggling with asthma preventer adherence and less so for people without problems. Such an interpretation would be consistent with Magnezi et al [3] and their study of patient activation or the extent individuals are able to manage their own health care. These authors found a negative relationship between patient activation and perceived usefulness of a website because taking a less active role in one’s own medical care predicted higher website usefulness.

#### Recruitment and Attrition

Of the 936 participants who began the eligibility and baseline screening forms, 48.3% (452/936) completed them and, after exclusions, 23.1% (216/936) were randomized to condition. It is not unusual for Web-based studies of online communities to have a large number of participants dropout between screening and randomization. For example, Richardson et al [9] had 880 signups, but after dropouts and exclusions, only 324 were randomized. It is also possible that given the number of question items and forms that needed to be completed to be eligible for the study, participants who were initially attracted by the monetary reward became discouraged and failed to continue.

Of the 216 participants who began the trial, 103 (47.7%) completed the 9-week study. Attrition was within the typical range described by other online community RCTs [9-13] and reviews of the literature for midsized trials [26]. However, it is difficult to make comparisons because these RCTs are so different in design and virtually none tested the effectiveness of the online community as a standalone intervention as in this study.

#### Primary Outcomes

The online community did not improve self-reported preventer adherence (SMAQ) compared to the control. This finding is consistent with findings by Eysenbach et al [27] that virtual health communities were not associated with improved health outcomes and, more recently, Richardson et al [9] or Brindal et al [10] that found membership in online communities had no effect on behavior change. The evidence from these trials and the one reported here would suggest that joining an online community intervention is not associated with improved health behavior.

#### Site Activity Via Community Posts and Comments

Analysis of the comments and posts indicates that the online community was primarily used for posting asthma medication use (Figure 2). This is not entirely unexpected because posting ICS preventer use was the only requirement for the study. However, more than 100 (12%) of the 754 posts were nonpreventer and 40% (33/83) of the community could be considered as “nonlurkers.” Within the context of online health communities, “lurkers” are individuals who do not participate in posting [28]. Because these members posted something other than a preventer post, which was a study requirement, these members met the criteria for nonlurkers. Studies indicate lurking to be highly variable, between 0 and 99% [29]. Nonnecke and Preece [29] found a mean 45.5% of lurkers in online health communities. Research indicates that both lurkers and nonlurkers can receive benefits from online health communities [30]. Overall, the present online community did foster interaction between participants (eg, questions, answers to questions, posts about symptoms) and there existed a reasonable ratio of lurkers to nonlurkers.

#### Site Adherence Over Nine Weeks

Beginning in the first week of the study, there was a significant difference between conditions for site adherence. One possible explanation could be that participants did not like posting their preventer use in an online community compared to the participants posting in an online diary. Perhaps these participants felt worried or uncomfortable posting this information publicly even though their identity was anonymized. On the one hand, an online diary could maintain a sense of privacy; on the other hand, the more rapid decline in posting in the diary condition might be explained by a lack of engagement over time. Engagement remained fairly consistent in the online community perhaps because of the presence of other members. Such an explanation would also be consistent with the findings of Richardson et al [9], in which an online community was found to reduce attrition to an Internet-mediated walking program, but did not increase walking step count.

### Study Strengths and Weaknesses

This study had a number of strengths and weaknesses. In relation to strengths, this study tested the effect of membership in an online community as a single intervention component in a RCT; RCTs are the gold standard for determining the effect of an intervention on an outcome. Second, previous studies have also attempted to influence the member activity of online communities through various time-intensive posting strategies, possibly confounding their results [9,27]. In contrast, this study did not attempt to influence participation beyond the weekly reminders sent to participants in both conditions. Outside of a research context, it is improbable that organizations seeking to enhance health behaviors would divert considerable resources toward encouraging participation. As such, this study represents a test of a deliverable intervention. Third, the inclusion criteria for participation were broad, primarily requiring that patients be prescribed an ICS preventer for daily use. This increases the generalizability of the findings to a large percentage of individuals with asthma.

In relation to weaknesses, the only validated measure of adherence was a self-report measure. A self-report measure was chosen to facilitate the aims of this study, which was to deliver an intervention at low cost across a geographically widespread group of participants in the United Kingdom. Several studies have shown that self-report measures of adherence can be unreliable [31,32]. However, in an analysis of 86 published studies using both self-report and non–self-report measures, Garber et al [23] found that self-report questionnaires and diaries were the most highly concordant with electronic measures (75% agreement).

Second, previous RCTs of online communities [9-13] have involved multiple components beyond the community itself to influence health behavior change. As such, these studies are unable to isolate the effectiveness of a single component compared to another, making it difficult to determine causality of the reported effects. Although it was a deliberate choice to test only a single component for this study, reliance on a single component also made this intervention more susceptible to failure. Similar to the pilot study, it may be that when the online community was unable to foster sustained engagement, participation dropped off.

Third, the measure site adherence should also be interpreted with caution. It is not an objective measure of actual preventer adherence, but more likely a measure of adherence to the study reporting instructions. It is possible that actual preventer adherence was different from that reported by participants on either site. Such an explanation would be supported by the SMAQ-T2 ITT and SMAQ-T2 means (Table 1), which indicated that there were no significant differences in self-report adherence for either condition at follow-up.

Finally, because universities were chosen as recruitment sites, it is possible that individuals associated with universities (eg, teachers, students, staff) are more likely to have the type of economic and social stability that may be associated with better adherence, self-reported or otherwise. This may affect generalizability because the most poorly controlled asthma patients may not be well represented in our sample.

### Future Directions

The present findings indicate that a “pure” online community does not improve self-report medication adherence. Future research may wish to experiment with multiple levels of engagement with an online community. For example, researchers may have a pure community in the comparator and a community with a virtual coach in the intervention condition. Automation and machine learning could also be used predict compliance and offer different levels of automated support, such as notifications on relevant content or reminders to take medication. These types of community interventions might be better tailored to individual participants with varying levels of adherence or different attitudes around asthma and compliance. Although we believe assessing the independent value of an online community component is an important step for online health community research, without enough participants to sustain long-term discussion, pure community interventions are likely to fail. Thus, additional patient-tailored components can offer more value to patients and possibly sustain engagement over longer periods of time.

Because self-report measures of adherence are sometimes unreliable, future studies may wish to also invest in a mechanical or digital measure of adherence able to provide objective ICS preventer data that can be compared against self-report measures such as those employed here.

### Conclusions

An online community did not improve self-reported adherence to asthma preventer medicine. Surprisingly, participants were much more adherent in posting inhaler use in the control condition than the intervention condition, although it appeared that this difference between conditions attenuated over time. Without greater community support beyond the existence of the community itself, it does not seem that an online community alone can improve adherence. However, our analyses of attrition suggest that online communities may be more useful to patients with poor asthma adherence than patients with good adherence.

## Appendix

Multimedia Appendix 1 CONSORT-EHEALTH (V 1.6.1)-Submission/Publication Form.

Multimedia Appendix 2 Recruitment email.

Multimedia Appendix 3 Eligibility screening form.

Multimedia Appendix 4 Participant information sheet.

Multimedia Appendix 5 Participant Consent Form.

Multimedia Appendix 6 Baseline questionnaire.

Multimedia Appendix 7 Simplified Medication Adherence Questionnaire.

Multimedia Appendix 8 Screenshots of website homepage, group diary page, asthma Q&A page, and profile page.

Multimedia Appendix 9 Control condition diary screenshot.

Multimedia Appendix 10 Participant site instructions.

Multimedia Appendix 11 Participant site instructions.

Multimedia Appendix 12 Reminder to post preventer inhaler.

Multimedia Appendix 13 Instructions on how to complete the follow-up measures.

## Abbreviations

ICS: inhaled corticosteroid
ITT: intention-to-treat
MANOVA: multivariate analysis of variance
PIN: personal identification number
RCT: randomized controlled trial
SMAQ: Simplified Medication Adherence Questionnaire
TPB: Theory of Planned Behavior
