# Supplementary material for: A Web-Based and Mobile Health Social Support Intervention to Promote Adherence to Inhaled Asthma Medications: Randomized Controlled Trial
Source: J Med Internet Res. 2016 Jun 13;18(6):e122. doi: 10.2196/jmir.4963 (PMC4923591; doi:10.2196/jmir.4963)
Supplement: Multimedia Appendix 1 [file jmir_v18i6e122_app1.pdf]

CONSORT-EHEALTH (V 1.6.1) - Submission/Publication Form

The CONSORT-EHEALTH checklist is intended for authors of randomized trials evaluating web-based and Internet-based applications/interventions, including mobile interventions, electronic games (incl multiplayer games), social media, certain telehealth applications, and other interactive and/or networked electronic applications. Some of the items (e.g. all subitems under item 5 - description of the intervention) may also be applicable for other study designs.

The goal of the CONSORT EHEALTH checklist and guideline is to be
a) a guide for reporting for authors of RCTs,
b) to form a basis for appraisal of an ehealth trial (in terms of validity)

CONSORT-EHEALTH items/subitems are MANDATORY reporting items for studies published in the Journal of Medical Internet Research and other journals / scientific societies endorsing the checklist.

Items numbered 1., 2., 3., 4a., 4b etc are original CONSORT or CONSORT-NPT (non-pharmacologic treatment) items.
Items with Roman numerals (i., ii, iii, iv etc.) are CONSORT-EHEALTH extensions/clarifications.

As the CONSORT-EHEALTH checklist is still considered in a formative stage, we would ask that you also RATE ON A SCALE OF 1-5 how important/useful you feel each item is FOR THE PURPOSE OF THE CHECKLIST and reporting guideline (optional).

Mandatory reporting items are marked with a red *.
In the textboxes, either copy & paste the relevant sections from your manuscript into this form - please include any quotes from your manuscript in QUOTATION MARKS,
or answer directly by providing additional information not in the manuscript, or elaborating on why the item was not relevant for this study.

YOUR ANSWERS WILL BE PUBLISHED AS A SUPPLEMENTARY FILE TO YOUR PUBLICATION IN JMIR AND ARE CONSIDERED PART OF YOUR PUBLICATION (IF ACCEPTED).
Please fill in these questions diligently. Information will not be copyedited, so please use proper spelling and grammar, use correct capitalization, and avoid abbreviations.

DO NOT FORGET TO SAVE AS PDF _AND_ CLICK THE SUBMIT BUTTON SO YOUR ANSWERS ARE IN OUR DATABASE !!!

Citation Suggestion (if you append the pdf as Appendix we suggest to cite this paper in the caption):
Eysenbach G, CONSORT-EHEALTH Group
CONSORT-EHEALTH: Improving and Standardizing Evaluation Reports of Web-based and Mobile Health Interventions
J Med Internet Res 2011;13(4):e126
URL: http://www.jmir.org/2011/4/e126/
doi: 10.2196/jmir.1923
PMID: 22209829

* Required

Your name *
First Last

Justin Koufopoulos

Primary Affiliation (short), City, Country *
University of Toronto, Toronto, Canada

University of Leeds, Lee

Your e-mail address *
abc@gmail.com

jkoufopoulos@gmail.cor

Title of your manuscript *
Provide the (draft) title of your manuscript.

A web-based and mobile health social support intervention to promote adherence to inhaled asthma medications: randomized controlled trial

Article Preparation Status/Stage *
At which stage in your article preparation are you currently (at the time you fill in this form)

- ○ not submitted yet - in early draft status
- ● not submitted yet - in late draft status, just before submission
- ○ submitted to a journal but not reviewed yet
- ○ submitted to a journal and after receiving initial reviewer comments
- ○ submitted to a journal and accepted, but not published yet
- ○ published
- ○ Other:

Journal *
If you already know where you will submit this paper (or if it is already submitted), please provide the journal name (if it is not JMIR, provide the journal name under "other")

- ○ not submitted yet / unclear where I will submit this
- ● Journal of Medical Internet Research (JMIR)
- ○ Other:

Manuscript tracking number *
If this is a JMIR submission, please provide the manuscript tracking number under "other" (The ms tracking number can be found in the submission acknowledgement email, or when you login as author in JMIR. If the paper is already published in JMIR, then the ms tracking number is the four-digit number at the end of the DOI, to be found at the bottom of each published article in JMIR)

◉ no ms number (yet) / not (yet) submitted to / published in JMIR

○ Other:

TITLE AND ABSTRACT

1a) TITLE: Identification as a randomized trial in the title

1a) Does your paper address CONSORT item 1a? *

I.e does the title contain the phrase "Randomized Controlled Trial"? (if not, explain the reason under "other")

◉ yes

○ Other:

1a-i) Identify the mode of delivery in the title

Identify the mode of delivery. Preferably use “web-based” and/or “mobile” and/or “electronic game” in the title. Avoid ambiguous terms like “online”, “virtual”, “interactive”. Use “Internet-based” only if Intervention includes non-web-based Internet components (e.g. email), use “computer-based” or “electronic” only if offline products are used. Use “virtual” only in the context of “virtual reality” (3-D worlds). Use “online” only in the context of “online support groups”. Complement or substitute product names with broader terms for the class of products (such as “mobile” or “smart phone” instead of “iphone”), especially if the application runs on different platforms.

	1	2	3	4	5	
subitem not at all important	○	○	○	○	○	essential

Does your paper address subitem 1a-i? *

Copy and paste relevant sections from manuscript title (include quotes in quotation marks "like this" to indicate direct quotes from your manuscript), or elaborate on this item by providing additional information not in the ms, or briefly explain why the item is not applicable/relevant for your study

A web-based and mobile health social support intervention to promote adherence to inhaled asthma medications: randomized controlled trial

1a-ii) Non-web-based components or important co-interventions in title

Mention non-web-based components or important co-interventions in title, if any (e.g., “with telephone support”).

	1	2	3	4	5	
subitem not at all important	○	○	○	○	○	essential

Does your paper address subitem 1a-ii?

Copy and paste relevant sections from manuscript title (include quotes in quotation marks "like this" to indicate direct quotes from your manuscript), or elaborate on this item by providing additional information not in the ms, or briefly explain why the item is not applicable/relevant for your study

A web-based and mobile health social support intervention to promote adherence to inhaled asthma medications: randomized controlled trial

1a-iii) Primary condition or target group in the title

Mention primary condition or target group in the title, if any (e.g., “for children with Type I Diabetes”) Example: A Web-based and Mobile Intervention with Telephone Support for Children with Type I Diabetes: Randomized Controlled Trial

	1	2	3	4	5	
subitem not at all important	○	○	○	○	○	essential

Does your paper address subitem 1a-iii? *

Copy and paste relevant sections from manuscript title (include quotes in quotation marks "like this" to indicate direct quotes from your manuscript), or elaborate on this item by providing additional information not in the ms, or briefly explain why the item is not applicable/relevant for your study

A web-based and mobile health social support intervention to promote adherence to inhaled asthma medications: randomized controlled trial

1b) ABSTRACT: Structured summary of trial design, methods, results, and conclusions

NPT extension: Description of experimental treatment, comparator, care providers, centers, and blinding status.

1b-i) Key features/functionalities/components of the intervention and comparator in the METHODS section of the ABSTRACT

Mention key features/functionalities/components of the intervention and comparator in the abstract. If possible, also mention theories and principles used for designing the site. Keep in mind the needs of systematic reviewers and indexers by including important synonyms. (Note: Only report in the abstract what the main paper is reporting. If this information is missing from the main body of text, consider adding it)

	1	2	3	4	5	
subitem not at all important	○	○	○	○	○	essential

Does your paper address subitem 1b-i? *

Copy and paste relevant sections from the manuscript abstract (include quotes in quotation marks "like this" to indicate direct quotes from your manuscript), or elaborate on this item by providing additional information not in the ms, or briefly explain why the item is not applicable/relevant for your study

Background: Many studies of eHealth interventions lack rigorous assessment. Online communities hold great potential as interventions for health, particularly for the management of chronic illness. The social support that online communities can provide has been associated with positive treatment outcomes, including medication adherence. Few studies have attempted to assess whether membership of an online community improves health outcomes using rigorous designs.
Objective: Our objective was to conduct a rigorous proof-of-concept randomized controlled trial of an online community intervention for improving adherence to asthma medicine.
Methods: This nine-week randomized controlled trial included a sample of asthmatic adults from the United Kingdom prescribed an inhaled corticosteroid preventer. Participants were recruited via email and randomized to either an “online community”, or “no-online community” condition. After each instance of preventer use, participants (n = 216) were required to report the number of doses of medication taken in a short post. Those randomized to the online community condition (n=99) could read the posts of other community members, reply, and create their own posts. Participants randomized to the no-online community condition (n=117) also posted their medication use but could not read others’ posts. The main outcome measures were self-reported medication adherence taken at baseline and follow-up, and an objective measure of adherence to the intervention (visits to site). Results: 103 participants completed the study (37.8% in the intervention condition and 62.2% in the control condition). MANOVA of adherence to asthma preventer medicine at follow-up controlling for baseline use was not significantly different between conditions in either intention-to-treat or per protocol analysis (P = .99). Site use was generally higher in the control compared to intervention conditions.
Conclusions: Joining an online community did not improve adherence to preventer medication for asthma patients. Without greater community support beyond reporting medication use, the current findings do not support the use of an online community to improve adherence.
Trial Registration: ISRCTN trial registration number 29399269.

1b-ii) Level of human involvement in the METHODS section of the ABSTRACT

Clarify the level of human involvement in the abstract, e.g., use phrases like “fully automated” vs. “therapist/nurse/care provider/physician-assisted” (mention number and expertise of providers involved, if any). (Note: Only report in the abstract what the main paper is reporting. If this information is missing from the main body of text, consider adding it)

	1	2	3	4	5	
subitem not at all important	○	○	○	○	○	essential

Does your paper address subitem 1b-ii?

Copy and paste relevant sections from the manuscript abstract (include quotes in quotation marks "like this" to indicate direct quotes from your manuscript), or elaborate on this item by providing additional information not in the ms, or briefly explain why the item is not applicable/relevant for your study

Background: Many studies of eHealth interventions lack rigorous assessment. Online communities hold great potential as interventions for health, particularly for the management of chronic illness. The social support that online communities can provide has been associated with positive treatment outcomes, including medication adherence. Few studies have attempted to assess whether membership of an online community improves health outcomes using rigorous designs.
Objective: Our objective was to conduct a rigorous proof-of-concept randomized controlled trial of an online community intervention for improving adherence to asthma medicine.
Methods: This nine-week randomized controlled trial included a sample of asthmatic adults from the United Kingdom prescribed an inhaled corticosteroid preventer. Participants were recruited via email and randomized to either an “online community”, or “no-online community” condition. After each instance of preventer use, participants (n = 216) were required to report the number of doses of medication taken in a short post. Those randomized to the online community condition (n=99) could read the posts of other community members, reply, and create their own posts. Participants randomized to the no-online community condition (n=117) also posted their medication use but could not read others’ posts. The main outcome measures were self-reported medication adherence taken at baseline and follow-up, and an objective measure of adherence to the intervention (visits to site). Results: 103 participants completed the study (37.8% in the intervention condition and 62.2% in the control condition). MANOVA of adherence to asthma preventer medicine at follow-up controlling for baseline use was not significantly different between conditions in either intention-to-treat or per protocol analysis (P = .99). Site use was generally higher in the control compared to intervention conditions.
Conclusions: Joining an online community did not improve adherence to preventer medication for asthma patients. Without greater community support beyond reporting medication use, the current [illegible]

1b-iii) Open vs. closed, web-based (self-assessment) vs. face-to-face assessments in the METHODS section of the ABSTRACT

Mention how participants were recruited (online vs. offline), e.g., from an open access website or from a clinic or a closed online user group (closed usergroup trial), and clarify if this was a purely web-based trial, or there were face-to-face components (as part of the intervention or for assessment). Clearly say if outcomes were self-assessed through questionnaires (as common in web-based trials). Note: In traditional offline trials, an open trial (open-label trial) is a type of clinical trial in which both the researchers and participants know which treatment is being administered. To avoid confusion, use “blinded” or “unblinded” to indicated the level of blinding instead of “open”, as “open” in web-based trials usually refers to “open access” (i.e. participants can self-enrol). (Note: Only report in the abstract what the main paper is reporting. If this information is missing from the main body of text, consider adding it)

	1	2	3	4	5	
subitem not at all important	○	○	○	○	○	essential

Does your paper address subitem 1b-iii?

Copy and paste relevant sections from the manuscript abstract (include quotes in quotation marks "like

this" to indicate direct quotes from your manuscript), or elaborate on this item by providing additional information not in the ms, or briefly explain why the item is not applicable/relevant for your study

Background: Many studies of eHealth interventions lack rigorous assessment. Online communities hold great potential as interventions for health, particularly for the management of chronic illness. The social support that online communities can provide has been associated with positive treatment outcomes, including medication adherence. Few studies have attempted to assess whether membership of an online community improves health outcomes using rigorous designs.
Objective: Our objective was to conduct a rigorous proof-of-concept randomized controlled trial of an online community intervention for improving adherence to asthma medicine.
Methods: This nine-week randomized controlled trial included a sample of asthmatic adults from the United Kingdom prescribed an inhaled corticosteroid preventer. Participants were recruited via email and randomized to either an "online community", or "no-online community" condition. After each instance of preventer use, participants (n = 216) were required to report the number of doses of medication taken in a short post. Those randomized to the online community condition (n=99) could read the posts of other community members, reply, and create their own posts. Participants randomized to the no-online community condition (n=117) also posted their medication use but could not read others' posts. The main outcome measures were self-reported medication adherence taken at baseline and follow-up, and an objective measure of adherence to the intervention (visits to site). Results: 103 participants completed the study (37.8% in the intervention condition and 62.2% in the control condition). MANOVA of adherence to asthma preventer medicine at follow-up controlling for baseline use was not significantly different between conditions in either intention-to-treat or per protocol analysis (P = .99). Site use was generally higher in the control compared to intervention conditions.
Conclusions: Joining an online community did not improve adherence to preventer medication for asthma patients. Without greater community support beyond reporting medication use, the current findings do not support the use of an online community to improve [illegible]

1b-iv) RESULTS section in abstract must contain use data

Report number of participants enrolled/assessed in each group, the use/uptake of the intervention (e.g., attrition/adherence metrics, use over time, number of logins etc.), in addition to primary/secondary outcomes. (Note: Only report in the abstract what the main paper is reporting. If this information is missing from the main body of text, consider adding it)

	1	2	3	4	5	
subitem not at all important	○	○	○	○	○	essential

Does your paper address subitem 1b-iv?

Copy and paste relevant sections from the manuscript abstract (include quotes in quotation marks "like this" to indicate direct quotes from your manuscript), or elaborate on this item by providing additional information not in the ms, or briefly explain why the item is not applicable/relevant for your study

Background: Many studies of eHealth interventions lack rigorous assessment. Online communities hold great potential as interventions for health, particularly for the management of chronic illness. The social support that online communities can provide has been associated with positive treatment outcomes, including medication adherence. Few studies have attempted to assess whether membership of an online community improves health outcomes using rigorous designs.
Objective: Our objective was to conduct a rigorous proof-of-concept randomized controlled trial of an online community intervention for improving adherence to asthma medicine.
Methods: This nine-week randomized controlled trial included a sample of asthmatic adults from the United Kingdom prescribed an inhaled corticosteroid preventer. Participants were recruited via email and randomized to either an “online community”, or “no-online community” condition. After each instance of preventer use, participants (n = 216) were required to report the number of doses of medication taken in a short post. Those randomized to the online community condition (n=99) could read the posts of other community members, reply, and create their own posts. Participants randomized to the no-online community condition (n=117) also posted their medication use but could not read others’ posts. The main outcome measures were self-reported medication adherence taken at baseline and follow-up, and an objective measure of adherence to the intervention (visits to site). Results: 103 participants completed the study (37.8% in the intervention condition and 62.2% in the control condition). MANOVA of adherence to asthma preventer medicine at follow-up controlling for baseline use was not significantly different between conditions in either intention-to-treat or per protocol analysis (P = .99). Site use was generally higher in the control compared to intervention conditions.
Conclusions: Joining an online community did not improve adherence to preventer medication for asthma patients. Without greater community support beyond reporting medication use, the current findings do not support the use of an online community to improve adherence.
Trial Registration: ISRCTN trial registration number 29399269.

1b-v) CONCLUSIONS/DISCUSSION in abstract for negative trials

Conclusions/Discussions in abstract for negative trials: Discuss the primary outcome - if the trial is negative (primary outcome not changed), and the intervention was not used, discuss whether negative results are attributable to lack of uptake and discuss reasons. (Note: Only report in the abstract what the main paper is reporting. If this information is missing from the main body of text, consider adding it)

	1	2	3	4	5	
subitem not at all important	○	○	○	○	○	essential

Does your paper address subitem 1b-v?

Copy and paste relevant sections from the manuscript abstract (include quotes in quotation marks "like this" to indicate direct quotes from your manuscript), or elaborate on this item by providing additional information not in the ms, or briefly explain why the item is not applicable/relevant for your study

Background: Many studies of eHealth interventions lack rigorous assessment. Online communities hold great potential as interventions for health, particularly for the management of chronic illness. The social support that online communities can provide has been associated with positive treatment outcomes, including medication adherence. Few studies have attempted to assess whether membership of an online community improves health outcomes using rigorous designs.
Objective: Our objective was to conduct a rigorous proof-of-concept randomized controlled trial of an online community intervention for improving adherence to asthma medicine.
Methods: This nine-week randomized controlled trial included a sample of asthmatic adults from the United Kingdom prescribed an inhaled corticosteroid preventer. Participants were recruited via email and randomized to either an "online community", or "no-online community" condition. After each instance of preventer use, participants (n = 216) were required to report the number of doses of medication taken in a short post. Those randomized to the online community condition (n=99) could read the posts of other community members, reply, and create their own posts. Participants randomized to the no-online community condition (n=117) also posted their medication use but could not read others' posts. The main outcome measures were self-reported medication adherence taken at baseline and follow-up, and an objective measure of adherence to the intervention (visits to site). Results: 103 participants completed the study (37.8% in the intervention condition and 62.2% in the control condition). MANOVA of adherence to asthma preventer medicine at follow-up controlling for baseline use was not significantly different between conditions in either intention-to-treat or per protocol analysis (P = .99). Site use was generally higher in the control compared to intervention conditions.
Conclusions: Joining an online community did not improve adherence to preventer medication for asthma patients. Without greater community support beyond reporting medication use, the current findings do not support the use of an online community to improve adherence.
Trial Registration: ISRCTN trial registration number 29399269.

INTRODUCTION

2a) In INTRODUCTION: Scientific background and explanation of rationale

2a-i) Problem and the type of system/solution

Describe the problem and the type of system/solution that is object of the study: intended as stand-alone intervention vs. incorporated in broader health care program? Intended for a particular patient population? Goals of the intervention, e.g., being more cost-effective to other interventions, replace or complement other solutions? (Note: Details about the intervention are provided in "Methods" under 5)

	1	2	3	4	5	
subitem not at all important	○	○	○	○	○	essential

Does your paper address subitem 2a-i? *

Copy and paste relevant sections from the manuscript (include quotes in quotation marks "like this" to indicate direct quotes from your manuscript), or elaborate on this item by providing additional information not in the ms, or briefly explain why the item is not applicable/relevant for your study

Introduction
Electronic health or eHealth presents an exciting opportunity to create engaging interventions, that, because of the Internet and growing number of Internet-connected devices, can reach nearly anyone, almost anywhere[1]. Despite this opportunity, many studies of eHealth interventions lack rigorous assessment [2, 3]. Often, eHealth studies are observational, noncontrolled studies [3 - 5]. There is a need for greater use of randomized controlled trials in eHealth research so as to determine how intervention components contribute to the overall success or failure of an eHealth intervention [3 - 6].

Online communities are often used in eHealth interventions [7 - 13]. While there is no single accepted definition of an online community, a common definition is, "…a group of people who share a strong common interest, form relationships, and interact online" [14]. Online communities have the potential to foster feelings of social support in patients battling chronic health issues [14 -16]. The management of chronic health issues falls mostly on the patient and their family and can create a sense of isolation and distress [17]. PatientsLikeMe is a well-publicized example of an online community for patients. The website is one of the largest online groups of patients with amyotrophic lateral sclerosis. Members can share health data and communicate with one another using the online interface [18]. Such online communities can provide valuable support, but do they also relate to better management of chronic health issues such as increased medication adherence?
Link Between Social Support and Medication Adherence
Medication adherence can be defined as the extent to which a patient follows medication taking guidelines agreed upon by a patient and doctor [19]. Common guidelines include medication dosage and frequency. According to the World Health Organization (WHO), adherence to asthma medicine is just 50%, representing a significant health threat including increased risk of hospitalization and death [17].

Adherence to medication is influenced by social support. In a systematic review of 122 studies published between 1948 and 2001, DiMatteo [20] found a significant relationship between social support and adherence. Studies were categorized into types of support, including practical support (eg, instrumental support, assistance, reminders, organization, support for a specific behavior), emotional support, unidimensional social support (involving multiple types of social support, not separated in their measurement), family cohesiveness (eg, warmth, closeness, acceptance), and marital status and living arrangement. Patients receiving practical support were 3.6 times more likely to adhere to treatment regimens than those who were not. Risk of nonadherence was also found to be 1.53 times more likely if patients had low social support. Equivalence between online and face-to-face interventions in related fields suggests that online support could similarly provide social support and be associated with improved medication adherence [21].
Current State of the Evidence for Online Communities
There have been only a few randomized controlled trials of online communities for patients with chronic health issues [7, 9 - 13]. The results of these studies have been mixed. For example, one randomized controlled trial by Imanaka et al [7], attempted to evaluate the effect of peer-to-peer support via shared health statuses

evaluate the effect of peer-to-peer support via shared health statuses for obese-patient weight-loss. Participants were randomized to either a web-based system, where they could receive counseling from a dietician and compare their own changes in weight and lifestyle with other patients through health status updates (an online community), or an email-based system, in which participants could receive email counseling, but not interact or learn about the other patients in their condition. Compared to the email condition, weight loss in the web-based condition was significantly greater, although changes in body mass index, quality of life scores, general health perception, and mental health did not differ.

Similarly, a randomized controlled trial by Richardson et al [9], found that while an online community for an Internet-mediated walking program did not increase participant step count, participants randomized to the online community had greater engagement, and lower rates of attrition than the control group. Research has also shown that participation in online communities may even be harmful under certain conditions. Takahashi et al [11] found that users who interacted with other users who were moderately to severely depressed or had negative perception of the online community became even more depressed.

We found two trials that used Facebook to evaluate the effectiveness of online communities on physical activity and weight loss [12, 13]. In a RCT to evaluate the feasibility and efficacy of a Facebook-based intervention on physical activity for young adult cancer survivors, participants were randomized to either a Facebook online community intervention condition (FITNET), or a Facebook self-care (SC) condition. Increases in light physical activity were more than 2 hours per week greater in the FITNET condition compared to the SC condition [12]. In another trial using Facebook, students with a body mass index of 25-50 kg/m2 were randomized to Facebook, a Facebook, text messaging and personal feedback group, and a wait list. After 8 weeks, the Facebook, text messaging, and personal feedback group had significantly greater weight loss than either of the other two groups [13].

Theoretical framework

We predicted that participating in an online community would lead to greater medication adherence. The theoretical underpinnings of this prediction are Social Cognitive Theory [22, 23] and the stress and coping perspective of social support [24]. The effect of community website exposure [10] is also included in the theoretical framework of the intervention. According to Social Cognitive Theory [22, 23], individuals can learn by observing the actions of others. If those actions produce an effect that is beneficial to the individual being observed, those actions are more likely to be imitated. It was predicted that participants observing the adherence of other patients will themselves improve adherence to inhaled corticosteroid (ICS) treatment. Participants reading other patients' success stories regarding adherence or dealing with asthma more generally will learn from these stories and apply these lessons to their own life, improving adherence.

Social support has been defined as the quality and structure of an individual's relationships, and is associated with improvements in adherence to medication regimes [20]. As is predicted in the stress-buffering perspective of social support [24], the perception of having socially supportive relationships and the support that participants actually receive will reduce stress associated with adherence and asthma improving adherence and overall health.

Objective and Hypothesis

Our objective was to conduct a rigorous study of the effects of participating in an online community on adherence to asthma medicine. To our knowledge there are no previous studies of the relationship between online communities and medication adherence. Asthma was chosen as the target illness for this trial because medication adherence is often low and the incidence of chronic asthma in adults is high: nearly 10% in the United Kingdom [25].

2a-ii) Scientific background, rationale: What is known about the (type of) system

Scientific background, rationale: What is known about the (type of) system that is the object of the study (be sure to discuss the use of similar systems for other conditions/diagnoses, if appropiate), motivation for the study, i.e. what are the reasons for and what is the context for this specific study, from which stakeholder viewpoint is the study performed, potential impact of findings [2]. Briefly justify the choice of the comparator.

	1	2	3	4	5	
subitem not at all important	○	○	○	○	○	essential

Does your paper address subitem 2a-ii? *

Copy and paste relevant sections from the manuscript (include quotes in quotation marks "like this" to indicate direct quotes from your manuscript), or elaborate on this item by providing additional information not in the ms, or briefly explain why the item is not applicable/relevant for your study

Introduction
Electronic health or eHealth presents an exciting opportunity to create engaging interventions, that, because of the Internet and growing number of Internet-connected devices, can reach nearly anyone, almost anywhere[1]. Despite this opportunity, many studies of eHealth interventions lack rigorous assessment [2, 3]. Often, eHealth studies are observational, noncontrolled studies [3 - 5]. There is a need for greater use of randomized controlled trials in eHealth research so as to determine how intervention components contribute to the overall success or failure of an eHealth intervention [3 - 6].

Online communities are often used in eHealth interventions [7 - 13]. While there is no single accepted definition of an online community, a common definition is, "…a group of people who share a strong common interest, form relationships, and interact online" [14]. Online communities have the potential to foster feelings of social support in patients battling chronic health issues [14 -16]. The management of chronic health issues falls mostly on the patient and their family and can create a sense of isolation and distress [17]. PatientsLikeMe is a well-publicized example of an online community for patients. The website is one of the largest online groups of patients with amyotrophic lateral sclerosis. Members can share health data and communicate with one another using the online interface [18]. Such online communities can provide valuable support, but do they also relate to better management of chronic health issues such as increased medication adherence?
Link Between Social Support and Medication Adherence
Medication adherence can be defined as the extent to which a patient follows medication taking guidelines agreed upon by a patient and doctor [19]. Common guidelines include medication dosage and frequency. According to the World Health Organization (WHO), adherence to asthma medicine is just 50%, representing a significant health threat including increased risk of hospitalization and death [17].

Adherence to medication is influenced by social support. In a systematic review of 122 studies published between 1948 and 2001, DiMatteo [20] found a significant relationship between social support and adherence. Studies were categorized into types of support, including practical support (eg, instrumental support, assistance, reminders, organization, support for a specific behavior), emotional support, unidimensional social support (involving multiple types of social support, not separated in their measurement), family cohesiveness (eg, warmth, closeness, acceptance), and marital status and living arrangement. Patients receiving practical support were 3.6 times more likely to adhere to treatment regimens than those who were not. Risk of nonadherence was also found to be 1.53 times more likely if patients had low social support. Equivalence between online and face-to-face interventions in related fields suggests that online support could similarly provide social support and be associated with improved medication adherence [21].

Current State of the Evidence for Online Communities

There have been only a few randomized controlled trials of online communities for patients with chronic health issues [7, 9 - 13]. The results of these studies have been mixed. For example, one randomized controlled trial by Imanaka et al [7], attempted to evaluate the effect of peer-to-peer support via shared health statuses for obese-patient weight-loss. Participants were randomized to either a web-based system, where they could receive counseling from a dietician and compare their own changes in weight and lifestyle with other patients through health status updates (an online community), or an email-based system, in which participants could receive email counseling, but not interact or learn about the other patients in their condition. Compared to the email condition, weight loss in the web-based condition was significantly greater, although changes in body mass index, quality of life scores, general health perception, and mental health did not differ.

Similarly, a randomized controlled trial by Richardson et al [9], found that while an online community for an Internet-mediated walking program did not increase participant step count, participants randomized to the online community had greater engagement, and lower rates of attrition than the control group. Research has also shown that participation in online communities may even be harmful under certain conditions. Takahashi et al [11] found that users who interacted with other users who were moderately to severely depressed or had negative perception of the online community became even more depressed.

We found two trials that used Facebook to evaluate the effectiveness of online communities on physical activity and weight loss [12, 13]. In a RCT to evaluate the feasibility and efficacy of a Facebook-based intervention on physical activity for young adult cancer survivors, participants were randomized to either a Facebook online community intervention condition (FITNET), or a Facebook self-care (SC) condition. Increases in light physical activity were more than 2 hours per week greater in the FITNET condition compared to the SC condition [12]. In another trial using Facebook, students with a body mass index of 25-50 kg/m2 were randomized to Facebook, a Facebook, text messaging and personal feedback group, and a wait list. After 8 weeks, the Facebook, text messaging, and personal feedback group had significantly greater weight loss than either of the other two groups [13].

Theoretical framework

We predicted that participating in an online community would lead to greater medication adherence. The theoretical underpinnings of this

prediction are Social Cognitive Theory [22, 23] and the stress and coping perspective of social support [24]. The effect of community website exposure [10] is also included in the theoretical framework of the intervention. According to Social Cognitive Theory [22, 23], individuals can learn by observing the actions of others. If those actions produce an effect that is beneficial to the individual being observed, those actions are more likely to be imitated. It was predicted that participants observing the adherence of other patients will themselves improve adherence to inhaled corticosteroid (ICS) treatment. Participants reading other patients' success stories regarding adherence or dealing with asthma more generally will learn from these stories and apply these lessons to their own life, improving adherence.

Social support has been defined as the quality and structure of an individual's relationships, and is associated with improvements in adherence to medication regimes [20]. As is predicted in the stress-buffering perspective of social support [24], the perception of having socially supportive relationships and the support that participants actually receive will reduce stress associated with adherence and asthma improving adherence and overall health.
Objective and Hypothesis
Our objective was to conduct a rigorous study of the effects of participating in an online community on adherence to asthma medicine. To our knowledge there are no previous studies of the relationship between online communities and medication adherence. Asthma was chosen as the target illness for this trial because medication adherence is often low and the incidence of chronic asthma in adults is high: nearly 10% in the United Kingdom [25]. Asthma in adults is typically treated with a combination of an ICS preventer and a bronchodilator reliever. We hypothesized that adherence would be improved by participation in an online

2b) In INTRODUCTION: Specific objectives or hypotheses

Does your paper address CONSORT subitem 2b? *

Copy and paste relevant sections from the manuscript (include quotes in quotation marks "like this" to indicate direct quotes from your manuscript), or elaborate on this item by providing additional information not in the ms, or briefly explain why the item is not applicable/relevant for your study

Objective and Hypothesis
Our objective was to conduct a rigorous study of the effects of participating in an online community on adherence to asthma medicine. To our knowledge there are no previous studies of the relationship between online communities and medication adherence. Asthma was chosen as the target illness for this trial because medication adherence is often low and the incidence of chronic asthma in adults is high: nearly 10% in the United Kingdom [25]. Asthma in adults is typically treated with a combination of an ICS preventer and a bronchodilator reliever. We hypothesized that adherence would be improved by participation in an online community due to processes of modeling and social support.

METHODS

3a) Description of trial design (such as parallel, factorial) including allocation ratio

Does your paper address CONSORT subitem 3a? *

Copy and paste relevant sections from the manuscript (include quotes in quotation marks "like this" to indicate direct quotes from your manuscript), or elaborate on this item by providing additional information not in the ms, or briefly explain why the item is not applicable/relevant for your study

Study Design
In this two-arm randomized controlled trial participants were enrolled into either the intervention condition: “AsthmaVillage”, an online community for patients with asthma, or the control condition: “AsthmaDiary”, an online diary for recording ICS preventer use. Intervention arm participants had access to an online community, and could leave comments or see who else was online. In contrast, the control arm participants could not read the posts of other control-arm participants, or interact with other participants online. An active control was used to test clearly the effect of the community on adherence, and prevent participants from guessing if they were in the group of interest. The study was carried out for nine weeks, between June 24th, 2013 and August 26th, 2013. The trial conformed to the Consolidated Standards of Reporting Trials (CONSORT)-eHealth Checklist (Multimedia Appendix 1) [34].

3b) Important changes to methods after trial commencement (such as eligibility criteria), with

reasons

Does your paper address CONSORT subitem 3b? *

Copy and paste relevant sections from the manuscript (include quotes in quotation marks "like this" to indicate direct quotes from your manuscript), or elaborate on this item by providing additional information not in the ms, or briefly explain why the item is not applicable/relevant for your study

N/A

3b-i) Bug fixes, Downtimes, Content Changes

Bug fixes, Downtimes, Content Changes: ehealth systems are often dynamic systems. A description of changes to methods therefore also includes important changes made on the intervention or comparator during the trial (e.g., major bug fixes or changes in the functionality or content) (5-iii) and other "unexpected events" that may have influenced study design such as staff changes, system failures/downtimes, etc. [2].

	1	2	3	4	5	
subitem not at all important	○	○	○	○	○	essential

Does your paper address subitem 3b-i?

Copy and paste relevant sections from the manuscript (include quotes in quotation marks "like this" to indicate direct quotes from your manuscript), or elaborate on this item by providing additional information not in the ms, or briefly explain why the item is not applicable/relevant for your study

N/A

4a) Eligibility criteria for participants

Does your paper address CONSORT subitem 4a? *

Copy and paste relevant sections from the manuscript (include quotes in quotation marks "like this" to indicate direct quotes from your manuscript), or elaborate on this item by providing additional information not in the ms, or briefly explain why the item is not applicable/relevant for your study

Recruitment
A total of 1,833 emails requesting participants for a study on asthma management were sent out to department secretaries of the 40 largest universities in the UK by enrollment. Recruitment emails were sent over a period of 10 days from June 13th, 2013 through June 23rd, 2013. Department secretaries were asked in the body of the email to forward the request to department mailing lists (Multimedia Appendix 2). The request for participants invited individuals managing their asthma with an ICS preventer to fill out an eligibility screening form and included a link to the questionnaire (Multimedia Appendix 3). Participants were also informed that upon successful completion of the study they would receive a £20 (approximately $30) shopping voucher. Successful completion of the study was defined as recording their ICS preventer use at least once per week on the site.
Eligibility Screening and Consent
Nine hundred and thirty-six participants responded to the eligibility questionnaire. Participants were excluded from the study if they failed to complete the eligibility questionnaire (n = 256) or baseline measures (n = 221), did not have asthma (n = 105), were not prescribed an ICS preventer inhaler (n = 87), or had previously taken the pilot (n = 9). After screening, a total of 251 participants were eligible for study inclusion. See Figure 1 for details.

4a-i) Computer / Internet literacy

Computer / Internet literacy is often an implicit “de facto” eligibility criterion - this should be explicitly clarified.

	1	2	3	4	5	
subitem not at all important	○	○	○	○	○	essential

Does your paper address subitem 4a-i?

Copy and paste relevant sections from the manuscript (include quotes in quotation marks "like this" to indicate direct quotes from your manuscript), or elaborate on this item by providing additional information not in the ms, or briefly explain why the item is not applicable/relevant for your study

Recruitment
A total of 1,833 emails requesting participants for a study on asthma management were sent out to department secretaries of the 40 largest universities in the UK by enrollment. Recruitment emails were sent over a period of 10 days from June 13th, 2013 through June 23rd, 2013. Department secretaries were asked in the body of the email to forward the request to department mailing lists (Multimedia Appendix 2). The request for participants invited individuals managing their asthma with an ICS preventer to fill out an eligibility screening form and included a link to the questionnaire (Multimedia Appendix 3). Participants were also informed that upon successful completion of the study they would receive a £20 (approximately $30) shopping voucher. Successful completion of the study was defined as recording their ICS preventer use at least once per week on the site.
Eligibility Screening and Consent
Nine hundred and thirty-six participants responded to the eligibility questionnaire. Participants were excluded from the study if they failed to complete the eligibility questionnaire (n = 256) or baseline measures (n = 221), did not have asthma (n = 105), were not prescribed an ICS preventer inhaler (n = 87), or had previously taken the pilot (n = 9). After screening, a total of 251 participants were eligible for study inclusion. See Figure 1 for details.

4a-ii) Open vs. closed, web-based vs. face-to-face assessments:

Open vs. closed, web-based vs. face-to-face assessments: Mention how participants were recruited (online vs. offline), e.g., from an open access website or from a clinic, and clarify if this was a purely web-based trial, or there were face-to-face components (as part of the intervention or for assessment), i.e., to what degree got the study team to know the participant. In online-only trials, clarify if participants were quasi-anonymous and whether having multiple identities was possible or whether technical or logistical measures (e.g., cookies, email confirmation, phone calls) were used to detect/prevent these.

	1	2	3	4	5	
subitem not at all important	○	○	○	○	○	essential

Does your paper address subitem 4a-ii? *

Copy and paste relevant sections from the manuscript (include quotes in quotation marks "like this" to indicate direct quotes from your manuscript), or elaborate on this item by providing additional information not in the ms, or briefly explain why the item is not applicable/relevant for your study

Recruitment
A total of 1,833 emails requesting participants for a study on asthma management were sent out to department secretaries of the 40 largest universities in the UK by enrollment. Recruitment emails were sent over a period of 10 days from June 13th, 2013 through June 23rd, 2013. Department secretaries were asked in the body of the email to forward the request to department mailing lists (Multimedia Appendix 2). The request for participants invited individuals managing their asthma with an ICS preventer to fill out an eligibility screening form and included a link to the questionnaire (Multimedia Appendix 3). Participants were also informed that upon successful completion of the study they would receive a £20 (approximately $30) shopping voucher. Successful completion of the study was defined as recording their ICS preventer use at least once per week on the site.
Eligibility Screening and Consent
Nine hundred and thirty-six participants responded to the eligibility questionnaire. Participants were excluded from the study if they failed to complete the eligibility questionnaire (n = 256) or baseline measures (n = 221), did not have asthma (n = 105), were not prescribed an ICS preventer inhaler (n = 87), or had previously taken the pilot (n = 9). After screening, a total of 251 participants were eligible for study inclusion. See Figure 1 for details.

Figure 1. Participant flow chart of recruitment, participation after exclusion criteria, randomization, and attrition.

Participants were automatically taken to the information sheet (Multimedia Appendix 4) and were asked to provide informed consent (Multimedia Appendix 5). Thirty-five participants refused to provide consent and were eliminated from the study, leaving 216 eligible participants. Participants were then randomized to the diary (n = 117) and online community (n = 99) conditions. Randomization occurred through a random number generator [26], yielding two unequal groups. The online screening survey was administered through Qualtrics [27], a subscription-based online survey software suite.

4a-iii) Information giving during recruitment

Information given during recruitment. Specify how participants were briefed for recruitment and in the informed consent procedures (e.g., publish the informed consent documentation as appendix, see also item X26), as this information may have an effect on user self-selection, user expectation and may also bias results.

	1	2	3	4	5	
subitem not at all important	○	○	○	○	○	essential

Does your paper address subitem 4a-iii?

Copy and paste relevant sections from the manuscript (include quotes in quotation marks "like this" to indicate direct quotes from your manuscript), or elaborate on this item by providing additional information not in the ms, or briefly explain why the item is not applicable/relevant for your study

Recruitment
A total of 1,833 emails requesting participants for a study on asthma management were sent out to department secretaries of the 40 largest universities in the UK by enrollment. Recruitment emails were sent over a period of 10 days from June 13th, 2013 through June 23rd, 2013. Department secretaries were asked in the body of the email to forward the request to department mailing lists (Multimedia Appendix 2). The request for participants invited individuals managing their asthma with an ICS preventer to fill out an eligibility screening form and included a link to the questionnaire (Multimedia Appendix 3). Participants were also informed that upon successful completion of the study they would receive a £20 (approximately $30) shopping voucher. Successful completion of the study was defined as recording their ICS preventer use at least once per week on the site.
Eligibility Screening and Consent
Nine hundred and thirty-six participants responded to the eligibility questionnaire. Participants were excluded from the study if they failed to complete the eligibility questionnaire (n = 256) or baseline measures (n = 221), did not have asthma (n = 105), were not prescribed an ICS preventer inhaler (n = 87), or had previously taken the pilot (n = 9). After screening, a total of 251 participants were eligible for study inclusion. See Figure 1 for details.

Figure 1. Participant flow chart of recruitment, participation after exclusion criteria, randomization, and attrition.

Participants were automatically taken to the information sheet (Multimedia Appendix 4) and were asked to provide informed consent (Multimedia Appendix 5). Thirty-five participants refused to provide consent and were eliminated from the study, leaving 216 eligible participants. Participants were then randomized to the diary (n = 117) and online community (n = 99) conditions. Randomization occurred through a random number generator [26], yielding two unequal groups. The online screening survey was administered through Qualtrics [27], a subscription-based online survey software suite.

4b) Settings and locations where the data were collected

Does your paper address CONSORT subitem 4b? *

Copy and paste relevant sections from the manuscript (include quotes in quotation marks "like this" to indicate direct quotes from your manuscript), or elaborate on this item by providing additional information not in the ms, or briefly explain why the item is not applicable/relevant for your study

Recruitment
A total of 1,833 emails requesting participants for a study on asthma management were sent out to department secretaries of the 40 largest universities in the UK by enrollment. Recruitment emails were sent over a period of 10 days from June 13th, 2013 through June 23rd, 2013. Department secretaries were asked in the body of the email to forward the request to department mailing lists (Multimedia Appendix 2). The request for participants invited individuals managing their asthma with an ICS preventer to fill out an eligibility screening form and included a link to the questionnaire (Multimedia Appendix 3). Participants were also informed that upon successful completion of the study they would receive a £20 (approximately $30) shopping voucher. Successful completion of the study was defined as recording their ICS preventer use at least once per week on the site.
Eligibility Screening and Consent
Nine hundred and thirty-six participants responded to the eligibility questionnaire. Participants were excluded from the study if they failed to complete the eligibility questionnaire (n = 256) or baseline measures (n = 221), did not have asthma (n = 105), were not prescribed an ICS preventer inhaler (n = 87), or had previously taken the pilot (n = 9). After screening, a total of 251 participants were eligible for study inclusion. See Figure 1 for details.

Participants were automatically taken to the information sheet (Multimedia Appendix 4) and were asked to provide informed consent (Multimedia Appendix 5). Thirty-five participants refused to provide consent and were eliminated from the study, leaving 216 eligible participants. Participants were then randomized to the diary (n = 117) and online community (n = 99) conditions. Randomization occurred through a random number generator [26], yielding two unequal groups. The online screening survey was administered through Qualtrics [27], a subscription-based online survey software suite.

4b-i) Report if outcomes were (self-)assessed through online questionnaires

Clearly report if outcomes were (self-)assessed through online questionnaires (as common in web-based trials) or otherwise.

	1	2	3	4	5	
subitem not at all important	○	○	○	○	○	essential

Does your paper address subitem 4b-i? *

Copy and paste relevant sections from the manuscript (include quotes in quotation marks "like this" to indicate direct quotes from your manuscript), or elaborate on this item by providing additional information not in the ms, or briefly explain why the item is not applicable/relevant for your study

Baseline Measures
Baseline measures were included as part of the eligibility screening. Participants completed an online survey (Multimedia Appendix 6) that included questions about gender, age, previous social networking use, and prescriptions.

Preventer adherence in both conditions was self-reported using the six-item Simplified Medication Adherence Questionnaire (SMAQ; [28], Multimedia Appendix 7), a common, validated measure of medication adherence. In the questionnaire, the SMAQ refers generally to all medicine. For example, the first item of the SMAQ reads, “Do you ever forget to take your medicine?” For this study all instances of the word, “medicine” was changed to “asthma preventer medication”. This small change was was unlikely to have affected the measure.

The SMAQ was then recalculated where all variables became dichotomous by rescoring item 4 of the SMAQ (“Thinking about the last week, how often have you not taken your asthma preventer medicine as prescribed?”) where more than two missed uses was equal to nonadherent. The rescored SMAQ formed a reliable measure (Cronbach’s alpha = .72), and all items were averaged at baseline. Missing values were given to any participant with fewer than two filled out SMAQ items.

4b-ii) Report how institutional affiliations are displayed

Report how institutional affiliations are displayed to potential participants [on ehealth media], as affiliations with prestigious hospitals or universities may affect volunteer rates, use, and reactions with regards to an intervention.(Not a required item – describe only if this may bias results)

	1	2	3	4	5	
subitem not at all important	○	○	○	○	○	essential

Does your paper address subitem 4b-ii?

Copy and paste relevant sections from the manuscript (include quotes in quotation marks "like this" to indicate direct quotes from your manuscript), or elaborate on this item by providing additional information not in the ms, or briefly explain why the item is not applicable/relevant for your study

Recruitment

A total of 1,833 emails requesting participants for a study on asthma management were sent out to department secretaries of the 40 largest universities in the UK by enrollment. Recruitment emails were sent over a period of 10 days from June 13th, 2013 through June 23rd, 2013. Department secretaries were asked in the body of the email to forward the request to department mailing lists (Multimedia Appendix 2). The request for participants invited individuals managing their asthma with an ICS preventer to fill out an eligibility screening form and included a link to the questionnaire (Multimedia Appendix 3). Participants were also informed that upon successful completion of the study they would receive a £20 (approximately $30) shopping voucher. Successful completion of the study was defined as recording their ICS preventer use at least once per week on the site.

Eligibility Screening and Consent

Nine hundred and thirty-six participants responded to the eligibility questionnaire. Participants were excluded from the study if they failed to complete the eligibility questionnaire (n = 256) or baseline measures (n = 221), did not have asthma (n = 105), were not prescribed an ICS preventer inhaler (n = 87), or had previously taken the pilot (n = 9). After screening, a total of 251 participants were eligible for study inclusion. See Figure 1 for details.

Participants were automatically taken to the information sheet (Multimedia Appendix 4) and were asked to provide informed consent (Multimedia Appendix 5). Thirty-five participants refused to provide consent and were eliminated from the study, leaving 216 eligible participants. Participants were then randomized to the diary (n = 117) and online community (n = 99) conditions. Randomization occurred through a random number generator [26], yielding two unequal groups. The online screening survey was administered through Qualtrics [27], a subscription-based online survey software suite.

Baseline Measures

Baseline measures were included as part of the eligibility screening. Participants completed an online survey (Multimedia Appendix 6) that included questions about gender, age, previous social networking use, and prescriptions.

Preventer adherence in both conditions was self-reported using the six-item Simplified Medication Adherence Questionnaire (SMAQ; [28], Multimedia Appendix 7), a common, validated measure of medication adherence. In the questionnaire, the SMAQ refers generally to all medicine. For example, the first item of the SMAQ reads, “Do you ever forget to take your medicine?” For this study all instances of the word, “medicine” was changed to “asthma preventer medication”. This small change was was unlikely to have affected the measure.

The SMAQ was then recalculated where all variables became dichotomous by rescoring item 4 of the SMAQ (“Thinking about the last week, how often have you not taken your asthma preventer medicine as prescribed?”) where more than two missed uses was equal to nonadherent. The rescored SMAQ formed a reliable measure (Cronbach’s alpha = .72), and all items were averaged at baseline. Missing values were given to any participant with fewer than two filled out SMAQ items.

5) The interventions for each group with sufficient details to allow replication, including how and when they were actually administered

5-i) Mention names, credential, affiliations of the developers, sponsors, and owners

Mention names, credential, affiliations of the developers, sponsors, and owners [6] (if authors/evaluators are owners or developer of the software, this needs to be declared in a "Conflict of interest" section or mentioned elsewhere in the manuscript).

	1	2	3	4	5	
subitem not at all important	○	○	○	○	○	essential

Does your paper address subitem 5-i?

Copy and paste relevant sections from the manuscript (include quotes in quotation marks "like this" to indicate direct quotes from your manuscript), or elaborate on this item by providing additional information not in the ms, or briefly explain why the item is not applicable/relevant for your study

Intervention
The online community was creating using Wordpress [29], an open-source content management system, and Buddypress [30] social networking features (Multimedia Appendix 8). There were four primary intervention components: (a) a homepage that displayed all site activity in a rolling status board, (b) a group diary for posting preventer use, (c) a page for posting questions and answers about asthma, and (d) a profile page.

The main actions participants could take on the online community were posting or creating comments. The intervention was developed to be accessible by smartphone web browsers, as well as by desktop versions.

The effect of membership in the online community is dependent on the extent members are using the website of the online community itself. Site engagement was a barrier for participants in the pilot. In order to create a website which closely resembled popular patient communities such as PatientsLikeMe, we created a site discussion section dedicated to posting questions regarding asthma. Such features have been shown to be beneficial to engagement in previous studies [9].
Control
The control condition comprised an online diary, AsthmaDiary. The online diary was created using Google Forms. A single-item survey was created (Multimedia Appendix 9). The item asked, "How many times did you take your preventer?" Participants randomized to the control condition could then input the number of puffs, and after entering their unique personal identification number (PIN), hit "submit." Since participants did not need to log in with a username to fill out the form, participants used a PIN that allowed their posts to be identified by the researcher. Participants in the control condition could not see the posts of the other participants, or otherwise know that

5-ii) Describe the history/development process

Describe the history/development process of the application and previous formative evaluations (e.g., focus groups, usability testing), as these will have an impact on adoption/use rates and help with

interpreting results.

	1	2	3	4	5	
subitem not at all important	○	○	○	○	○	essential

Does your paper address subitem 5-ii?

Copy and paste relevant sections from the manuscript (include quotes in quotation marks "like this" to indicate direct quotes from your manuscript), or elaborate on this item by providing additional information not in the ms, or briefly explain why the item is not applicable/relevant for your study

Pilot Study
Previous to this trial, a small pilot study (n = 8) was conducted to gather qualitative feedback on the usability of the online community. The study ran for 31 days. Participants were required to post their preventer use weekly in the online community. At the end of the study, participants were sent a questionnaire with short-answer items regarding the usefulness and usability of the site. Participants reported that they found the social support features of the intervention useful in connecting with other asthma patients, but found that after the first few weeks it was difficult to become engaged with the site as the number of active conversations diminished.

The results of the pilot study influenced the development of the final intervention administered during the randomized controlled trial. While the intervention remained largely unchanged, a separate page was created as part of the site for posting questions and answers about asthma.
Intervention
The online community was creating using Wordpress [29], an open-source content management system, and Buddypress [30] social networking features (Multimedia Appendix 8). There were four primary intervention components: (a) a homepage that displayed all site activity in a rolling status board, (b) a group diary for posting preventer use, (c) a page for posting questions and answers about asthma, and (d) a profile page.

The main actions participants could take on the online community were posting or creating comments. The intervention was developed to be accessible by smartphone web browsers, as well as by desktop versions.

The effect of membership in the online community is dependent on the extent members are using the website of the online community itself. Site engagement was a barrier for participants in the pilot. In order to create a website which closely resembled popular patient communities such as PatientsLikeMe, we created a site discussion section dedicated to posting questions regarding asthma. Such features have been shown to be beneficial to engagement in previous studies [9].
Control
The control condition comprised an online diary, AsthmaDiary. The online diary was created using Google Forms. A single-item survey was created (Multimedia Appendix 9). The item asked, "How many times did you take your preventer?" Participants randomized to the control condition could then input the number of puffs, and after entering their unique personal identification number (PIN), hit "submit." Since participants did not need to log in with a username to fill out the form, participants used a PIN that allowed their posts to be identified by the researcher. Participants in the control condition could not see the posts of the other participants, or otherwise know that there were other participants posting in their condition of the experiment

5-iii) Revisions and updating

Revisions and updating. Clearly mention the date and/or version number of the application/intervention (and comparator, if applicable) evaluated, or describe whether the intervention underwent major changes during the evaluation process, or whether the development and/or content was "frozen" during the trial. Describe dynamic components such as news feeds or changing content which may have an impact on the replicability of the intervention (for unexpected events see item 3b).

	1	2	3	4	5	
subitem not at all important	◯	◯	◯	◯	◯	essential

Does your paper address subitem 5-iii?

Copy and paste relevant sections from the manuscript (include quotes in quotation marks "like this" to indicate direct quotes from your manuscript), or elaborate on this item by providing additional information not in the ms, or briefly explain why the item is not applicable/relevant for your study

N/A

5-iv) Quality assurance methods

Provide information on quality assurance methods to ensure accuracy and quality of information provided [1], if applicable.

	1	2	3	4	5	
subitem not at all important	◯	◯	◯	◯	◯	essential

Does your paper address subitem 5-iv?

Copy and paste relevant sections from the manuscript (include quotes in quotation marks "like this" to indicate direct quotes from your manuscript), or elaborate on this item by providing additional information not in the ms, or briefly explain why the item is not applicable/relevant for your study

N/A

5-v) Ensure replicability by publishing the source code, and/or providing screenshots/screen-capture video, and/or providing flowcharts of the algorithms used

Ensure replicability by publishing the source code, and/or providing screenshots/screen-capture video, and/or providing flowcharts of the algorithms used. Replicability (i.e., other researchers should in principle be able to replicate the study) is a hallmark of scientific reporting.

	1	2	3	4	5	
subitem not at all important	◯	◯	◯	◯	◯	essential

Does your paper address subitem 5-v?

Copy and paste relevant sections from the manuscript (include quotes in quotation marks "like this" to

indicate direct quotes from your manuscript), or elaborate on this item by providing additional information not in the ms, or briefly explain why the item is not applicable/relevant for your study

Yes; in Figures and Multimedia Appendices.

5-vi) Digital preservation

Digital preservation: Provide the URL of the application, but as the intervention is likely to change or disappear over the course of the years; also make sure the intervention is archived (Internet Archive, webcitation.org, and/or publishing the source code or screenshots/videos alongside the article). As pages behind login screens cannot be archived, consider creating demo pages which are accessible without login.

	1	2	3	4	5	
subitem not at all important	○	○	○	○	○	essential

Does your paper address subitem 5-vi?

Copy and paste relevant sections from the manuscript (include quotes in quotation marks "like this" to indicate direct quotes from your manuscript), or elaborate on this item by providing additional information not in the ms, or briefly explain why the item is not applicable/relevant for your study

N/A.

5-vii) Access

Access: Describe how participants accessed the application, in what setting/context, if they had to pay (or were paid) or not, whether they had to be a member of specific group. If known, describe how participants obtained “access to the platform and Internet” [1]. To ensure access for editors/reviewers/readers, consider to provide a “backdoor” login account or demo mode for reviewers/readers to explore the application (also important for archiving purposes, see vi).

	1	2	3	4	5	
subitem not at all important	○	○	○	○	○	essential

Does your paper address subitem 5-vii? *

Copy and paste relevant sections from the manuscript (include quotes in quotation marks "like this" to indicate direct quotes from your manuscript), or elaborate on this item by providing additional information not in the ms, or briefly explain why the item is not applicable/relevant for your study

Procedure
After randomization, participants were emailed instructions on how to use their site (Multimedia Appendix 10, 11). Participants were then prompted to log into their respective websites. Before logging in, participants created a username and password for their sites. Participants randomized to the diary created a PIN which they were required to input whenever they posted preventer use. Participants in both conditions were quasi-anonymous. Since registration required an email confirmation, it is unlikely that a single individual operated multiple accounts.

During the trial, a weekly email was automated to send to participants indicating which week the trial was on, with a reminder to post their preventer inhaler use (Multimedia Appendix 12). At the end of the study, participants were emailed with instructions (Multimedia Appendix 13) on how to complete the follow-up measures.

Participants that completed the study, and posted on their site at least once a week were mailed a £20 (approximately $30) shopping voucher for participation (n = 82). Participants who completed at least the baseline and follow-up measures were mailed a £10 (approximately $15) voucher (n = 23). This approach was taken to allow as many participants as possible an opportunity to fill out the follow-up questionnaire and reduce bias in the sample.

5-viii) Mode of delivery, features/functionalities/components of the intervention and comparator, and the theoretical framework

Describe mode of delivery, features/functionalities/components of the intervention and comparator, and the theoretical framework [6] used to design them (instructional strategy [1], behaviour change techniques, persuasive features, etc., see e.g., [7, 8] for terminology). This includes an in-depth description of the content (including where it is coming from and who developed it) [1],” whether [and how] it is tailored to individual circumstances and allows users to track their progress and receive feedback” [6]. This also includes a description of communication delivery channels and – if computer-mediated communication is a component – whether communication was synchronous or asynchronous [6]. It also includes information on presentation strategies [1], including page design principles, average amount of text on pages, presence of hyperlinks to other resources, etc. [1].

	1	2	3	4	5	
subitem not at all important	○	○	○	○	○	essential

Does your paper address subitem 5-viii? *

Copy and paste relevant sections from the manuscript (include quotes in quotation marks "like this" to indicate direct quotes from your manuscript), or elaborate on this item by providing additional information not in the ms, or briefly explain why the item is not applicable/relevant for your study

Theoretical framework
We predicted that participating in an online community would lead to greater medication adherence. The theoretical underpinnings of this prediction are Social Cognitive Theory [22, 23] and the stress and coping perspective of social support [24]. The effect of community website exposure [10] is also included in the theoretical framework of the intervention. According to Social Cognitive Theory [22, 23], individuals can learn by observing the actions of others. If those actions produce an effect that is beneficial to the individual being observed, those actions are more likely to be imitated. It was predicted that participants observing the adherence of other patients will themselves improve adherence to inhaled corticosteroid (ICS) treatment. Participants reading other patients' success stories regarding adherence or dealing with asthma more generally will learn from these stories and apply these lessons to their own life, improving adherence.

Social support has been defined as the quality and structure of an individual's relationships, and is associated with improvements in adherence to medication regimes [20]. As is predicted in the stress-buffering perspective of social support [24], the perception of having socially supportive relationships and the support that participants actually receive will reduce stress associated with adherence and asthma improving adherence and overall health.

Intervention
The online community was creating using Wordpress [29], an open-source content management system, and Buddypress [30] social networking features (Multimedia Appendix 8). There were four primary intervention components: (a) a homepage that displayed all site activity in a rolling status board, (b) a group diary for posting preventer use, (c) a page for posting questions and answers about asthma, and (d) a profile page.

The main actions participants could take on the online community were posting or creating comments. The intervention was developed to be accessible by smartphone web browsers, as well as by desktop versions.

The effect of membership in the online community is dependent on the extent members are using the website of the online community itself. Site engagement was a barrier for participants in the pilot. In order to create a website which closely resembled popular patient communities such as PatientsLikeMe, we created a site discussion section dedicated to posting questions regarding asthma. Such features have been shown to be beneficial to engagement in previous studies [9].

5-ix) Describe use parameters

Describe use parameters (e.g., intended "doses" and optimal timing for use). Clarify what instructions or recommendations were given to the user, e.g., regarding timing, frequency, heaviness of use, if any, or was the intervention used ad libitum.

	1	2	3	4	5	
subitem not at all important	○	○	○	○	○	essential

Does your paper address subitem 5-ix?

Copy and paste relevant sections from the manuscript (include quotes in quotation marks "like this" to indicate direct quotes from your manuscript), or elaborate on this item by providing additional information not in the ms, or briefly explain why the item is not applicable/relevant for your study

Procedure
After randomization, participants were emailed instructions on how to use their site (Multimedia Appendix 10, 11). Participants were then prompted to log into their respective websites. Before logging in, participants created a username and password for their sites. Participants randomized to the diary created a PIN which they were required to input whenever they posted preventer use. Participants in both conditions were quasi-anonymous. Since registration required an email confirmation, it is unlikely that a single individual operated multiple accounts.

During the trial, a weekly email was automated to send to participants indicating which week the trial was on, with a reminder to post their preventer inhaler use (Multimedia Appendix 12). At the end of the study, participants were emailed with instructions (Multimedia Appendix 13) on how to complete the follow-up measures.

Participants that completed the study, and posted on their site at least once a week were mailed a £20 (approximately $30) shopping voucher for participation (n = 82). Participants who completed at least the baseline and follow-up measures were mailed a £10 (approximately $15) voucher (n = 23). This approach was taken to allow as many participants as possible an opportunity to fill out the follow-up questionnaire and reduce bias in the sample.

5-x) Clarify the level of human involvement

Clarify the level of human involvement (care providers or health professionals, also technical assistance) in the e-intervention or as co-intervention (detail number and expertise of professionals involved, if any, as well as “type of assistance offered, the timing and frequency of the support, how it is initiated, and the medium by which the assistance is delivered”. It may be necessary to distinguish between the level of human involvement required for the trial, and the level of human involvement required for a routine application outside of a RCT setting (discuss under item 21 – generalizability).

	1	2	3	4	5	
subitem not at all important	○	○	○	○	○	essential

Does your paper address subitem 5-x?

Copy and paste relevant sections from the manuscript (include quotes in quotation marks "like this" to indicate direct quotes from your manuscript), or elaborate on this item by providing additional information not in the ms, or briefly explain why the item is not applicable/relevant for your study

Procedure
After randomization, participants were emailed instructions on how to use their site (Multimedia Appendix 10, 11). Participants were then prompted to log into their respective websites. Before logging in, participants created a username and password for their sites. Participants randomized to the diary created a PIN which they were required to input whenever they posted preventer use. Participants in both conditions were quasi-anonymous. Since registration required an email confirmation, it is unlikely that a single individual operated multiple accounts.

During the trial, a weekly email was automated to send to participants indicating which week the trial was on, with a reminder to post their preventer inhaler use (Multimedia Appendix 12). At the end of the study, participants were emailed with instructions (Multimedia Appendix 13) on how to complete the follow-up measures.

Participants that completed the study, and posted on their site at least once a week were mailed a £20 (approximately $30) shopping voucher for participation (n = 82). Participants who completed at least the baseline and follow-up measures were mailed a £10 (approximately $15) voucher (n = 23). This approach was taken to allow as many participants as possible an opportunity to fill out the follow-up questionnaire and reduce bias in the sample.

5-xi) Report any prompts/reminders used

Report any prompts/reminders used: Clarify if there were prompts (letters, emails, phone calls, SMS) to use the application, what triggered them, frequency etc. It may be necessary to distinguish between the level of prompts/reminders required for the trial, and the level of prompts/reminders for a routine application outside of a RCT setting (discuss under item 21 – generalizability).

	1	2	3	4	5	
subitem not at all important	○	○	○	○	○	essential

Does your paper address subitem 5-xi? *

Copy and paste relevant sections from the manuscript (include quotes in quotation marks "like this" to indicate direct quotes from your manuscript), or elaborate on this item by providing additional information not in the ms, or briefly explain why the item is not applicable/relevant for your study

Procedure
After randomization, participants were emailed instructions on how to use their site (Multimedia Appendix 10, 11). Participants were then prompted to log into their respective websites. Before logging in, participants created a username and password for their sites. Participants randomized to the diary created a PIN which they were required to input whenever they posted preventer use. Participants in both conditions were quasi-anonymous. Since registration required an email confirmation, it is unlikely that a single individual operated multiple accounts.

During the trial, a weekly email was automated to send to participants indicating which week the trial was on, with a reminder to post their preventer inhaler use (Multimedia Appendix 12). At the end of the study, participants were emailed with instructions (Multimedia Appendix 13) on how to complete the follow-up measures.

Participants that completed the study, and posted on their site at least once a week were mailed a £20 (approximately $30) shopping voucher for participation (n = 82). Participants who completed at least the baseline and follow-up measures were mailed a £10 (approximately $15) voucher (n = 23). This approach was taken to allow as many participants as possible an opportunity to fill out the follow-up questionnaire and reduce bias in the sample.

5-xii) Describe any co-interventions (incl. training/support)

Describe any co-interventions (incl. training/support): Clearly state any interventions that are provided in addition to the targeted eHealth intervention, as ehealth intervention may not be designed as stand-alone intervention. This includes training sessions and support [1]. It may be necessary to distinguish between the level of training required for the trial, and the level of training for a routine application outside of a RCT setting (discuss under item 21 – generalizability.

	1	2	3	4	5	
subitem not at all important	○	○	○	○	○	essential

Does your paper address subitem 5-xii? *

Copy and paste relevant sections from the manuscript (include quotes in quotation marks "like this" to indicate direct quotes from your manuscript), or elaborate on this item by providing additional information not in the ms, or briefly explain why the item is not applicable/relevant for your study

N/A

6a) Completely defined pre-specified primary and secondary outcome measures, including how and when they were assessed

Does your paper address CONSORT subitem 6a? *

Copy and paste relevant sections from the manuscript (include quotes in quotation marks "like this" to indicate direct quotes from your manuscript), or elaborate on this item by providing additional information not in the ms, or briefly explain why the item is not applicable/relevant for your study

Baseline Measures
Baseline measures were included as part of the eligibility screening. Participants completed an online survey (Multimedia Appendix 6) that included questions about gender, age, previous social networking use, and prescriptions.

Preventer adherence in both conditions was self-reported using the six-item Simplified Medication Adherence Questionnaire (SMAQ; [28], Multimedia Appendix 7), a common, validated measure of medication adherence. In the questionnaire, the SMAQ refers generally to all medicine. For example, the first item of the SMAQ reads, “Do you ever forget to take your medicine?” For this study all instances of the word, “medicine” was changed to “asthma preventer medication”. This small change was was unlikely to have affected the measure.

The SMAQ was then recalculated where all variables became dichotomous by rescoring item 4 of the SMAQ (“Thinking about the last week, how often have you not taken your asthma preventer medicine as prescribed?”) where more than two missed uses was equal to nonadherent. The rescored SMAQ formed a reliable measure (Cronbach’s alpha = .72), and all items were averaged at baseline. Missing values were given to any participant with fewer than two filled out SMAQ items.

Follow-up Measures
The SMAQ was also used to measure ICS preventer adherence at follow-up (SMAQ-T2). Just as at baseline, the measure was then rescored where all variables became dichotomous. The SMAQ-T2 formed a reliable measure (Cronbach’s alpha = .69), and all items were averaged. Missing values were assigned to any participant with fewer than two filled out SMAQ-T2 items. Intention-to-treat (ITT) analyses were conducted where SMAQ-T1 means were carried forward to follow-up for participants who did not complete the follow-up measures.

Participants in both conditions were instructed to report their preventer use on their assigned website each time they used their preventers for nine weeks. The extent that participants adhered to these directions was calculated (Site Adherence). The total number of preventer puffs (Total Puffs) each participant reported having taken in each week was divided by the total number of preventer puffs prescribed in a given week (daily total number of puffs prescribed in one day multiplied by seven). This calculation produced a score of weekly site adherence for each participant. Thus, if a participant logged their preventer use every time they were prescribed to use their preventer, their Site Adherence would be 100%. The mean adherence across each of the nine weeks was averaged, forming an overall score of adherence to the site (Site Adherence).

6a-i) Online questionnaires: describe if they were validated for online use and apply CHERRIES items to describe how the questionnaires were designed/deployed

If outcomes were obtained through online questionnaires, describe if they were validated for online use and apply CHERRIES items to describe how the questionnaires were designed/deployed [9].

1 2 3 4 5

subitem not at all important ◯ ◯ ◯ ◯ ◯ essential

Does your paper address subitem 6a-i?

Copy and paste relevant sections from manuscript text

Baseline Measures
Baseline measures were included as part of the eligibility screening. Participants completed an online survey (Multimedia Appendix 6) that included questions about gender, age, previous social networking use, and prescriptions.

Preventer adherence in both conditions was self-reported using the six-item Simplified Medication Adherence Questionnaire (SMAQ; [28], Multimedia Appendix 7), a common, validated measure of medication adherence. In the questionnaire, the SMAQ refers generally to all medicine. For example, the first item of the SMAQ reads, “Do you ever forget to take your medicine?” For this study all instances of the word, “medicine” was changed to “asthma preventer medication”. This small change was was unlikely to have affected the measure.

The SMAQ was then recalculated where all variables became dichotomous by rescoring item 4 of the SMAQ (“Thinking about the last week, how often have you not taken your asthma preventer medicine as prescribed?”) where more than two missed uses was equal to nonadherent. The rescored SMAQ formed a reliable measure (Cronbach’s alpha = .72), and all items were averaged at baseline. Missing values were given to any participant with fewer than two filled out SMAQ items.

6a-ii) Describe whether and how “use” (including intensity of use/dosage) was defined/measured/monitored

Describe whether and how “use” (including intensity of use/dosage) was defined/measured/monitored (logins, logfile analysis, etc.). Use/adoption metrics are important process outcomes that should be reported in any ehealth trial.

	1	2	3	4	5	
subitem not at all important	◯	◯	◯	◯	◯	essential

Does your paper address subitem 6a-ii?

Copy and paste relevant sections from manuscript text

Baseline Measures
Baseline measures were included as part of the eligibility screening. Participants completed an online survey (Multimedia Appendix 6) that included questions about gender, age, previous social networking use, and prescriptions.

Preventer adherence in both conditions was self-reported using the six-item Simplified Medication Adherence Questionnaire (SMAQ; [28], Multimedia Appendix 7), a common, validated measure of medication adherence. In the questionnaire, the SMAQ refers generally to all medicine. For example, the first item of the SMAQ reads, “Do you ever forget to take your medicine?” For this study all instances of the word, “medicine” was changed to “asthma preventer medication”. This small change was was unlikely to have affected the measure.

The SMAQ was then recalculated where all variables became dichotomous by rescoring item 4 of the SMAQ (“Thinking about the last week, how often have you not taken your asthma preventer medicine as prescribed?”) where more than two missed uses was equal to nonadherent. The rescored SMAQ formed a reliable measure (Cronbach’s alpha = .72), and all items were averaged at baseline. Missing values were given to any participant with fewer than two filled out SMAQ items.

6a-iii) Describe whether, how, and when qualitative feedback from participants was obtained

Describe whether, how, and when qualitative feedback from participants was obtained (e.g., through emails, feedback forms, interviews, focus groups).

	1	2	3	4	5	
subitem not at all important	◯	◯	◯	◯	◯	essential

Does your paper address subitem 6a-iii?

Copy and paste relevant sections from manuscript text

N/A

6b) Any changes to trial outcomes after the trial commenced, with reasons

Does your paper address CONSORT subitem 6b? *

Copy and paste relevant sections from the manuscript (include quotes in quotation marks "like this" to indicate direct quotes from your manuscript), or elaborate on this item by providing additional

information not in the ms, or briefly explain why the item is not applicable/relevant for your study

N/A

7a) How sample size was determined

NPT: When applicable, details of whether and how the clustering by care provides or centers was addressed

7a-i) Describe whether and how expected attrition was taken into account when calculating the sample size

Describe whether and how expected attrition was taken into account when calculating the sample size.

	1	2	3	4	5	
subitem not at all important	◯	◯	◯	◯	◯	essential

Does your paper address subitem 7a-i?

Copy and paste relevant sections from manuscript title (include quotes in quotation marks "like this" to indicate direct quotes from your manuscript), or elaborate on this item by providing additional information not in the ms, or briefly explain why the item is not applicable/relevant for your study

Based on an expected medium effect size (d = .5), an alpha of .05 [one-tailed] and a power of 80% we calculated that a total of 102 participants would be needed to complete the study. In eHealth studies, attrition can vary considerably. We assumed a 50% dropout rate and thus recruited double the number of participants we needed.

7b) When applicable, explanation of any interim analyses and stopping guidelines

Does your paper address CONSORT subitem 7b? *

Copy and paste relevant sections from the manuscript (include quotes in quotation marks "like this" to indicate direct quotes from your manuscript), or elaborate on this item by providing additional information not in the ms, or briefly explain why the item is not applicable/relevant for your study

N/A

8a) Method used to generate the random allocation sequence

NPT: When applicable, how care providers were allocated to each trial group

Does your paper address CONSORT subitem 8a? *

Copy and paste relevant sections from the manuscript (include quotes in quotation marks "like this" to indicate direct quotes from your manuscript), or elaborate on this item by providing additional information not in the ms, or briefly explain why the item is not applicable/relevant for your study

"Participants were then randomized to the diary (n = 117) and online community (n = 99) conditions. Randomization occurred through a random number generator [26], yielding two unequal groups."

8b) Type of randomisation; details of any restriction (such as blocking and block size)

Does your paper address CONSORT subitem 8b? *

Copy and paste relevant sections from the manuscript (include quotes in quotation marks "like this" to indicate direct quotes from your manuscript), or elaborate on this item by providing additional information not in the ms, or briefly explain why the item is not applicable/relevant for your study

"Participants were then randomized to the diary (n = 117) and online community (n = 99) conditions. Randomization occurred through a random number generator [26], yielding two unequal groups."

9) Mechanism used to implement the random allocation sequence (such as sequentially numbered containers), describing any steps taken to conceal the sequence until interventions were assigned

Does your paper address CONSORT subitem 9? *

Copy and paste relevant sections from the manuscript (include quotes in quotation marks "like this" to indicate direct quotes from your manuscript), or elaborate on this item by providing additional information not in the ms, or briefly explain why the item is not applicable/relevant for your study

"Participants were then randomized to the diary (n = 117) and online community (n = 99) conditions. Randomization occurred through a random number generator [26], yielding two unequal groups."

10) Who generated the random allocation sequence, who enrolled participants, and who assigned participants to interventions

Does your paper address CONSORT subitem 10? *

Copy and paste relevant sections from the manuscript (include quotes in quotation marks "like this" to indicate direct quotes from your manuscript), or elaborate on this item by providing additional information not in the ms, or briefly explain why the item is not applicable/relevant for your study

"Participants were then randomized to the diary (n = 117) and online community (n = 99) conditions. Randomization occurred through a random number generator [26], yielding two unequal groups."

11a) If done, who was blinded after assignment to interventions (for example, participants, care providers, those assessing outcomes) and how

NPT: Whether or not administering co-interventions were blinded to group assignment

11a-i) Specify who was blinded, and who wasn't

Specify who was blinded, and who wasn't. Usually, in web-based trials it is not possible to blind the participants [1, 3] (this should be clearly acknowledged), but it may be possible to blind outcome assessors, those doing data analysis or those administering co-interventions (if any).

	1	2	3	4	5	
subitem not at all important	○	○	○	○	○	essential

Does your paper address subitem 11a-i? *

Copy and paste relevant sections from the manuscript (include quotes in quotation marks "like this" to indicate direct quotes from your manuscript), or elaborate on this item by providing additional information not in the ms, or briefly explain why the item is not applicable/relevant for your study

An active control was used to test clearly the effect of the community on adherence, and prevent participants from guessing if they were in the group of interest. The study was carried out for nine weeks, between June 24th, 2013 and August 26th, 2013. The trial conformed to the Consolidated Standards of Reporting Trials (CONSORT)-eHealth Checklist (Multimedia Appendix 1) [34].

11a-ii) Discuss e.g., whether participants knew which intervention was the "intervention of interest" and which one was the "comparator"

Informed consent procedures (4a-ii) can create biases and certain expectations - discuss e.g., whether participants knew which intervention was the "intervention of interest" and which one was the "comparator".

	1	2	3	4	5	
subitem not at all important	○	○	○	○	○	essential

Does your paper address subitem 11a-ii?

Copy and paste relevant sections from the manuscript (include quotes in quotation marks "like this" to indicate direct quotes from your manuscript), or elaborate on this item by providing additional information not in the ms, or briefly explain why the item is not applicable/relevant for your study

An active control was used to test clearly the effect of the community on adherence, and prevent participants from guessing if they were in the group of interest. The study was carried out for nine weeks, between June 24th, 2013 and August 26th, 2013. The trial conformed to the Consolidated Standards of Reporting Trials (CONSORT)-eHealth Checklist (Multimedia Appendix 1) [34].

11b) If relevant, description of the similarity of interventions

(this item is usually not relevant for ehealth trials as it refers to similarity of a placebo or sham intervention to a active medication/intervention)

Does your paper address CONSORT subitem 11b? *

Copy and paste relevant sections from the manuscript (include quotes in quotation marks "like this" to indicate direct quotes from your manuscript), or elaborate on this item by providing additional information not in the ms, or briefly explain why the item is not applicable/relevant for your study

N/A

12a) Statistical methods used to compare groups for primary and secondary outcomes

NPT: When applicable, details of whether and how the clustering by care providers or centers was addressed

Does your paper address CONSORT subitem 12a? *

Copy and paste relevant sections from the manuscript (include quotes in quotation marks "like this" to indicate direct quotes from your manuscript), or elaborate on this item by providing additional information not in the ms, or briefly explain why the item is not applicable/relevant for your study

Statistical Analysis
Sample Size Calculation
Based on an expected medium effect size (d = .5), an alpha of .05 [one-tailed] and a power of 80% we calculated that a total of 102 participants would be needed to complete the study. In eHealth studies, attrition can vary considerably. We assumed a 50% dropout rate and thus recruited double the number of participants we needed.
Analysis
First, descriptive statistics were calculated for all variables (gender, age, Total Puffs, Site Adherence, SMAQ-T1, SMAQ-T2, and SMAQ-T2 ITT) and examined across the whole sample and for each condition.

Next, we examined the baseline variables (gender, age, and SMAQ-T1) to determine if conditions were matched (i.e., to test if randomization had been successful) using multivariate analysis of variance (MANOVA) with condition as the independent variable. We then examined differences at follow-up by condition on the variables measured at that time point (SMAQ-T2-ITT, SMAQ-T2, Total Puffs, and Site Adherence), controlling for any baseline differences where necessary. In order to avoid problems of multiple significance testing, univariate effects were only explored where multivariate effects were significant.

Statistical analyses were conducted using IBM SPSS Statistics 20.0 (IBM Corp., Armonk, NY).

12a-i) Imputation techniques to deal with attrition / missing values

Imputation techniques to deal with attrition / missing values: Not all participants will use the intervention/comparator as intended and attrition is typically high in ehealth trials. Specify how participants who did not use the application or dropped out from the trial were treated in the statistical analysis (a complete case analysis is strongly discouraged, and simple imputation techniques such as LOCF may also be problematic [4]).

	1	2	3	4	5	
subitem not at all important	○	○	○	○	○	essential

Does your paper address subitem 12a-i? *

Copy and paste relevant sections from the manuscript (include quotes in quotation marks "like this" to indicate direct quotes from your manuscript), or elaborate on this item by providing additional information not in the ms, or briefly explain why the item is not applicable/relevant for your study

Statistical Analysis
Sample Size Calculation
Based on an expected medium effect size (d = .5), an alpha of .05 [one-tailed] and a power of 80% we calculated that a total of 102 participants would be needed to complete the study. In eHealth studies, attrition can vary considerably. We assumed a 50% dropout rate and thus recruited double the number of participants we needed.
Analysis
First, descriptive statistics were calculated for all variables (gender, age, Total Puffs, Site Adherence, SMAQ-T1, SMAQ-T2, and SMAQ-T2 ITT) and examined across the whole sample and for each condition.

Next, we examined the baseline variables (gender, age, and SMAQ-T1) to determine if conditions were matched (i.e., to test if randomization had been successful) using multivariate analysis of variance (MANOVA) with condition as the independent variable. We then examined differences at follow-up by condition on the variables measured at that time point (SMAQ-T2-ITT, SMAQ-T2, Total Puffs, and Site Adherence), controlling for any baseline differences where necessary. In order to avoid problems of multiple significance testing, univariate effects were only explored where multivariate effects were significant.

Statistical analyses were conducted using IBM SPSS Statistics 20.0 (IBM Corp., Armonk, NY).

12b) Methods for additional analyses, such as subgroup analyses and adjusted analyses

Does your paper address CONSORT subitem 12b? *

Copy and paste relevant sections from the manuscript (include quotes in quotation marks "like this" to indicate direct quotes from your manuscript), or elaborate on this item by providing additional information not in the ms, or briefly explain why the item is not applicable/relevant for your study

Statistical Analysis
Sample Size Calculation
Based on an expected medium effect size (d = .5), an alpha of .05 [one-tailed] and a power of 80% we calculated that a total of 102 participants would be needed to complete the study. In eHealth studies, attrition can vary considerably. We assumed a 50% dropout rate and thus recruited double the number of participants we needed.
Analysis
First, descriptive statistics were calculated for all variables (gender, age, Total Puffs, Site Adherence, SMAQ-T1, SMAQ-T2, and SMAQ-T2 ITT) and examined across the whole sample and for each condition.

Next, we examined the baseline variables (gender, age, and SMAQ-T1) to determine if conditions were matched (i.e., to test if randomization had been successful) using multivariate analysis of variance (MANOVA) with condition as the independent variable. We then examined differences at follow-up by condition on the variables measured at that time point (SMAQ-T2-ITT, SMAQ-T2, Total Puffs, and Site Adherence), controlling for any baseline differences where necessary. In order to avoid problems of multiple significance testing, univariate effects were only explored where multivariate effects were significant.

Statistical analyses were conducted using IBM SPSS Statistics 20.0 (IBM Corp., Armonk, NY).

X26) REB/IRB Approval and Ethical Considerations [recommended as subheading under "Methods"] (not a CONSORT item)

X26-i) Comment on ethics committee approval

	1	2	3	4	5	
subitem not at all important	○	○	○	○	○	essential

Does your paper address subitem X26-i?

Copy and paste relevant sections from the manuscript (include quotes in quotation marks "like this" to indicate direct quotes from your manuscript), or elaborate on this item by providing additional information not in the ms, or briefly explain why the item is not applicable/relevant for your study

Human Subjects and Trial Registration
The University of Leeds School of Psychology Ethics Committee approved this study (ethics reference number 13-0096). All participants gave online consent. The details of the trial were made public in advance: ISRCTN trial registration number was 29399269.
Results

x26-ii) Outline informed consent procedures

Outline informed consent procedures e.g., if consent was obtained offline or online (how? Checkbox,

etc.?), and what information was provided (see 4a-ii). See [6] for some items to be included in informed consent documents.

	1	2	3	4	5	
subitem not at all important	◯	◯	◯	◯	◯	essential

Does your paper address subitem X26-ii?

Copy and paste relevant sections from the manuscript (include quotes in quotation marks "like this" to indicate direct quotes from your manuscript), or elaborate on this item by providing additional information not in the ms, or briefly explain why the item is not applicable/relevant for your study

Eligibility Screening and Consent
Nine hundred and thirty-six participants responded to the eligibility questionnaire. Participants were excluded from the study if they failed to complete the eligibility questionnaire (n = 256) or baseline measures (n = 221), did not have asthma (n = 105), were not prescribed an ICS preventer inhaler (n = 87), or had previously taken the pilot (n = 9). After screening, a total of 251 participants were eligible for study inclusion. See Figure 1 for details.

Participants were automatically taken to the information sheet (Multimedia Appendix 4) and were asked to provide informed consent (Multimedia Appendix 5). Thirty-five participants refused to provide consent and were eliminated from the study, leaving 216 eligible participants. Participants were then randomized to the diary (n = 117) and online community (n = 99) conditions. Randomization occurred through a random number generator [26], yielding two unequal groups. The online screening survey was administered through Qualtrics [27], a subscription-based online survey software suite.

X26-iii) Safety and security procedures

Safety and security procedures, incl. privacy considerations, and any steps taken to reduce the likelihood or detection of harm (e.g., education and training, availability of a hotline)

	1	2	3	4	5	
subitem not at all important	◯	◯	◯	◯	◯	essential

Does your paper address subitem X26-iii?

Copy and paste relevant sections from the manuscript (include quotes in quotation marks "like this" to indicate direct quotes from your manuscript), or elaborate on this item by providing additional information not in the ms, or briefly explain why the item is not applicable/relevant for your study

Eligibility Screening and Consent
Nine hundred and thirty-six participants responded to the eligibility questionnaire. Participants were excluded from the study if they failed to complete the eligibility questionnaire (n = 256) or baseline measures (n = 221), did not have asthma (n = 105), were not prescribed an ICS preventer inhaler (n = 87), or had previously taken the pilot (n = 9). After screening, a total of 251 participants were eligible for study inclusion. See Figure 1 for details.

Participants were automatically taken to the information sheet (Multimedia Appendix 4) and were asked to provide informed consent (Multimedia Appendix 5). Thirty-five participants refused to provide consent and were eliminated from the study, leaving 216 eligible participants. Participants were then randomized to the diary (n = 117) and online community (n = 99) conditions. Randomization occurred through a random number generator [26], yielding two unequal groups. The online screening survey was administered through Qualtrics [27], a subscription-based online survey software suite.

RESULTS

13a) For each group, the numbers of participants who were randomly assigned, received intended treatment, and were analysed for the primary outcome

NPT: The number of care providers or centers performing the intervention in each group and the number of patients treated by each care provider in each center

Does your paper address CONSORT subitem 13a? *

Copy and paste relevant sections from the manuscript (include quotes in quotation marks "like this" to indicate direct quotes from your manuscript), or elaborate on this item by providing additional information not in the ms, or briefly explain why the item is not applicable/relevant for your study

Results
Attrition
Of the 216 participants who met our inclusion criteria, only 103 participants fully completed the study, 64 (62%) of these were from the control arm. A chi-square test indicated that drop out was higher in the intervention condition (60/99, 61%) than the control condition (53/117, 45%), $\chi 2$ (2, n = 216) = 5.037, P = .03.

We also tested whether the sample who completed the study were representative of the initial sample on baseline measures of gender, age, and SMAQ-T1. MANOVA revealed a significant difference between groups, Wilk's Lambda =.944, F(3, 212) = 4.190, P = .007. Examination of the univariate effects revealed significant effects for SMAQ-T1 (F(1,214) = 4.48, P = .04), and gender (F(1,214) = 7.20, P = .008), but no effect for age (F(1,214) = 0.72). On average, completers scored higher on the SMAQ at baseline (1.49) than non-completers (1.40). Higher SMAQ scores indicate lower preventer adherence. Completers were also much more likely to be female (79.6%) than non-completers (61.1%).

Our attrition analyses indicated that the sample completing the study was not fully representative of those starting the study and therefore analyses based on completers should be treated with caution.

Descriptive Statistics
Participants were mostly women, and although ages ranged from 18 to 64 years, the average participant was in their late twenties (Table 1). There was generally an even mixture of adherent and nonadherent participants.

Table 1 also shows that in general the control and intervention arms showed few differences except in relation to site adherence measures.

Primary Outcomes
MANOVA revealed that there were no significant differences between condition across the various dependent measures taken at baseline [Wilks' Lambda = .983, F(3, 212) = 1.19, P = .32], indicating that groups were matched and supported the success of randomization in creating two equivalent groups. Given the lack of difference between conditions on baseline measures we did not control for any baseline measures in subsequent analyses of the follow-up measures.

MANOVA of group differences at follow-up revealed that there was a significant effect of condition, Wilks' Lambda = .815, F(3, 89) = 6.723, P < .001. Examination of the univariate effects revealed no significant differences between the control and intervention conditions on SMAQ when based on ITT analyses including all participants (F(1,216) = 0.03, ns) or when using only participants completing the follow-up measure of SMAQ (F(1, 91) = 0.03). There were however significant differences for site adherence (F(1, 91) = 14.76, P < .001) and total puffs (F(1, 91) = 18.31, P < .001). These differences reflected the higher levels of total puffs and site adherence in the control compared to the intervention condition (Table 1).

13b) For each group, losses and exclusions after

randomisation, together with reasons

Does your paper address CONSORT subitem 13b? (NOTE: Preferably, this is shown in a CONSORT flow diagram) *

Copy and paste relevant sections from the manuscript (include quotes in quotation marks "like this" to indicate direct quotes from your manuscript), or elaborate on this item by providing additional information not in the ms, or briefly explain why the item is not applicable/relevant for your study

See Figure 1.

13b-i) Attrition diagram

Strongly recommended: An attrition diagram (e.g., proportion of participants still logging in or using the intervention/comparator in each group plotted over time, similar to a survival curve) or other figures or tables demonstrating usage/dose/engagement.

	1	2	3	4	5	
subitem not at all important	○	○	○	○	○	essential

Does your paper address subitem 13b-i?

Copy and paste relevant sections from the manuscript or cite the figure number if applicable (include quotes in quotation marks "like this" to indicate direct quotes from your manuscript), or elaborate on this item by providing additional information not in the ms, or briefly explain why the item is not applicable/relevant for your study

See Figure 2.

14a) Dates defining the periods of recruitment and follow-up

Does your paper address CONSORT subitem 14a? *

Copy and paste relevant sections from the manuscript (include quotes in quotation marks "like this" to indicate direct quotes from your manuscript), or elaborate on this item by providing additional information not in the ms, or briefly explain why the item is not applicable/relevant for your study

The study was carried out for nine weeks, between June 24th, 2013 and August 26th, 2013. The trial conformed to the Consolidated Standards of Reporting Trials (CONSORT)-eHealth Checklist (Multimedia Appendix 1) [34].

14a-i) Indicate if critical "secular events" fell into the study period

Indicate if critical "secular events" fell into the study period, e.g., significant changes in Internet resources available or "changes in computer hardware or Internet delivery resources"

	1	2	3	4	5	
subitem not at all important	◯	◯	◯	◯	◯	essential

Does your paper address subitem 14a-i?

Copy and paste relevant sections from the manuscript (include quotes in quotation marks "like this" to indicate direct quotes from your manuscript), or elaborate on this item by providing additional information not in the ms, or briefly explain why the item is not applicable/relevant for your study

N/A

14b) Why the trial ended or was stopped (early)

Does your paper address CONSORT subitem 14b? *

Copy and paste relevant sections from the manuscript (include quotes in quotation marks "like this" to indicate direct quotes from your manuscript), or elaborate on this item by providing additional information not in the ms, or briefly explain why the item is not applicable/relevant for your study

N/A

15) A table showing baseline demographic and clinical characteristics for each group

NPT: When applicable, a description of care providers (case volume, qualification, expertise, etc.) and centers (volume) in each group

Does your paper address CONSORT subitem 15? *

Copy and paste relevant sections from the manuscript (include quotes in quotation marks "like this" to indicate direct quotes from your manuscript), or elaborate on this item by providing additional information not in the ms, or briefly explain why the item is not applicable/relevant for your study

See Table 1.

15-i) Report demographics associated with digital divide issues

In ehealth trials it is particularly important to report demographics associated with digital divide issues, such as age, education, gender, social-economic status, computer/Internet/ehealth literacy of the participants, if known.

	1	2	3	4	5	
subitem not at all important	○	○	○	○	○	essential

Does your paper address subitem 15-i? *

Copy and paste relevant sections from the manuscript (include quotes in quotation marks "like this" to indicate direct quotes from your manuscript), or elaborate on this item by providing additional information not in the ms, or briefly explain why the item is not applicable/relevant for your study

See Table 1.

16) For each group, number of participants (denominator) included in each analysis and whether the analysis was by original assigned groups

16-i) Report multiple “denominators” and provide definitions

Report multiple “denominators” and provide definitions: Report N’s (and effect sizes) “across a range of study participation [and use] thresholds” [1], e.g., N exposed, N consented, N used more than x times, N used more than y weeks, N participants “used” the intervention/comparator at specific pre-defined time points of interest (in absolute and relative numbers per group). Always clearly define “use” of the intervention.

	1	2	3	4	5	
subitem not at all important	○	○	○	○	○	essential

Does your paper address subitem 16-i? *

Copy and paste relevant sections from the manuscript (include quotes in quotation marks "like this" to indicate direct quotes from your manuscript), or elaborate on this item by providing additional information not in the ms, or briefly explain why the item is not applicable/relevant for your study

Primary Outcomes
MANOVA revealed that there were no significant differences between condition across the various dependent measures taken at baseline [Wilks' Lambda = .983, F(3, 212) = 1.19, P = .32], indicating that groups were matched and supported the success of randomization in creating two equivalent groups. Given the lack of difference between conditions on baseline measures we did not control for any baseline measures in subsequent analyses of the follow-up measures.

MANOVA of group differences at follow-up revealed that there was a significant effect of condition, Wilks' Lambda = .815, F(3, 89) = 6.723, P < .001. Examination of the univariate effects revealed no significant differences between the control and intervention conditions on SMAQ when based on ITT analyses including all participants (F(1,216) = 0.03, ns) or when using only participants completing the follow-up measure of SMAQ (F(1, 91) = 0.03). There were however significant differences for site adherence (F(1, 91) = 14.76, P < .001) and total puffs (F(1, 91) = 18.31, P < .001). These differences reflected the higher levels of total puffs and site adherence in the control compared to the intervention condition (Table 1).
Site Adherence over Nine Weeks
Further detailed examination of the weekly site adherence means over the nine-week intervention indicated a substantial difference between conditions in adherence at Week 1, with 41.7% in the control, compared to 11.3% in the intervention. Figure 2 shows that site adherence was most different between groups at Week 1,and fell at a much greater rate across weeks in the control than in the intervention, which stayed relatively consistent.

Figure 2. Change in Site Adherence over nine weeks by condition.

16-ii) Primary analysis should be intent-to-treat

Primary analysis should be intent-to-treat, secondary analyses could include comparing only "users", with the appropriate caveats that this is no longer a randomized sample (see 18-i).

	1	2	3	4	5	
subitem not at all important	○	○	○	○	○	essential

Does your paper address subitem 16-ii?

Copy and paste relevant sections from the manuscript (include quotes in quotation marks "like this" to indicate direct quotes from your manuscript), or elaborate on this item by providing additional information not in the ms, or briefly explain why the item is not applicable/relevant for your study

Primary Outcomes
MANOVA revealed that there were no significant differences between condition across the various dependent measures taken at baseline [Wilks' Lambda = .983, $F(3, 212) = 1.19$, $P = .32$], indicating that groups were matched and supported the success of randomization in creating two equivalent groups. Given the lack of difference between conditions on baseline measures we did not control for any baseline measures in subsequent analyses of the follow-up measures.

MANOVA of group differences at follow-up revealed that there was a significant effect of condition, Wilks' Lambda = .815, $F(3, 89) = 6.723$, $P < .001$. Examination of the univariate effects revealed no significant differences between the control and intervention conditions on SMAQ when based on ITT analyses including all participants ($F(1,216) = 0.03$, ns) or when using only participants completing the follow-up measure of SMAQ ($F(1, 91) = 0.03$). There were however significant differences for site adherence ($F(1, 91) = 14.76$, $P < .001$) and total puffs ($F(1, 91) = 18.31$, $P < .001$). These differences reflected the higher levels of total puffs and site adherence in the control compared to the intervention condition (Table 1).
Site Adherence over Nine Weeks
Further detailed examination of the weekly site adherence means over the nine-week intervention indicated a substantial difference between conditions in adherence at Week 1, with 41.7% in the control, compared to 11.3% in the intervention. Figure 2 shows that site adherence was most different between groups at Week 1,and fell at a much greater rate across weeks in the control than in the intervention, which stayed relatively consistent.

Figure 2. Change in Site Adherence over nine weeks by condition.

17a) For each primary and secondary outcome, results for each group, and the estimated effect size and its precision (such as 95% confidence interval)

Does your paper address CONSORT subitem 17a? *

Copy and paste relevant sections from the manuscript (include quotes in quotation marks "like this" to indicate direct quotes from your manuscript), or elaborate on this item by providing additional information not in the ms, or briefly explain why the item is not applicable/relevant for your study

Primary Outcomes
MANOVA revealed that there were no significant differences between condition across the various dependent measures taken at baseline [Wilks' Lambda = .983, $F(3, 212) = 1.19$, $P = .32$], indicating that groups were matched and supported the success of randomization in creating two equivalent groups. Given the lack of difference between conditions on baseline measures we did not control for any baseline measures in subsequent analyses of the follow-up measures.

MANOVA of group differences at follow-up revealed that there was a significant effect of condition, Wilks' Lambda = .815, $F(3, 89) = 6.723$, $P < .001$. Examination of the univariate effects revealed no significant differences between the control and intervention conditions on SMAQ when based on ITT analyses including all participants ($F(1,216) = 0.03$, ns) or when using only participants completing the follow-up measure of SMAQ ($F(1, 91) = 0.03$). There were however significant differences for site adherence ($F(1, 91) = 14.76$, $P < .001$) and total puffs ($F(1, 91) = 18.31$, $P < .001$). These differences reflected the higher levels of total puffs and site adherence in the control compared to the intervention condition (Table 1).
Site Adherence over Nine Weeks
Further detailed examination of the weekly site adherence means over the nine-week intervention indicated a substantial difference between conditions in adherence at Week 1, with 41.7% in the control, compared to 11.3% in the intervention. Figure 2 shows that site adherence was most different between groups at Week 1,and fell at a much greater rate across weeks in the control than in the intervention, which stayed relatively consistent.

Figure 2. Change in Site Adherence over nine weeks by condition.

17a-i) Presentation of process outcomes such as metrics of use and intensity of use

In addition to primary/secondary (clinical) outcomes, the presentation of process outcomes such as metrics of use and intensity of use (dose, exposure) and their operational definitions is critical. This does not only refer to metrics of attrition (13-b) (often a binary variable), but also to more continuous exposure metrics such as "average session length". These must be accompanied by a technical description how a metric like a "session" is defined (e.g., timeout after idle time) [1] (report under item 6a).

	1	2	3	4	5	
subitem not at all important	○	○	○	○	○	essential

Does your paper address subitem 17a-i?

Copy and paste relevant sections from the manuscript (include quotes in quotation marks "like this" to indicate direct quotes from your manuscript), or elaborate on this item by providing additional information not in the ms, or briefly explain why the item is not applicable/relevant for your study

Primary Outcomes
MANOVA revealed that there were no significant differences between condition across the various dependent measures taken at baseline [Wilks' Lambda = .983, $F(3, 212) = 1.19$, $P = .32$], indicating that groups were matched and supported the success of randomization in creating two equivalent groups. Given the lack of difference between conditions on baseline measures we did not control for any baseline measures in subsequent analyses of the follow-up measures.

MANOVA of group differences at follow-up revealed that there was a significant effect of condition, Wilks' Lambda = .815, $F(3, 89) = 6.723$, $P < .001$. Examination of the univariate effects revealed no significant differences between the control and intervention conditions on SMAQ when based on ITT analyses including all participants ($F(1,216) = 0.03$, ns) or when using only participants completing the follow-up measure of SMAQ ($F(1, 91) = 0.03$). There were however significant differences for site adherence ($F(1, 91) = 14.76$, $P < .001$) and total puffs ($F(1, 91) = 18.31$, $P < .001$). These differences reflected the higher levels of total puffs and site adherence in the control compared to the intervention condition (Table 1).
Site Adherence over Nine Weeks
Further detailed examination of the weekly site adherence means over the nine-week intervention indicated a substantial difference between conditions in adherence at Week 1, with 41.7% in the control, compared to 11.3% in the intervention. Figure 2 shows that site adherence was most different between groups at Week 1,and fell at a much greater rate across weeks in the control than in the intervention, which stayed relatively consistent.

Figure 2. Change in Site Adherence over nine weeks by condition.

17b) For binary outcomes, presentation of both absolute and relative effect sizes is recommended

Does your paper address CONSORT subitem 17b? *

Copy and paste relevant sections from the manuscript (include quotes in quotation marks "like this" to indicate direct quotes from your manuscript), or elaborate on this item by providing additional information not in the ms, or briefly explain why the item is not applicable/relevant for your study

Primary Outcomes
MANOVA revealed that there were no significant differences between condition across the various dependent measures taken at baseline [Wilks' Lambda = .983, $F(3, 212) = 1.19$, $P = .32$], indicating that groups were matched and supported the success of randomization in creating two equivalent groups. Given the lack of difference between conditions on baseline measures we did not control for any baseline measures in subsequent analyses of the follow-up measures.

MANOVA of group differences at follow-up revealed that there was a significant effect of condition, Wilks' Lambda = .815, $F(3, 89) = 6.723$, $P < .001$. Examination of the univariate effects revealed no significant differences between the control and intervention conditions on SMAQ when based on ITT analyses including all participants ($F(1,216) = 0.03$, ns) or when using only participants completing the follow-up measure of SMAQ ($F(1, 91) = 0.03$). There were however significant differences for site adherence ($F(1, 91) = 14.76$, $P < .001$) and total puffs ($F(1, 91) = 18.31$, $P < .001$). These differences reflected the higher levels of total puffs and site adherence in the control compared to the intervention condition (Table 1).
Site Adherence over Nine Weeks
Further detailed examination of the weekly site adherence means over the nine-week intervention indicated a substantial difference between conditions in adherence at Week 1, with 41.7% in the control, compared to 11.3% in the intervention. Figure 2 shows that site adherence was most different between groups at Week 1,and fell at a much greater rate across weeks in the control than in the intervention, which stayed relatively consistent.

Figure 2. Change in Site Adherence over nine weeks by condition.

18) Results of any other analyses performed, including subgroup analyses and adjusted analyses, distinguishing pre-specified from exploratory

Does your paper address CONSORT subitem 18? *

Copy and paste relevant sections from the manuscript (include quotes in quotation marks "like this" to indicate direct quotes from your manuscript), or elaborate on this item by providing additional information not in the ms, or briefly explain why the item is not applicable/relevant for your study

Primary Outcomes
MANOVA revealed that there were no significant differences between condition across the various dependent measures taken at baseline [Wilks' Lambda = .983, $F(3, 212) = 1.19$, $P = .32$], indicating that groups were matched and supported the success of randomization in creating two equivalent groups. Given the lack of difference between conditions on baseline measures we did not control for any baseline measures in subsequent analyses of the follow-up measures.

MANOVA of group differences at follow-up revealed that there was a significant effect of condition, Wilks' Lambda = .815, $F(3, 89) = 6.723$, $P < .001$. Examination of the univariate effects revealed no significant differences between the control and intervention conditions on SMAQ when based on ITT analyses including all participants ($F(1,216) = 0.03$, ns) or when using only participants completing the follow-up measure of SMAQ ($F(1, 91) = 0.03$). There were however significant differences for site adherence ($F(1, 91) = 14.76$, $P < .001$) and total puffs ($F(1, 91) = 18.31$, $P < .001$). These differences reflected the higher levels of total puffs and site adherence in the control compared to the intervention condition (Table 1).
Site Adherence over Nine Weeks
Further detailed examination of the weekly site adherence means over the nine-week intervention indicated a substantial difference between conditions in adherence at Week 1, with 41.7% in the control, compared to 11.3% in the intervention. Figure 2 shows that site adherence was most different between groups at Week 1,and fell at a much greater rate across weeks in the control than in the intervention, which stayed relatively consistent.

Figure 2. Change in Site Adherence over nine weeks by condition.

18-i) Subgroup analysis of comparing only users

A subgroup analysis of comparing only users is not uncommon in ehealth trials, but if done, it must be stressed that this is a self-selected sample and no longer an unbiased sample from a randomized trial (see 16-iii).

	1	2	3	4	5	
subitem not at all important	○	○	○	○	○	essential

Does your paper address subitem 18-i?

Copy and paste relevant sections from the manuscript (include quotes in quotation marks "like this" to indicate direct quotes from your manuscript), or elaborate on this item by providing additional information not in the ms, or briefly explain why the item is not applicable/relevant for your study

Primary Outcomes
MANOVA revealed that there were no significant differences between condition across the various dependent measures taken at baseline [Wilks' Lambda = .983, F(3, 212) = 1.19, P = .32], indicating that groups were matched and supported the success of randomization in creating two equivalent groups. Given the lack of difference between conditions on baseline measures we did not control for any baseline measures in subsequent analyses of the follow-up measures.

MANOVA of group differences at follow-up revealed that there was a significant effect of condition, Wilks' Lambda = .815, F(3, 89) = 6.723, P < .001. Examination of the univariate effects revealed no significant differences between the control and intervention conditions on SMAQ when based on ITT analyses including all participants (F(1,216) = 0.03, ns) or when using only participants completing the follow-up measure of SMAQ (F(1, 91) = 0.03). There were however significant differences for site adherence (F(1, 91) = 14.76, P < .001) and total puffs (F(1, 91) = 18.31, P < .001). These differences reflected the higher levels of total puffs and site adherence in the control compared to the intervention condition (Table 1).
Site Adherence over Nine Weeks
Further detailed examination of the weekly site adherence means over the nine-week intervention indicated a substantial difference between conditions in adherence at Week 1, with 41.7% in the control, compared to 11.3% in the intervention. Figure 2 shows that site adherence was most different between groups at Week 1,and fell at a much greater rate across weeks in the control than in the intervention, which stayed relatively consistent.

Figure 2. Change in Site Adherence over nine weeks by condition.

19) All important harms or unintended effects in each group

(for specific guidance see CONSORT for harms)

Does your paper address CONSORT subitem 19? *

Copy and paste relevant sections from the manuscript (include quotes in quotation marks "like this" to indicate direct quotes from your manuscript), or elaborate on this item by providing additional information not in the ms, or briefly explain why the item is not applicable/relevant for your study

N/A

19-i) Include privacy breaches, technical problems

Include privacy breaches, technical problems. This does not only include physical “harm” to participants, but also incidents such as perceived or real privacy breaches [1], technical problems, and other unexpected/unintended incidents. “Unintended effects” also includes unintended positive effects [2].

	1	2	3	4	5	
subitem not at all important	○	○	○	○	○	essential

Does your paper address subitem 19-i?

Copy and paste relevant sections from the manuscript (include quotes in quotation marks "like this" to indicate direct quotes from your manuscript), or elaborate on this item by providing additional information not in the ms, or briefly explain why the item is not applicable/relevant for your study

N/A

19-ii) Include qualitative feedback from participants or observations from staff/researchers

Include qualitative feedback from participants or observations from staff/researchers, if available, on strengths and shortcomings of the application, especially if they point to unintended/unexpected effects or uses. This includes (if available) reasons for why people did or did not use the application as intended by the developers.

	1	2	3	4	5	
subitem not at all important	○	○	○	○	○	essential

Does your paper address subitem 19-ii?

Copy and paste relevant sections from the manuscript (include quotes in quotation marks "like this" to indicate direct quotes from your manuscript), or elaborate on this item by providing additional information not in the ms, or briefly explain why the item is not applicable/relevant for your study

N/A

DISCUSSION

22) Interpretation consistent with results, balancing benefits and harms, and considering other relevant

evidence

NPT: In addition, take into account the choice of the comparator, lack of or partial blinding, and unequal expertise of care providers or centers in each group

22-i) Restate study questions and summarize the answers suggested by the data, starting with primary outcomes and process outcomes (use)

Restate study questions and summarize the answers suggested by the data, starting with primary outcomes and process outcomes (use).

	1	2	3	4	5	
subitem not at all important	○	○	○	○	○	essential

Does your paper address subitem 22-i? *

Copy and paste relevant sections from the manuscript (include quotes in quotation marks "like this" to indicate direct quotes from your manuscript), or elaborate on this item by providing additional information not in the ms, or briefly explain why the item is not applicable/relevant for your study

Discussion
Summary of Principal Results
This RCT examined whether being part of an online community would improve self-reported asthma adherence through the mechanisms of role modeling, social support, and website exposure. Being part of an online community for asthma patients for nine weeks failed to increase medication adherence to ICS preventer therapy compared to a diary (P = .32). In addition, there was significantly lower site adherence in the intervention group than in the control (P < .001), even from the very first week (Figure 2). Study condition predicted attrition, where participants were less likely to complete the study if they were randomized to the intervention compared to control condition (P = .03). Further examination indicated that both baseline SMAQ scores (P = .036) and gender (P = .008) were related to attrition with completers being more likely to be women (79.6% versus 61.1%), and less adherent to preventers than non-completers (M = 1.49 versus 1.41). This finding can perhaps be explained by the site being more useful for people struggling with asthma preventer adherence, and less so for people without problems. Such an interpretation would be consistent with Magnezi et al [16], and their study of patient activation, or the extent individuals are able to manage their own health care. The authors found a negative relationship between patient activation and perceived usefulness of the site, as taking a less active role in one's own medical care predicted higher website usefulness.
Primary Outcomes
The online community did not improve preventer adherence compared to the control. This finding is consistent with findings by Eysenbach et al [31] where communities were not associated with improved health outcomes, and more recently Richardson et al [9] where membership in online communities had no effect on behavioral change. The current evidence would suggest that joining an online community intervention is not associated with improved health behavior.
Site Adherence over Nine Weeks
Beginning in the first week of the study, there was a significant difference between conditions for Site Adherence. One possible explanation could be that participants did not like posting their preventer use in an online community, compared to the participants posting in an online diary. Perhaps these participants felt worried or uncomfortable posting this information publicly. On the one hand, an online diary could [illegible]

maintain a sense of privacy, on the other hand, the more rapid decline in the diary might be explained by a lack of engagement in the diary condition over time. Engagement could have remained fairly consistent in the online community because of the presence of other members. Such an explanation would also be consistent with the findings of Richardson et al [9], where an online community was found to reduce attrition to an Internet-mediated walking program, but did not increase walking step count.

Study Strengths and Weaknesses

This study had a number of strengths and weaknesses. In relation to strengths, first, this study tested the effect of membership in an online community as a single intervention component in a RCT. RCTs are the gold standard for determining the effect of an intervention on an outcome. Second, previous studies have also attempted to influence the member activity of online communities through various time-intensive posting strategies, possibly confounding the results [9, 31]. In contrast, this study did not attempt to influence participation beyond the weekly reminders sent to participants in both conditions. Outside of a research context, it is improbable that organizations seeking to enhance health behaviors would divert considerable resources towards encouraging participation. As such, this study represents a test of a deliverable intervention. Third, the inclusion criteria for participation were broad, including a large percentage of adults managing their asthma with a preventer who could possibly benefit from improved preventer adherence. This increases the generalizability of the findings to a large percentage of individuals with asthma.

In relation to weaknesses, first, the only validated measure of adherence was a self-report measure. Several studies have shown that self-report measures of adherence can be unreliable [32, 33]. Second, the Site Adherence should also be interpreted with caution. It is not an objective measure of actual preventer adherence, but more likely a measure of adherence to the intervention instructions. It is possible that actual preventer adherence was different than what was reported by participants on either site. Such an explanation would be supported by the SMAQ-T2 ITT and SMAQ-T2 means (Table 1), which indicated that there were no significant differences in self-report adherence for either condition at follow-up.

Future Directions

The present findings indicate that a “pure” online community does not improve medication adherence. Future research may wish to experiment with multiple levels of engagement. For example, researchers may have a “pure” community in the comparator, and a community with a virtual coach in the intervention condition. As self-report measures of adherence are sometime unreliable, researchers may wish to invest in a mechanical or digital measure of adherence able to provide objective ICS preventer data.

Conclusions

An online community did not improve adherence to asthma preventer medicine. Surprisingly, participants were much more adherent to the control condition than the intervention, although over time it appeared that the difference between conditions attenuated. Without greater community support beyond the existence of the place itself, it does not seem that an online community alone can improve adherence. However, our analyses of attrition suggests that online communities may be more useful to patients with poor asthma adherence than patients with good adherence.

22-ii) Highlight unanswered new questions, suggest future research

Highlight unanswered new questions, suggest future research.

	1	2	3	4	5	
subitem not at all important	○	○	○	○	○	essential

Does your paper address subitem 22-ii?

Copy and paste relevant sections from the manuscript (include quotes in quotation marks "like this" to indicate direct quotes from your manuscript), or elaborate on this item by providing additional information not in the ms, or briefly explain why the item is not applicable/relevant for your study

Discussion
Summary of Principal Results
This RCT examined whether being part of an online community would improve self-reported asthma adherence through the mechanisms of role modeling, social support, and website exposure. Being part of an online community for asthma patients for nine weeks failed to increase medication adherence to ICS preventer therapy compared to a diary (P = .32). In addition, there was significantly lower site adherence in the intervention group than in the control (P < .001), even from the very first week (Figure 2). Study condition predicted attrition, where participants were less likely to complete the study if they were randomized to the intervention compared to control condition (P = .03). Further examination indicated that both baseline SMAQ scores (P = .036) and gender (P = .008) were related to attrition with completers being more likely to be women (79.6% versus 61.1%), and less adherent to preventers than non-completers (M = 1.49 versus 1.41). This finding can perhaps be explained by the site being more useful for people struggling with asthma preventer adherence, and less so for people without problems. Such an interpretation would be consistent with Magnezi et al [16], and their study of patient activation, or the extent individuals are able to manage their own health care. The authors found a negative relationship between patient activation and perceived usefulness of the site, as taking a less active role in one's own medical care predicted higher website usefulness.
Primary Outcomes
The online community did not improve preventer adherence compared to the control. This finding is consistent with findings by Eysenbach et al [31] where communities were not associated with improved health outcomes, and more recently Richardson et al [9] where membership in online communities had no effect on behavioral change. The current evidence would suggest that joining an online community intervention is not associated with improved health behavior.
Site Adherence over Nine Weeks
Beginning in the first week of the study, there was a significant difference between conditions for Site Adherence. One possible explanation could be that participants did not like posting their preventer use in an online community, compared to the participants posting in an online diary. Perhaps these participants felt worried or uncomfortable posting this information publicly. On the one hand, an online diary could maintain a sense of privacy; on the other hand, the more rapid decline in the diary might be explained by a lack of engagement in the diary condition over time. Engagement could have remained fairly consistent in the online community because of the presence of other members. Such an explanation would also be consistent with the findings of [illegible]

Richardson et al [9], where an online community was found to reduce attrition to an Internet-mediated walking program, but did not increase walking step count.

Study Strengths and Weaknesses

This study had a number of strengths and weaknesses. In relation to strengths, first, this study tested the effect of membership in an online community as a single intervention component in a RCT. RCTs are the gold standard for determining the effect of an intervention on an outcome. Second, previous studies have also attempted to influence the member activity of online communities through various time-intensive posting strategies, possibly confounding the results [9, 31]. In contrast, this study did not attempt to influence participation beyond the weekly reminders sent to participants in both conditions. Outside of a research context, it is improbable that organizations seeking to enhance health behaviors would divert considerable resources towards encouraging participation. As such, this study represents a test of a deliverable intervention. Third, the inclusion criteria for participation were broad, including a large percentage of adults managing their asthma with a preventer who could possibly benefit from improved preventer adherence. This increases the generalizability of the findings to a large percentage of individuals with asthma.

In relation to weaknesses, first, the only validated measure of adherence was a self-report measure. Several studies have shown that self-report measures of adherence can be unreliable [32, 33]. Second, the Site Adherence should also be interpreted with caution. It is not an objective measure of actual preventer adherence, but more likely a measure of adherence to the intervention instructions. It is possible that actual preventer adherence was different than what was reported by participants on either site. Such an explanation would be supported by the SMAQ-T2 ITT and SMAQ-T2 means (Table 1), which indicated that there were no significant differences in self-report adherence for either condition at follow-up.

Future Directions

The present findings indicate that a “pure” online community does not improve medication adherence. Future research may wish to experiment with multiple levels of engagement. For example, researchers may have a “pure” community in the comparator, and a community with a virtual coach in the intervention condition. As self-report measures of adherence are sometime unreliable, researchers may wish to invest in a mechanical or digital measure of adherence able to provide objective ICS preventer data.

Conclusions

An online community did not improve adherence to asthma preventer medicine. Surprisingly, participants were much more adherent to the control condition than the intervention, although over time it appeared that the difference between conditions attenuated. Without greater community support beyond the existence of the place itself, it does not seem that an online community alone can improve adherence. However, our analyses of attrition suggests that online communities may be more useful to patients with poor asthma adherence than patients with good adherence.

20) Trial limitations, addressing sources of potential bias, imprecision, and, if relevant, multiplicity of analyses

20-i) Typical limitations in ehealth trials

Typical limitations in ehealth trials: Participants in ehealth trials are rarely blinded. Ehealth trials often look at a multiplicity of outcomes, increasing risk for a Type I error. Discuss biases due to non-use of the intervention/usability issues, biases through informed consent procedures, unexpected events.

	1	2	3	4	5	
subitem not at all important	○	○	○	○	○	essential

Does your paper address subitem 20-i? *

Copy and paste relevant sections from the manuscript (include quotes in quotation marks "like this" to indicate direct quotes from your manuscript), or elaborate on this item by providing additional information not in the ms, or briefly explain why the item is not applicable/relevant for your study

Discussion
Summary of Principal Results
This RCT examined whether being part of an online community would improve self-reported asthma adherence through the mechanisms of role modeling, social support, and website exposure. Being part of an online community for asthma patients for nine weeks failed to increase medication adherence to ICS preventer therapy compared to a diary (P = .32). In addition, there was significantly lower site adherence in the intervention group than in the control (P < .001), even from the very first week (Figure 2). Study condition predicted attrition, where participants were less likely to complete the study if they were randomized to the intervention compared to control condition (P = .03). Further examination indicated that both baseline SMAQ scores (P = .036) and gender (P = .008) were related to attrition with completers being more likely to be women (79.6% versus 61.1%), and less adherent to preventers than non-completers (M = 1.49 versus 1.41). This finding can perhaps be explained by the site being more useful for people struggling with asthma preventer adherence, and less so for people without problems. Such an interpretation would be consistent with Magnezi et al [16], and their study of patient activation, or the extent individuals are able to manage their own health care. The authors found a negative relationship between patient activation and perceived usefulness of the site, as taking a less active role in one's own medical care predicted higher website usefulness.
Primary Outcomes
The online community did not improve preventer adherence compared to the control. This finding is consistent with findings by Eysenbach et al [31] where communities were not associated with improved health outcomes, and more recently Richardson et al [9] where membership in online communities had no effect on behavioral change. The current evidence would suggest that joining an online community intervention is not associated with improved health behavior.
Site Adherence over Nine Weeks
Beginning in the first week of the study, there was a significant difference between conditions for Site Adherence. One possible explanation could be that participants did not like posting their preventer use in an online community, compared to the participants posting in an online diary. Perhaps these participants felt worried or uncomfortable
[illegible]

posting this information publicly. On the one hand, an online diary could maintain a sense of privacy; on the other hand, the more rapid decline in the diary might be explained by a lack of engagement in the diary condition over time. Engagement could have remained fairly consistent in the online community because of the presence of other members. Such an explanation would also be consistent with the findings of Richardson et al [9], where an online community was found to reduce attrition to an Internet-mediated walking program, but did not increase walking step count.

Study Strengths and Weaknesses

This study had a number of strengths and weaknesses. In relation to strengths, first, this study tested the effect of membership in an online community as a single intervention component in a RCT. RCTs are the gold standard for determining the effect of an intervention on an outcome. Second, previous studies have also attempted to influence the member activity of online communities through various time-intensive posting strategies, possibly confounding the results [9, 31]. In contrast, this study did not attempt to influence participation beyond the weekly reminders sent to participants in both conditions. Outside of a research context, it is improbable that organizations seeking to enhance health behaviors would divert considerable resources towards encouraging participation. As such, this study represents a test of a deliverable intervention. Third, the inclusion criteria for participation were broad, including a large percentage of adults managing their asthma with a preventer who could possibly benefit from improved preventer adherence. This increases the generalizability of the findings to a large percentage of individuals with asthma.

In relation to weaknesses, first, the only validated measure of adherence was a self-report measure. Several studies have shown that self-report measures of adherence can be unreliable [32, 33]. Second, the Site Adherence should also be interpreted with caution. It is not an objective measure of actual preventer adherence, but more likely a measure of adherence to the intervention instructions. It is possible that actual preventer adherence was different than what was reported by participants on either site. Such an explanation would be supported by the SMAQ-T2 ITT and SMAQ-T2 means (Table 1), which indicated that there were no significant differences in self-report adherence for either condition at follow-up.

Future Directions

The present findings indicate that a “pure” online community does not improve medication adherence. Future research may wish to experiment with multiple levels of engagement. For example, researchers may have a “pure” community in the comparator, and a community with a virtual coach in the intervention condition. As self-report measures of adherence are sometime unreliable, researchers may wish to invest in a mechanical or digital measure of adherence able to provide objective ICS preventer data.

Conclusions

An online community did not improve adherence to asthma preventer medicine. Surprisingly, participants were much more adherent to the control condition than the intervention, although over time it appeared that the difference between conditions attenuated. Without greater community support beyond the existence of the place itself, it does not seem that an online community alone can improve adherence. However, our analyses of attrition suggests that online communities may be more useful to patients with poor asthma adherence than patients with good adherence.

21) Generalisability (external validity, applicability) of the trial findings

NPT: External validity of the trial findings according to the intervention, comparators, patients, and care providers or centers involved in the trial

21-i) Generalizability to other populations

Generalizability to other populations: In particular, discuss generalizability to a general Internet population, outside of a RCT setting, and general patient population, including applicability of the study results for other organizations

	1	2	3	4	5	
subitem not at all important	○	○	○	○	○	essential

Does your paper address subitem 21-i?

Copy and paste relevant sections from the manuscript (include quotes in quotation marks "like this" to indicate direct quotes from your manuscript), or elaborate on this item by providing additional information not in the ms, or briefly explain why the item is not applicable/relevant for your study

Discussion
Summary of Principal Results
This RCT examined whether being part of an online community would improve self-reported asthma adherence through the mechanisms of role modeling, social support, and website exposure. Being part of an online community for asthma patients for nine weeks failed to increase medication adherence to ICS preventer therapy compared to a diary (P = .32). In addition, there was significantly lower site adherence in the intervention group than in the control (P < .001), even from the very first week (Figure 2). Study condition predicted attrition, where participants were less likely to complete the study if they were randomized to the intervention compared to control condition (P = .03). Further examination indicated that both baseline SMAQ scores (P = .036) and gender (P = .008) were related to attrition with completers being more likely to be women (79.6% versus 61.1%), and less adherent to preventers than non-completers (M = 1.49 versus 1.41). This finding can perhaps be explained by the site being more useful for people struggling with asthma preventer adherence, and less so for people without problems. Such an interpretation would be consistent with Magnezi et al [16], and their study of patient activation, or the extent individuals are able to manage their own health care. The authors found a negative relationship between patient activation and perceived usefulness of the site, as taking a less active role in one's own medical care predicted higher website usefulness.
Primary Outcomes
The online community did not improve preventer adherence compared to the control. This finding is consistent with findings by Eysenbach et al [31] where communities were not associated with improved health outcomes, and more recently Richardson et al [9] where membership in online communities had no effect on behavioral change. The current evidence would suggest that joining an online community intervention is not associated with improved health behavior.
Site Adherence over Nine Weeks

Beginning in the first week of the study, there was a significant difference between conditions for Site Adherence. One possible explanation could be that participants did not like posting their preventer use in an online community, compared to the participants posting in an online diary. Perhaps these participants felt worried or uncomfortable posting this information publicly. On the one hand, an online diary could maintain a sense of privacy; on the other hand, the more rapid decline in the diary might be explained by a lack of engagement in the diary condition over time. Engagement could have remained fairly consistent in the online community because of the presence of other members. Such an explanation would also be consistent with the findings of Richardson et al [9], where an online community was found to reduce attrition to an Internet-mediated walking program, but did not increase walking step count.

Study Strengths and Weaknesses

This study had a number of strengths and weaknesses. In relation to strengths, first, this study tested the effect of membership in an online community as a single intervention component in a RCT. RCTs are the gold standard for determining the effect of an intervention on an outcome. Second, previous studies have also attempted to influence the member activity of online communities through various time-intensive posting strategies, possibly confounding the results [9, 31]. In contrast, this study did not attempt to influence participation beyond the weekly reminders sent to participants in both conditions. Outside of a research context, it is improbable that organizations seeking to enhance health behaviors would divert considerable resources towards encouraging participation. As such, this study represents a test of a deliverable intervention. Third, the inclusion criteria for participation were broad, including a large percentage of adults managing their asthma with a preventer who could possibly benefit from improved preventer adherence. This increases the generalizability of the findings to a large percentage of individuals with asthma.

In relation to weaknesses, first, the only validated measure of adherence was a self-report measure. Several studies have shown that self-report measures of adherence can be unreliable [32, 33]. Second, the Site Adherence should also be interpreted with caution. It is not an objective measure of actual preventer adherence, but more likely a measure of adherence to the intervention instructions. It is possible that actual preventer adherence was different than what was reported by participants on either site. Such an explanation would be supported by the SMAQ-T2 ITT and SMAQ-T2 means (Table 1), which indicated that there were no significant differences in self-report adherence for either condition at follow-up.

Future Directions

The present findings indicate that a “pure” online community does not improve medication adherence. Future research may wish to experiment with multiple levels of engagement. For example, researchers may have a “pure” community in the comparator, and a community with a virtual coach in the intervention condition. As self-report measures of adherence are sometime unreliable, researchers may wish to invest in a mechanical or digital measure of adherence able to provide objective ICS preventer data.

Conclusions

An online community did not improve adherence to asthma preventer medicine. Surprisingly, participants were much more adherent to the control condition than the intervention, although over time it appeared that the difference between conditions attenuated. Without greater community support beyond the existence of the place itself, it does not seem that an online community alone can improve adherence. However, our analyses of attrition suggests that online communities may be more [illegible]

useful to patients with poor asthma adherence than patients with good adherence.

21-ii) Discuss if there were elements in the RCT that would be different in a routine application setting

Discuss if there were elements in the RCT that would be different in a routine application setting (e.g., prompts/reminders, more human involvement, training sessions or other co-interventions) and what impact the omission of these elements could have on use, adoption, or outcomes if the intervention is applied outside of a RCT setting.

	1	2	3	4	5	
subitem not at all important	◯	◯	◯	◯	◯	essential

Does your paper address subitem 21-ii?

Copy and paste relevant sections from the manuscript (include quotes in quotation marks "like this" to indicate direct quotes from your manuscript), or elaborate on this item by providing additional information not in the ms, or briefly explain why the item is not applicable/relevant for your study

Discussion
Summary of Principal Results
This RCT examined whether being part of an online community would improve self-reported asthma adherence through the mechanisms of role modeling, social support, and website exposure. Being part of an online community for asthma patients for nine weeks failed to increase medication adherence to ICS preventer therapy compared to a diary (P = .32). In addition, there was significantly lower site adherence in the intervention group than in the control (P < .001), even from the very first week (Figure 2). Study condition predicted attrition, where participants were less likely to complete the study if they were randomized to the intervention compared to control condition (P = .03). Further examination indicated that both baseline SMAQ scores (P = .036) and gender (P = .008) were related to attrition with completers being more likely to be women (79.6% versus 61.1%), and less adherent to preventers than non-completers (M = 1.49 versus 1.41). This finding can perhaps be explained by the site being more useful for people struggling with asthma preventer adherence, and less so for people without problems. Such an interpretation would be consistent with Magnezi et al [16], and their study of patient activation, or the extent individuals are able to manage their own health care. The authors found a negative relationship between patient activation and perceived usefulness of the site, as taking a less active role in one’s own medical care predicted higher website usefulness.
Primary Outcomes
The online community did not improve preventer adherence compared to the control. This finding is consistent with findings by Eysenbach et al [31] where communities were not associated with improved health outcomes, and more recently Richardson et al [9] where membership in online communities had no effect on behavioral change. The current evidence would suggest that joining an online community intervention is not associated with improved health behavior.
Site Adherence over Nine Weeks
[illegible]

Beginning in the first week of the study, there was a significant difference between conditions for Site Adherence. One possible explanation could be that participants did not like posting their preventer use in an online community, compared to the participants posting in an online diary. Perhaps these participants felt worried or uncomfortable posting this information publicly. On the one hand, an online diary could maintain a sense of privacy; on the other hand, the more rapid decline in the diary might be explained by a lack of engagement in the diary condition over time. Engagement could have remained fairly consistent in the online community because of the presence of other members. Such an explanation would also be consistent with the findings of Richardson et al [9], where an online community was found to reduce attrition to an Internet-mediated walking program, but did not increase walking step count.

Study Strengths and Weaknesses

This study had a number of strengths and weaknesses. In relation to strengths, first, this study tested the effect of membership in an online community as a single intervention component in a RCT. RCTs are the gold standard for determining the effect of an intervention on an outcome. Second, previous studies have also attempted to influence the member activity of online communities through various time-intensive posting strategies, possibly confounding the results [9, 31]. In contrast, this study did not attempt to influence participation beyond the weekly reminders sent to participants in both conditions. Outside of a research context, it is improbable that organizations seeking to enhance health behaviors would divert considerable resources towards encouraging participation. As such, this study represents a test of a deliverable intervention. Third, the inclusion criteria for participation were broad, including a large percentage of adults managing their asthma with a preventer who could possibly benefit from improved preventer adherence. This increases the generalizability of the findings to a large percentage of individuals with asthma.

In relation to weaknesses, first, the only validated measure of adherence was a self-report measure. Several studies have shown that self-report measures of adherence can be unreliable [32, 33]. Second, the Site Adherence should also be interpreted with caution. It is not an objective measure of actual preventer adherence, but more likely a measure of adherence to the intervention instructions. It is possible that actual preventer adherence was different than what was reported by participants on either site. Such an explanation would be supported by the SMAQ-T2 ITT and SMAQ-T2 means (Table 1), which indicated that there were no significant differences in self-report adherence for either condition at follow-up.

Future Directions

The present findings indicate that a “pure” online community does not improve medication adherence. Future research may wish to experiment with multiple levels of engagement. For example, researchers may have a “pure” community in the comparator, and a community with a virtual coach in the intervention condition. As self-report measures of adherence are sometime unreliable, researchers may wish to invest in a mechanical or digital measure of adherence able to provide objective ICS preventer data.

Conclusions

An online community did not improve adherence to asthma preventer medicine. Surprisingly, participants were much more adherent to the control condition than the intervention, although over time it appeared that the difference between conditions attenuated. Without greater community support beyond the existence of the place itself, it does not seem that an online community alone can improve adherence. However, our analyses of attrition suggests that online communities

OTHER INFORMATION

23) Registration number and name of trial registry

Does your paper address CONSORT subitem 23? *

Copy and paste relevant sections from the manuscript (include quotes in quotation marks "like this" to indicate direct quotes from your manuscript), or elaborate on this item by providing additional information not in the ms, or briefly explain why the item is not applicable/relevant for your study

Trial Registration Number:
ISRCTN trial registration number 29399269

24) Where the full trial protocol can be accessed, if available

Does your paper address CONSORT subitem 24? *

Cite a Multimedia Appendix, other reference, or copy and paste relevant sections from the manuscript (include quotes in quotation marks "like this" to indicate direct quotes from your manuscript), or elaborate on this item by providing additional information not in the ms, or briefly explain why the item is not applicable/relevant for your study

N/A

25) Sources of funding and other support (such as supply of drugs), role of funders

Does your paper address CONSORT subitem 25? *

Copy and paste relevant sections from the manuscript (include quotes in quotation marks "like this" to indicate direct quotes from your manuscript), or elaborate on this item by providing additional information not in the ms, or briefly explain why the item is not applicable/relevant for your study

Acknowledgements
We would first like to thank the participants for their willingness and interest in this study. This study was funded by a pilot grant from the University of Leeds School of Psychology. A Fulbright Scholarship from the US-UK Fulbright Commission supported the first author. Finally we would also like to thank the Buddypress open-source community for their support in the development of the intervention.
Conflicts of Interest
None declared.

X27) Conflicts of Interest (not a CONSORT item)

X27-i) State the relation of the study team towards the system being evaluated
In addition to the usual declaration of interests (financial or otherwise), also state the relation of the study team towards the system being evaluated, i.e., state if the authors/evaluators are distinct from or identical with the developers/sponsors of the intervention.

	1	2	3	4	5	
subitem not at all important	○	○	○	○	○	essential

Does your paper address subitem X27-i?
Copy and paste relevant sections from the manuscript (include quotes in quotation marks "like this" to indicate direct quotes from your manuscript), or elaborate on this item by providing additional information not in the ms, or briefly explain why the item is not applicable/relevant for your study

None declared.

About the CONSORT EHEALTH checklist

As a result of using this checklist, did you make changes in your manuscript? *

- ○ yes, major changes
- ◉ yes, minor changes
- ○ no

What were the most important changes you made as a result of using this checklist?

More precision in reporting.

How much time did you spend on going through the checklist INCLUDING making changes in your manuscript *

8 hours because of issu

As a result of using this checklist, do you think your manuscript has improved? *

- (x) yes
- ( ) no
- ( ) Other:

Would you like to become involved in the CONSORT EHEALTH group?

This would involve for example becoming involved in participating in a workshop and writing an "Explanation and Elaboration" document

- ( ) yes
- (x) no
- ( ) Other:

Any other comments or questions on CONSORT EHEALTH

Please make sure that this form can submit properly - have to do this three times now.

STOP - Save this form as PDF before you click submit

To generate a record that you filled in this form, we recommend to generate a PDF of this page (on a Mac, simply select "print" and then select "print as PDF") before you submit it.

When you submit your (revised) paper to JMIR, please upload the PDF as supplementary file.

Don't worry if some text in the textboxes is cut off, as we still have the complete information in our database. Thank you!

Final step: Click submit !

Click submit so we have your answers in our database!

Submit

Never submit passwords through Google Forms.

Powered by

This content is neither created nor endorsed by Google.

Report Abuse - Terms of Service - Additional Terms
