# Supplementary material for: A Web-Based and Mobile Health Social Support Intervention to Promote Adherence to Inhaled Asthma Medications: Randomized Controlled Trial
Source: J Med Internet Res. 2016 Jun 13;18(6):e122. doi: 10.2196/jmir.4963 (PMC4923591; doi:10.2196/jmir.4963)
Supplement: Multimedia Appendix 2 [file jmir_v18i6e122_app2.pdf]

Dear Sir or Madam:

We are researchers at the University of Leeds, Institute of Psychological Sciences, conducting an important, systematic trial of a web application that aims to improve the lives of people suffering with asthma. We have received ethical approval for our study (ethics 13-0096).

Would you be kind enough to forward the below request to any relevant email lists?

Thank you,

Justin Koufopoulos, Mark Connor, Peter Gardner, and Ian Kellar
University of Leeds
Institute of Psychological Sciences

Subject: £20 Love-to-Shop Voucher for participation in Asthma Web-based experiment!

Hi there,

Do you have asthma and manage it with a preventer? If so, you may be eligible to participate in an innovative web-based study.

We invite you to participate in an exciting new research project using a website and mobile application designed to help you better manage your asthma. Individuals who participate in this study will be given a £20 Love-to-Shop Voucher upon completion!

If you are interested in participating in this study, please fill out this short survey and provide your email address at the end:
https://qtrial.qualtrics.com/SE/?SID=SV_cHogB6MBS3TPZ2J

Thank you!

Justin Koufopoulos
Institute of Psychological Sciences
psjtk@leeds.ac.uk
