# Supplementary material for: A Web-Based and Mobile Health Social Support Intervention to Promote Adherence to Inhaled Asthma Medications: Randomized Controlled Trial
Source: J Med Internet Res. 2016 Jun 13;18(6):e122. doi: 10.2196/jmir.4963 (PMC4923591; doi:10.2196/jmir.4963)
Supplement: Multimedia Appendix 3 [file jmir_v18i6e122_app3.pdf]

Are you a student (undergraduate or postgraduate) at the University of Leeds?	Yes	No
Do you have asthma?	Yes	No
Have you previously taken the study run by Justin Koufopoulos entitled 'AsthmaVillage'?	Yes	No
How many times a day are you prescribed your preventer inhaler? For example, if you are prescribed to use your preventer for two puffs, twice per day, you would put the number '4'. Puffs prescribed per day:	(text box)	
Do you have a smartphone (i.e., a phone that can access the internet and download apps)? Examples include the iPhone or Android.	Yes	No
For this study, you will be required to use a web application, which will take a very small amount of data to run.<br><br>Would you be willing to use your phone's data plan and/or a Wi-Fi (wireless) internet connection to access this application? You can select multiple responses for this question.		
I would be willing to use…(select all that apply)	Data	
	Wi-Fi	
	Neither	
If you are interested in participating in this study, please provide your email address in the text box below. We will be in touch shortly about participation in the study, and send you additional important information. Thank you.		
