# Supplementary material for: A Web-Based and Mobile Health Social Support Intervention to Promote Adherence to Inhaled Asthma Medications: Randomized Controlled Trial
Source: J Med Internet Res. 2016 Jun 13;18(6):e122. doi: 10.2196/jmir.4963 (PMC4923591; doi:10.2196/jmir.4963)
Supplement: Multimedia Appendix 4 [file jmir_v18i6e122_app4.pdf]

Participant Information Form

Online Applications and Asthma Management

What is this study about?

We invite you to participate in a research project about the usability and usefulness of a prototype online web app for asthma management. This study is being conducted as part of my, Justin Koufopoulos', programme of research in the Institute of Psychological Sciences.

Do I have to take part?

This information sheet has been written to help you decide if you would like to take part. It is up to you and you alone whether or not to take part. If you do decide to take part you will be free to withdraw at any time without providing a reason, up until the anonymization of data. After the anonymization of data, all links between identity and data will be lost.

Are there any restrictions on who can take part?

You must have asthma, a smartphone with a wireless connection or data plan, and be managing your asthma with an inhaler (preventer). You should also be in a position to give informed consent to participate in the study (e.g., 18 years or over). If you have any questions about eligibility or consent, please contact Justin Koufopoulos at psjtk@leeds.ac.uk.

What would I be required to do?

You will be asked to track your daily preventer use using a web application posting as you use your preventer inhaler. This can be done via the mobile site, or on a personal computer. We would ask that you make full use of the website and all of its features so as to get the full experience of the site! You will also be required to read the directions for how to download and access the application as well as guidelines for use.

We would expect that you will be using the application/website at least daily, but there is no limit to the number of visits (and length of time) you may wish to access the application.

At the end of the study, you will be asked to fill out a questionnaire that will take approximately 15-20 minutes to complete on your experience with the site and your asthma control.

Will my participation be Anonymous and Confidential?

Your data will be kept strictly confidential. Your permission will be sought in the Participant Consent Form (the next page) for the data you provide to be used for future scholarly purposes. No record of your Internet Protocol address or any personally

identifiable information will be recorded and you will never be personally identifiable from the study data you provide or the publications that result from it.

Storage and destruction of data collected

Your anonymised, confidential data will be stored for 5 years.

What will happen to the results of the research study?

The results will be finalised by December 2013 and may contribute to a journal article on the experiment and/or grant proposals on asthma management.

Reward

Participants who complete the study will received a Love-to-Shop voucher for £20.

Are there any potential risks to taking part?

None.

Consent and approval

This research proposal has been scrutinized and been granted Ethical Approval through the University of Leeds ethical approval process.

Questions

If you have any questions or concerns about this study, please e-mail Justin Koufopoulos: psjtk@leeds.ac.uk
