# Supplementary material for: A Web-Based and Mobile Health Social Support Intervention to Promote Adherence to Inhaled Asthma Medications: Randomized Controlled Trial
Source: J Med Internet Res. 2016 Jun 13;18(6):e122. doi: 10.2196/jmir.4963 (PMC4923591; doi:10.2196/jmir.4963)
Supplement: Multimedia Appendix 5 [file jmir_v18i6e122_app5.pdf]

Participant Consent Form

The University of Leeds attaches high priority to the ethical conduct of research. We therefore ask that you consider the following points before approving this form. Pressing ‘Continue’ confirms that you are happy to participate in this study.

Consent

The purpose of this form is to ensure that you are willing to take part in this study and to let you understand what it entails. Approving this form does not commit you to anything you do not wish to do.

Material gathered during this research will be anonymous, so it is impossible to trace it back to you. Once it is removed from the server, it will be stored for an indefinite period. Please answer each statement concerning the collection and use of the research data.

I have read and understood the Participant Information Page.	Yes	No
I understand that I can email the lead researcher, Justin Koufopoulos (psjtk@leeds.ac.uk) if I have any questions about the study	Yes	No
I understand that I can withdraw from the study at any time by emailing Justin Koufopoulos (psjtk@leeds.ac.uk)	Yes	No
I give permission for members of the research team to have access to my anonymized responses. I understand that my name will not be linked with the research materials, and I will not be identified or identifiable in the report or reports that result from the research.	Yes	No
I agree to my data (in line with the conditions outlined above) being used to prepare scientific manuscripts and grant funding applications and to my data being archived and used for further research projects by Justin Koufopoulos.	Yes	No
I have been made fully aware of the potential risks associated with this research and am satisfied with the information provided.	Yes	No
I am aged 18 or over and agree to take part in the study.	Yes	No
