# Supplementary material for: A Web-Based and Mobile Health Social Support Intervention to Promote Adherence to Inhaled Asthma Medications: Randomized Controlled Trial
Source: J Med Internet Res. 2016 Jun 13;18(6):e122. doi: 10.2196/jmir.4963 (PMC4923591; doi:10.2196/jmir.4963)
Supplement: Multimedia Appendix 6 [file jmir_v18i6e122_app6.pdf]

<table>
<tr><td colspan="9">Demographics questionnaire</td></tr>
<tr><td>Are you male or female?</td><td>Male</td><td colspan="3">Female</td><td colspan="4">Prefer not to say</td></tr>
<tr><td>What is your age?</td><td colspan="8">[text box]</td></tr>
<tr><td>On an average day, about how frequently do you visit social networking sites such as Facebook, Google +, or Twitter?</td><td>I never use social networking sites</td><td>1 to 4 times per day</td><td>5 to 10 times per day</td><td>11 to 15 times per day</td><td>16 to 20 times per day</td><td>21 to 25 times per day</td><td>More than 25 times per day</td><td>Prefer not to say</td></tr>
<tr><td>On an average day, about how much time do you spend visiting social networking sites such as Facebook, Google +, or Twitter?</td><td>I never use social networking sites</td><td>1 to 2 hours per day</td><td>2 to 3 hours per day</td><td>3 to 4 hours per day</td><td>4 to 5 hours per day</td><td>More than 5 hours per day</td><td colspan="2">Prefer not to say</td></tr>
<tr><td>Is English your first language?</td><td colspan="8">Yes/No</td></tr>
<tr><td>What is your email address? This email address must be the same email address that you will use to register for AsthmaVillage.</td><td colspan="8">[text box]</td></tr>
<tr><td colspan="9">A reliever is an inhaler (often blue in colour) which contains bronchodilator drugs</td></tr>
<tr><td>In a typical week, excluding before exercise, how frequently do you use your reliever (in occasions) to relieve asthma symptoms?</td><td colspan="8">[text box]</td></tr>
<tr><td>In a typical week, excluding before exercise, how many days do you go without having to use your reliever?</td><td colspan="8">[text box]</td></tr>
</table>

A preventer is an inhaler (often brown, red, yellow, or orange in colour) which usually contains a steroid drug.	
How many puffs per day are you prescribed to use your preventer?	[text box]
In the past month, about what percentage of the time do you use your preventer as prescribed?	[text box]
