# Supplementary material for: A Web-Based and Mobile Health Social Support Intervention to Promote Adherence to Inhaled Asthma Medications: Randomized Controlled Trial
Source: J Med Internet Res. 2016 Jun 13;18(6):e122. doi: 10.2196/jmir.4963 (PMC4923591; doi:10.2196/jmir.4963)
Supplement: Multimedia Appendix 7 [file jmir_v18i6e122_app7.pdf]

Question	Patient Answer	
Do you ever forget to take your ASTHMA PREVENTER MEDICATION?	Yes	No
Are you careless at times about taking your ASTHMA PREVENTER MEDICATION?	Yes	No
When you feel better do you sometimes stop taking your ASTHMA PREVENTER MEDICATION?	Yes	No
Thinking about the last week, how often have you not taken your ASTHMA PREVENTER MEDICINE AS PRESCRIBED?	Never<br>1+/- 2 times<br>3+/- 5 times<br>6+/- 10 times<br>>10 times	
Did you not take any of your ASTHMA PREVENTER MEDICINE over the past weekend?	Yes	No
Over the past 3 months, how many days have you not taken any of your ASTHMA PREVENTER MEDICINE at all?	Greater than or equal to 2 days;<br>Less than 2 days.	

SCORING: A positive response to any of the qualitative questions, more than two doses missed over the past week, or over 2 days of total non-medication during the past 3 months = nonadherent.
