# Supplementary figures and images for: A Web-Based and Mobile Health Social Support Intervention to Promote Adherence to Inhaled Asthma Medications: Randomized Controlled Trial
Source: J Med Internet Res. 2016 Jun 13;18(6):e122. doi: 10.2196/jmir.4963 (PMC4923591; doi:10.2196/jmir.4963)

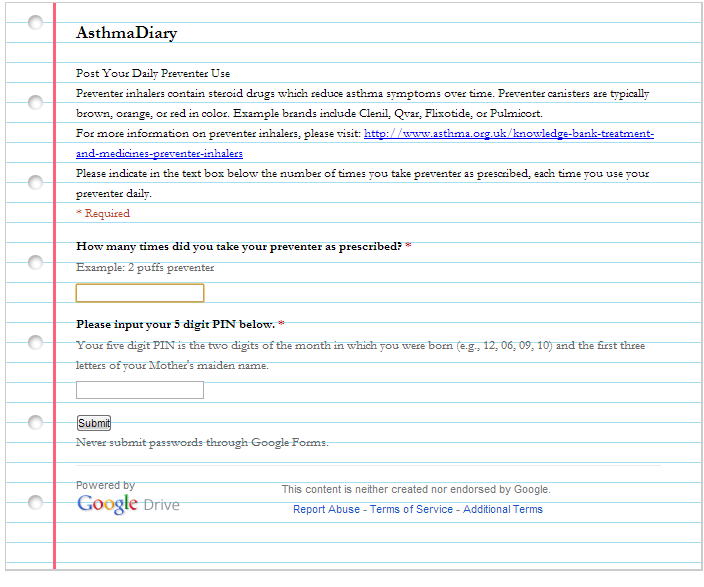

Supplement: Multimedia Appendix 9 [file jmir_v18i6e122_app9.png]
