# Supplementary material for: A Web-Based and Mobile Health Social Support Intervention to Promote Adherence to Inhaled Asthma Medications: Randomized Controlled Trial
Source: J Med Internet Res. 2016 Jun 13;18(6):e122. doi: 10.2196/jmir.4963 (PMC4923591; doi:10.2196/jmir.4963)
Supplement: Multimedia Appendix 10 [file jmir_v18i6e122_app10.pdf]

Getting Started with AsthmaDiary

AsthmaDiary is a way to easily log your daily preventer use. AsthmaDiary can be used on Mac or PC, iPhone or Android.

In order to receive credit for this study and receive the £20 Love-to-Shop Voucher, participants must at minimum post their asthma preventer use weekly.

Logging Preventer Use

Logging preventer use is easy! When you receive the link to the site (at the end of this instruction guide) and use your preventer inhaler, simply type the number of puffs you used your inhaler (for example, 2 puffs) and type "2 puffs preventer" into the form box.

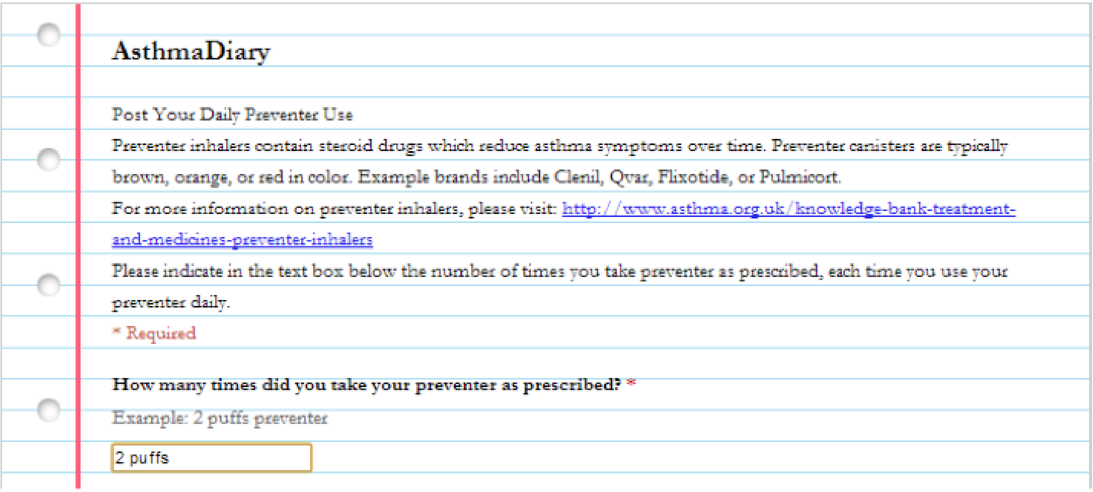

AsthmaDiary

Post Your Daily Preventer Use

Preventer inhalers contain steroid drugs which reduce asthma symptoms over time. Preventer canisters are typically brown, orange, or red in color. Example brands include Clenil, Qvar, Flixotide, or Pulmicort.

For more information on preventer inhalers, please visit: http://www.asthma.org.uk/knowledge-bank-treatment-and-medicines-preventer-inhalers

Please indicate in the text box below the number of times you take preventer as prescribed, each time you use your preventer daily.

* Required

How many times did you take your preventer as prescribed? *

Example: 2 puffs preventer

2 puffs

Then, you will need to input your 5 digit PIN which you created earlier. Your 5 digit PIN is the two digits of the month in which you were born (e.g., 12, 06, 09, 10, etc.), and the first three letters of your Mother's maiden name.

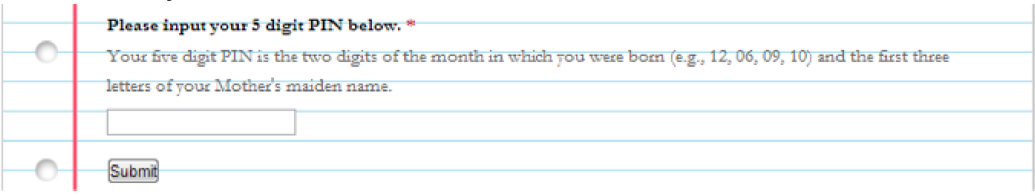

Please input your 5 digit PIN below. *

Your five digit PIN is the two digits of the month in which you were born (e.g., 12, 06, 09, 10) and the first three letters of your Mother's maiden name.

Submit

Adding AsthmaDiary to your Smartphone Device: The Super Easy, Setup Guide

For iPhone

1. In your browser, once you have completed this instructions guide, go to [link].
2. Tap on the ‘Share’ or ‘Action’ button (which looks like an arrow) and then tap ‘Add to Home Screen.’
3. In the ‘Add to Home’ screen, you can edit the title of the icon. Then click ‘Add.’
4. The icon will now appear on your iPhone home screen.

For Android

1. In your browser, once you have completed this instructions guide, go to [link].
2. Tap the menu button, and add the page to your bookmarks. In Google Chrome, just tap on the star and follow the prompt.
3. Open your bookmarks using the menu button, and find the new bookmark you’ve just added. Press down and hold on the bookmark until you see an action menu. Select ‘Add to home screen.’
4. The bookmark will now appear on your Android device home screen.
