# Supplementary material for: A Web-Based and Mobile Health Social Support Intervention to Promote Adherence to Inhaled Asthma Medications: Randomized Controlled Trial
Source: J Med Internet Res. 2016 Jun 13;18(6):e122. doi: 10.2196/jmir.4963 (PMC4923591; doi:10.2196/jmir.4963)
Supplement: Multimedia Appendix 11 [file jmir_v18i6e122_app11.pdf]

Getting Started with AsthmaVillage: The Super Easy, Basic Overview

After you've created your account and completed sign up, it's time to start using AsthmaVillage! AsthmaVillage is an online social network, with many of the same features you are already used to.

AsthmaVillage is supported on PC and Mac, iPhone and Android. After registering, once you've signed into AsthmaVillage, you should bookmark the page and tell AsthmaVillage to remember your username and password.

Updating Your Profile Picture

Updating your profile picture is easy. On the right hand-side of the page, just click on your avatar or username. You'll be taken to your profile with your current avatar at the top and all of your activity below. Click 'Profile' > 'Change Avatar' > 'Browse'. Then select an image file saved to your computer, double click on the file, and click 'Upload Image.'

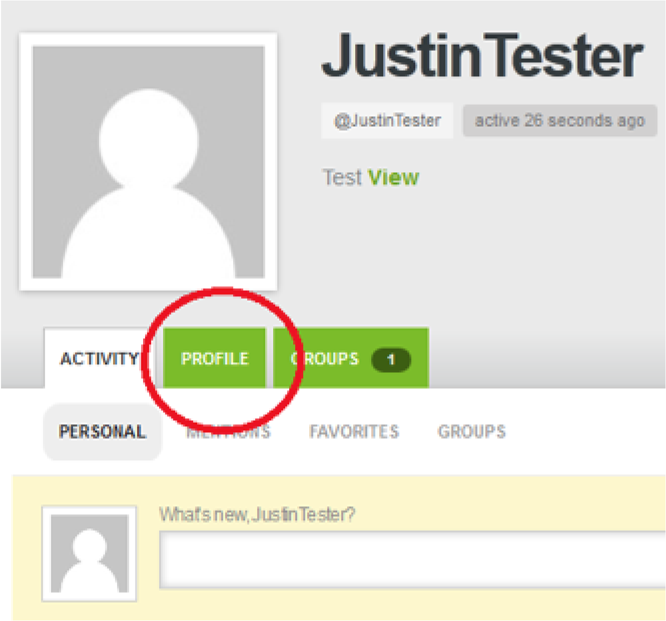

Posting in the Feed

Posting in the main feed, 'AsthmaVillage Newsfeed' in the main menu, is easy. Simply click in the main textbox under "What's new, username?" and type. Then click 'Post Update'.

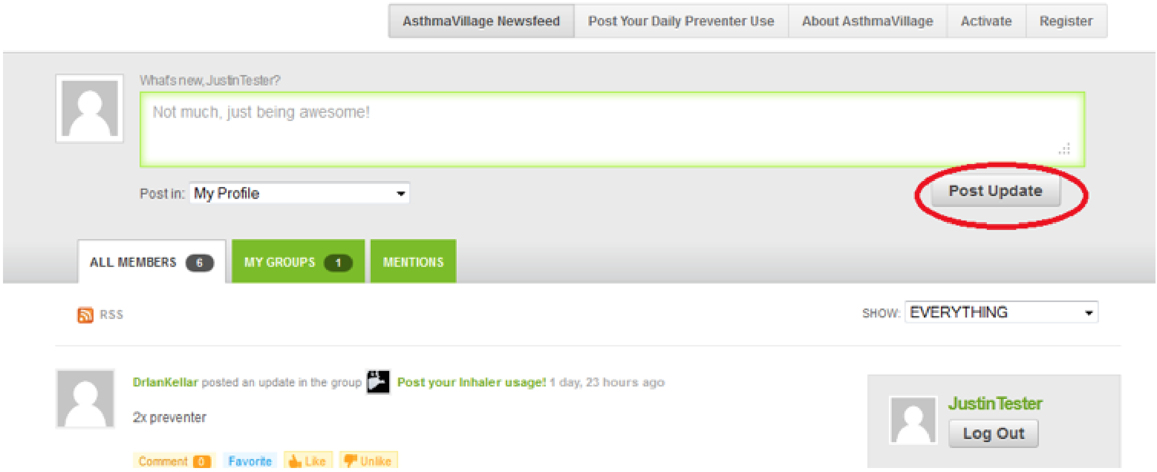

PLEASE READ: Tracking Preventer Use

One of the main features, and goals of the AsthmaVillage, is to help you improve your asthma management through the self-tracking and group posting of preventer use.

This is a very important feature of AsthmaVillage, and we ask that all participants use the following feature.

On every occasion that you use your preventer, please post the number of puffs in the group, "Post Your Daily Preventer Use" (e.g., "2 times preventer").

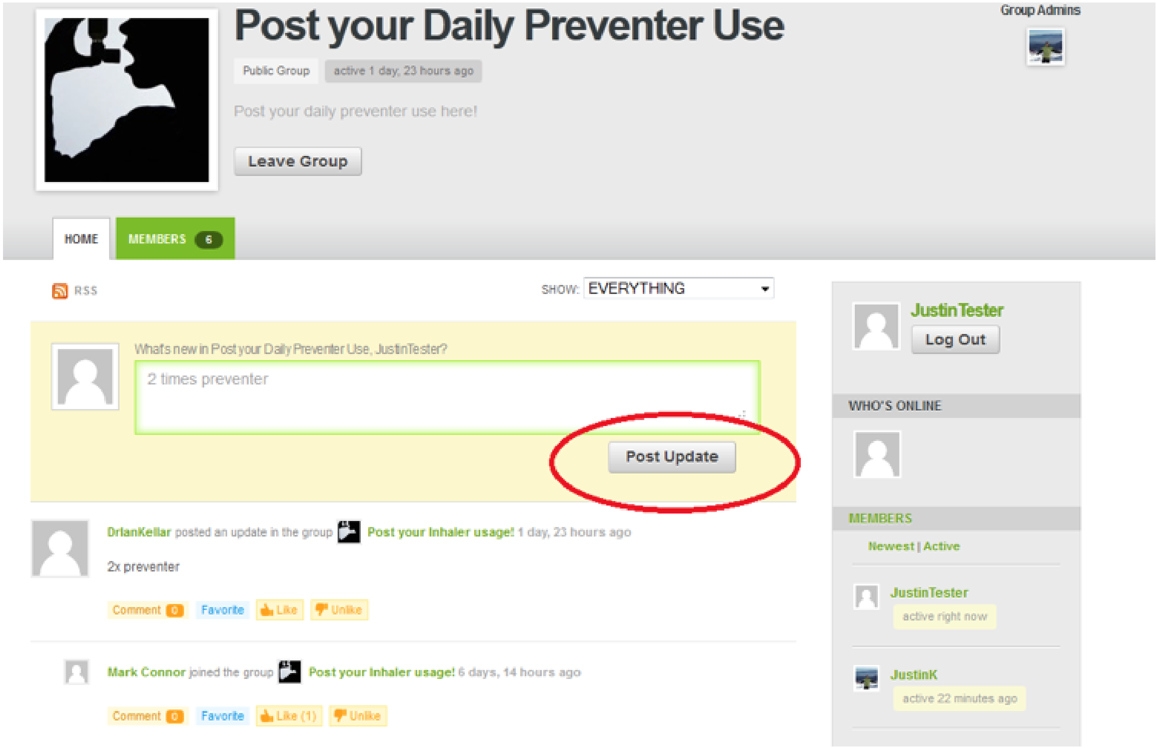

There are several ways to post your preventer usage. You can go to the main group's page, by clicking 'Post Your Daily Preventer Usage' at the top of the page in the main menu. Then, in the Groups Directory, click on the group 'Post your Daily Preventer Use.'

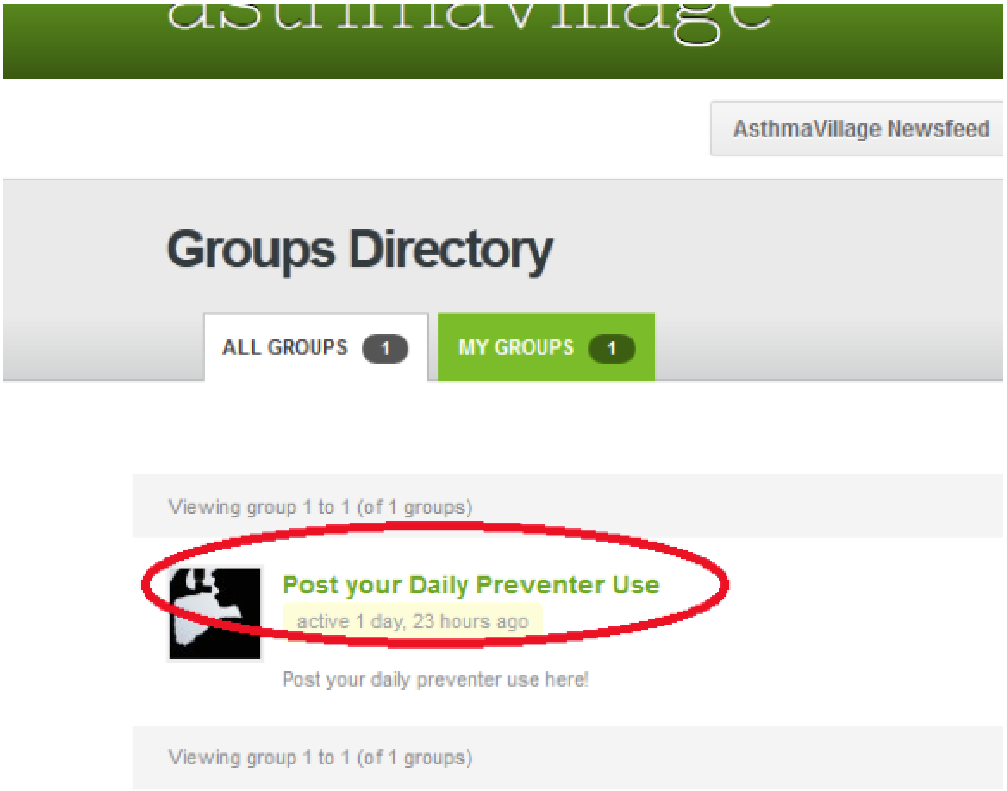

In the main feed, 'AsthmaVillage Newsfeed,' you can see group activity by clicking 'My Groups.' You can post directly from the newsfeed to the group. Create your post, and on the 'Post in' dropdown menu, click "Post your Daily Preventer Use." Then, click 'Post Update'. The update will then appear in both the AsthmaVillage Newsfeed, and the group feed.

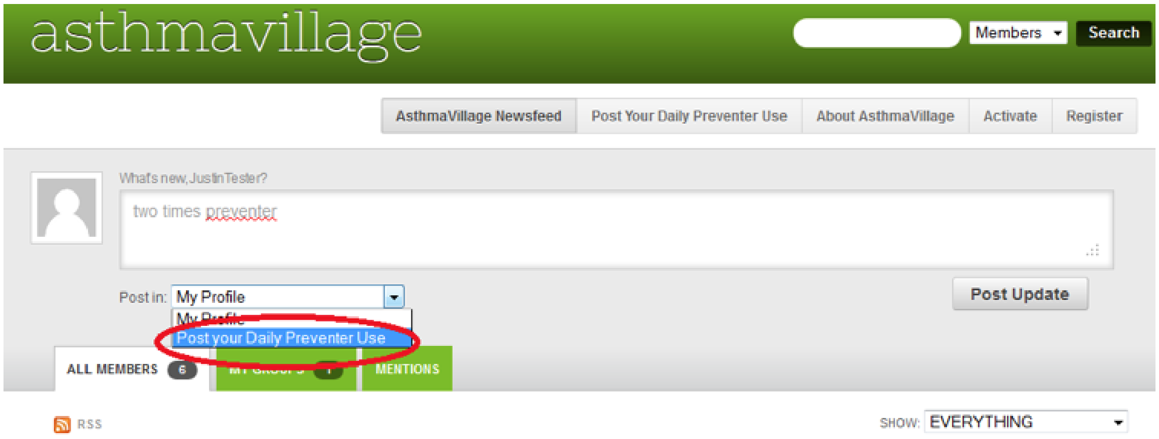

Adding AsthmaVillage to Your Smartphone Device: The Super Easy, Setup Guide

For iPhone

1. In Safari, go to measured-response.com/leeds/. You will see the main activity feed.
2. In the upper-left hand corner, tap ‘Login.’ Enter your username and password, check ‘Remember Me’ and tap ‘Log In.’
3. Logging in will bring you to the Dashboard. Tap ‘AsthmaVillage’ on the Settings bar to bring you to the main site.
4. Tap on the ‘Share’ or ‘Action’ button and then tap ‘Add to Home Screen.’
5. In the ‘Add to Home’ screen, you can edit the title of the icon. Then click ‘Add.’
6. The icon will now appear on your iPhone home screen.

For Android

1. In your browser, go to measured-response.com/leeds/. You will see the main activity feed.
2. Tap the menu button, and add the page to your bookmarks. In Google Chrome, just tap on the star and follow the prompt.
3. Open your bookmarks using the menu button, and find the new bookmark you’ve just added. Press down and hold on the bookmark until you see an action menu. Select ‘Add to home screen.’
4. The bookmark will now appear on your Android device home screen.
