# Supplementary material for: A Web-Based and Mobile Health Social Support Intervention to Promote Adherence to Inhaled Asthma Medications: Randomized Controlled Trial
Source: J Med Internet Res. 2016 Jun 13;18(6):e122. doi: 10.2196/jmir.4963 (PMC4923591; doi:10.2196/jmir.4963)
Supplement: Multimedia Appendix 12 [file jmir_v18i6e122_app12.pdf]

Hello,

By now you should have been using AsthmaVillage for ____ (*week number*) weeks! Have you been noticing any improvements in your asthma symptoms or the extent you take your preventer?

Remember, to get credit for this study and receive the £20 Love-to-Shop Voucher you must post your preventer use at minimum once per week. To get the full effect of AsthmaVillage however, you should post your preventer use every time you use your preventer medicine.

Stay healthy,

Justin

Justin Koufopoulos
2012 – 2013 Fulbright Scholar
University of Leeds Partnership Award
Institute of Psychological Sciences
psjtk@leeds.ac.u k
