# Supplementary material for: A Web-Based and Mobile Health Social Support Intervention to Promote Adherence to Inhaled Asthma Medications: Randomized Controlled Trial
Source: J Med Internet Res. 2016 Jun 13;18(6):e122. doi: 10.2196/jmir.4963 (PMC4923591; doi:10.2196/jmir.4963)
Supplement: Multimedia Appendix 13 [file jmir_v18i6e122_app13.pdf]

Hello,

Thank you for participating in this study of asthma management from the University of Leeds Institute of Psychological Sciences.

In order to complete your participation in this study and receive your £20 Love-to-Shop voucher, you must answer our end-of-study survey by following this link: https://qtrial.qualtrics.com/SE/?SID=SV_71gPyfzjTZR8rcx

The survey will take approximately 30 minutes to compete, and must be completed in a single sitting, so do please ensure you have plenty of time to complete it.

Only individuals who complete this survey will be eligible to receive the £20 Love-to-Shop voucher. At the end of the survey, you will be asked to enter the address where you would like your Love-to-Shop voucher sent. Love-to-Shop vouchers can only be sent to valid UK addresses.

At the end of the survey there will also be a debriefing form explaining the purpose of the study and the storage of data. If you have any additional questions, please contact me, Justin Koufopoulos, for further information.

Thank you for your participation, and as always, stay healthy.

Justin

Justin Koufopoulos
2012 – 2013 Fulbright Scholar
University of Leeds Partnership Award
Institute of Psychological Sciences
psjtk@leeds.ac.uk
